# Chemical element transport in stellar evolution models

**DOI:** 10.1098/rsos.170192

**Published:** 2017-08-23

**Authors:** Maurizio Salaris, Santi Cassisi

**Affiliations:** 1 Astrophysics Research Institute, Liverpool John Moores University, IC2 Liverpool Science Park, 146 Brownlow Hill, L3 5RF, Liverpool, UK; 2 INAF—Osservatorio Astronomico Collurania, Via Mentore Maggini, 64100 Teramo, Italy; 3 Instituto de Astrofísica de Canarias, Via Láctea s/n. E38205 La Laguna, Tenerife, Spain

**Keywords:** stellar physics, mixing, convection, diffusion, rotation

## Abstract

Stellar evolution computations provide the foundation of several methods applied to study the evolutionary properties of stars and stellar populations, both Galactic and extragalactic. The accuracy of the results obtained with these techniques is linked to the accuracy of the stellar models, and in this context the correct treatment of the transport of chemical elements is crucial. Unfortunately, in many respects calculations of the evolution of the chemical abundance profiles in stars are still affected by sometimes sizable uncertainties. Here, we review the various mechanisms of element transport included in the current generation of stellar evolution calculations, how they are implemented, the free parameters and uncertainties involved, the impact on the models and the observational constraints.

## 1. Introduction

Almost a century ago Eddington wrote [1]:

‘It is reasonable to hope that in a not too distant future we shall be competent to understand so simple a thing as a star’.

During this time, the theory of stellar evolution has been developed and established, and its main predictions confirmed by a large number of empirical tests that have involved photometric, spectroscopic and asteroseismic observations (and solar neutrino flux measurements, after the discovery of neutrino oscillations). Results obtained from stellar model computations are nowadays widely used to develop a vast array of techniques to estimate distances, ages, star formation histories and chemical evolution of star clusters and galaxies, both resolved and unresolved [2].

Obtaining this kind of information from techniques rooted in stellar evolution calculations is a crucial step to address problems like understanding the mechanisms that drive the formation and evolution of galaxies. The accuracy of results gathered from stellar population analyses is tied to the accuracy of the current generation of stellar models; in this respect, one particularly thorny issue is how to treat the transport of chemical elements in stellar evolution calculations. The problem is that mixing and element transport do not arise from the solution of the equations of stellar structure and evolution, instead they have to be ‘added’ following recipes that often—as we will see—involve a number of free parameters and/or are subject to sizable uncertainties.

On the other hand, the temporal evolution of the chemical abundance profiles within stellar models is a main evolutionary driver, and can be in principle tested through spectroscopic observations of photospheric abundances of key elements, asteroseismic observations (study of non-radial pulsations of stars, which can test thermodynamical properties of stellar interiors that are also affected by the chemical composition), and more indirectly through the effect on star counts (sensitive to evolutionary timescales) and evolutionary paths in colour-magnitude-diagrams (CMDs) or Hertzsprung–Russell diagrams (HRDs), which are all affected by the internal chemical profiles.

The aim of this review is to discuss the various mechanisms of chemical element transport included in the current generation of stellar models, their effect on the evolutionary properties of the models, and the various prescriptions found in the literature, that often produce very different results. This will allow the reader to appreciate the main uncertainties involved, and what properties of stellar models are affected the most.

We start in §2 with a brief summary of the equations of stellar structure and evolution, and an overview of the main evolutionary properties of models of different initial masses, to set the stage for the discussion that follows. The next sections discuss the implementation in stellar modelling, the associated uncertanties and the observational constraints of processes like convection (§3), semiconvection (§4), thermohaline mixing (§5), atomic diffusion (§6), phase separation upon crystallization (§7) and rotationally induced element transport (§8). Section 9 discusses briefly an example of how a combination of several of the mechanisms discussed in the previous sections can explain the puzzling trend of photospheric Li abundances with effective temperature in open clusters, and conclusions follow in §10.

## 2. Stellar model computation

With *stellar modelling* we mean here the calculation of the run of physical (i.e. luminosity, temperature, density and specific heats) and chemical quantities, from the centre to the photosphere of a star of a given initial mass and chemical composition, and their evolution with time. Despite tremendous advances in computing power and computational techniques during the last decades, a full detailed modelling of a star by solving the equations of radiation hydrodynamics in three dimensions (3D) is still unfeasible, and this will be the case for the foreseeable future. This inability to model stars with multidimensional radiation hydrodynamics is a consequence of the extreme range of spatial and temporal scales^1^ that need to be resolved when calculating full evolutionary models covering all stages from the pre-main sequence (pre-MS) to the last *white dwarf* (WD) or pre-supernova phases. Current hydrodynamics computations are, however, starting to be able to provide some guidelines about mixing processes [3], as highlighted in the following sections.

For these reasons, complete stellar evolution computations still have to rely basically on the following ‘classical’ set of one-dimensional (1D) equations for spherical, non-rotating and non-magnetic stars (equation of continuity of mass, hydrostatic equilibrium,^2^ energy generation and energy transport, respectively) and Raphson–Newton solution methods [4]:

2.1 $$\begin{array}{rl} \frac{\partial r}{\partial m} & =\frac{1}{4\pi r^{2}\rho }, \end{array}$$

2.2 $$\begin{array}{rl} \frac{\partial P}{\partial m} & =-\frac{Gm}{4\pi r^{4}}, \end{array}$$

2.3 $$\begin{array}{rl} \frac{\partial L}{\partial m} & =\epsilon_{n}-\epsilon_{\nu }+\epsilon_{g} \end{array}$$

2.4 $$\begin{array}{rl} \;\text{and}\;\frac{\partial T}{\partial m} & =-\frac{GmT}{4\pi r^{4}P}\nabla , \end{array}$$

where the independent variable *m* is the mass enclosed within radius *r*, and *T*, *L*, *P* and *ρ* are, respectively, the temperature, luminosity, pressure and density at the layer specified by the value of *m*. The coefficient *ϵ*_*ν*_ denotes the energy per unit time and unit mass carried away by neutrinos (that do not interact with the stellar gas), *ϵ*_n_ is the energy per unit time and unit mass produced by nuclear reactions. For a generic nuclear reaction $n_{A}A+n_{b}b\to$products involving elements *A* and *b* with mass fractions *X*_A_ and *X*_b_ and atomic weights *A*_A_ and *A*_b_

$$\epsilon_{n}=R_{\mathrm{Ab}}Q_{\mathrm{Ab}},$$

where *Q*_Ab_ is the amount of energy released by a single reaction and *R*_Ab_ is the number of reactions per unit mass and unit time, given by

2.5 $$R_{\mathrm{Ab}}=\rho^{n_{A}+n_{b}-1}\frac{X_{A}^{n_{A}}X_{b}^{n_{b}}}{A_{A}^{n_{A}}A_{b}^{n_{b}}}\frac{{\left\langle \sigma v\right\rangle }_{\mathrm{Ab}}}{m_{H}^{n_{A}+n_{b}}n_{A}!n_{b}!},$$

where 〈*σv*〉_Ab_ is the reaction cross section.^3^

The coefficient *ϵ*_g_ represents the so-called *gravitational energy* produced per unit time and unit mass, and is given by

2.6 $$-\epsilon_{g}={\left(\frac{\partial U}{\partial v}\right)}_{T,\mu }\frac{\partial v}{\partial t}+{\left(\frac{\partial U}{\partial T}\right)}_{v,\mu }\frac{\partial T}{\partial t}+{\left(\frac{\partial U}{\partial \mu }\right)}_{T,v}\frac{\partial \mu }{\partial t}+P\frac{\partial v}{\partial t},$$

where *U* is the internal energy per unit mass, *v*=1/*ρ* is the specific volume and *μ* is the mean molecular weight of the stellar matter. The term (∂*U*/∂*μ*)_*T*,*v*_(∂*μ*/∂*t*) arises from the variation of *U* at constant temperature and volume due to the change of chemical abundances. Its contribution to the energy budget is negligible when nuclear reactions are efficient, but is very important in the case of WDs, where nuclear burnings are inefficient. When integrated over the whole stellar structure, *ϵ*_g_ is equal to the time variation of the internal energy plus the gravitational potential energy of the star [2,5].

For the case of radiative plus electron conduction energy transport, the gradient ∇≡(d ln⁡(T)/d ln⁡(P)) is set to ∇_rad_

2.7 $$\nabla_{\mathrm{rad}}=\frac{3}{16\pi acG}\frac{\kappa LP}{mT^{4}},$$

where *a* is the radiation density constant, *c* is the speed of light, *G* is the gravitational constant and *κ* is the Rosseland opacity, including also the contribution of electron conduction when appropriate. In the case of convective energy transport, a theory of convection is needed to calculate the appropriate temperature gradient ∇_conv_ (see §3).

These equations are complemented by a set of *I* equations (*s*=1,…,*I*) for the change of the mass fraction of the *I* chemical elements considered at the layer specified by *m*. Consider first the changes due just to nuclear reactions; an element *s* is produced by *w* reactions of the type

$$n_{h}h+n_{k}k\to n_{p}s$$

and destroyed by *l* reactions

$$n_{d}s+n_{j}j\to n_{z}z.$$

This provides the following equation for the variation of the abundance of *s*^4^

2.8 $$\begin{array}{rl} \frac{\partial X_{s}}{\partial t} & =\sum_{w}\rho^{n_{h}+n_{k}-1}n_{p}\frac{X_{h}^{n_{h}}X_{k}^{n_{k}}A_{s}}{A_{h}^{n_{h}}A_{k}^{n_{k}}}\frac{{\left\langle \sigma v\right\rangle }_{hk}}{m_{H}^{n_{h}+n_{k}-1}n_{h}!n_{k}!}\\ & \;-\sum_{l}\rho^{n_{d}+n_{j}-1}n_{d}\frac{X_{s}^{n_{d}}X_{j}^{n_{j}}A_{s}}{A_{s}^{n_{d}}A_{j}^{n_{j}}}\frac{{\left\langle \sigma v\right\rangle }_{sj}}{m_{H}^{n_{d}+n_{j}-1}n_{d}!n_{j}!}. \end{array}$$

The complete system of equations (equations (2.1)–(2.4) and *I*-times equation (2.7)) is solved at a given time *t* considering the ‘Lagrangian’ independent variable *m*, with *r*,*L*,*P*,*T* as unknowns, once the stellar mass and initial chemical composition are specified, and prescriptions for the equation of state of the stellar gas, Rosseland mean opacities, nuclear reaction cross section and energy generation rates, and neutrino production rates are provided. The chemical composition is usually denoted by *X*, *Y* , *Z*, that correspond to the mass fractions of H, He and all other elements collectively called ‘metals’, respectively. A relative distribution of the metal abundances also needs to be specified.

Notice that the chemical abundance profile enters explicitly equation (2.7) and *ϵ*_n_, and affects the coefficients *ϵ*_*ν*_, *ϵ*_g_, the opacities *κ* and the equation of state, which all depend on the chemical composition of the stellar matter.

**Figure 1.**
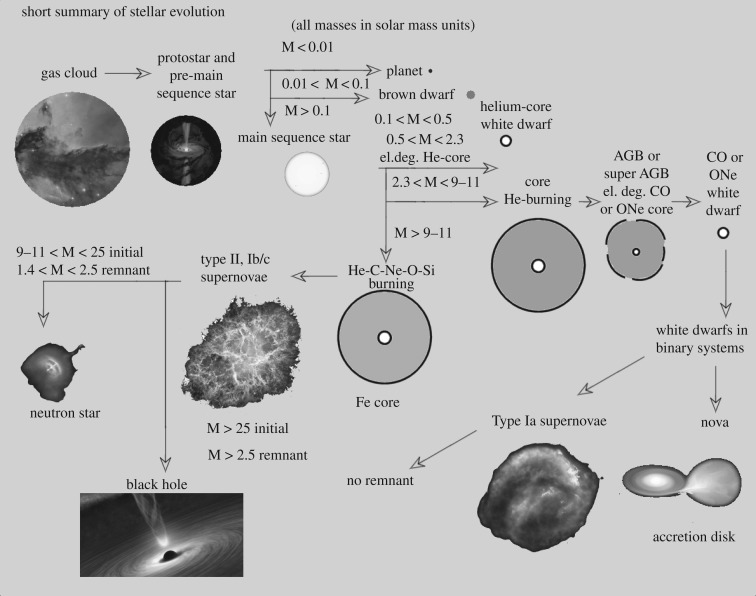
General evolutionary paths of single stars with different initial masses. The exact values of the mass ranges depend on the initial chemical composition and the details of the adopted element transport mechanisms. We show also some final product of the evolution of interacting binaries (Novae, Type Ia supernovae). He-core WDs from single stars can form only on timescales much longer than a Hubble time, but are produced nowadays by interacting binary systems. There are observational indications that massive stars with initial mass above approximately 20*M*_⊙_ might not explode as supernovae, rather collapse directly to a black hole [6].

Figure 1 shows an overview of the evolutionary paths of single stars with different initial masses (plus the main by-products of interacting binary evolution), as derived from complete stellar evolution models. This general evolutionary framework is solid and does not depend on the details of element transport modelling although the precise values of the various mass ranges do (and they depend also on the initial chemical composition). It constitutes a reference guideline for the discussions presented in the following sections.

It is clear that chemical element transport does not arise naturally from the equations of stellar structure and evolution. This makes it necessary to first identify all possible mechanisms—in addition to the nuclear reactions—able to change the chemical abundances at a given mass layer, and then to develop formalisms for their implementation in the 1D equations (a major difficulty). The element transport mechanisms described below will add extra terms to the right-hand side of equation (2.7), in addition to the terms describing the effect of nuclear reactions.

## 3. Convection

Besides radiation and electron conduction, convection is the third fundamental mechanism for energy transport in stars. It involves organized large-scale motions of matter that in addition to carrying energy are also a very efficient source of mixing. It can be envisaged as a flux of matter from deeper—hence hotter—stellar layers moving vertically outwards into cooler layers, and material from cooler outer layers flowing down to hotter inner layers.

The implementation of this element (and energy) transport in stellar models requires first a criterion for the onset of the convective instability, and then a mathematical treatment to predict the main physical properties of convective regions.

### 3.1. Instabilities in non-rotating stars

Matter inside the stars is never at rest, but usually the gas is locally subject to small random perturbations around equilibrium positions. Under certain conditions, these small random perturbations can trigger large-scale motions that involve sizable fractions of the total stellar mass. These large-scale motions are called convection, and are the equivalent to the motion of water elements in a kettle heated from below.

The treatment of convection in stellar interiors is extremely complicated and requires the introduction of various approximations. This stems from the fact that the flow of gas in a stellar convective region is highly turbulent, forcing us to adopt simplified models that provide only mean approximate values for the properties of the flow of gas. The so-called *mixing length theory* (MLT) is the local, time-independent convection model almost universally used in stellar evolution calculations, which we will discuss below. Firstly, we show how a simplified linear analysis is sufficient to determine the main criteria for the onset of mixing (not only convective) in stellar interiors [7].

**Figure 2.**
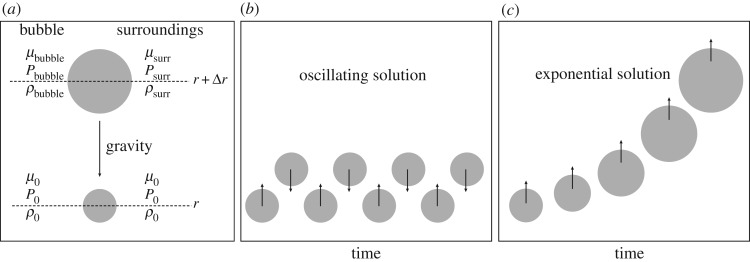
(*a*) Set-up of the simple analysis for the onset of instabilities (see text for details). (*b*) Stable situation. A blob of gas displaced vertically by a small amount oscillates around its equilibrium position. (*c*) Unstable situation. After the initial displacement, the blob of gas continues to rise as time goes on.

We consider a gas element at rest at a distance *r* from the star centre. This gas bubble will have a pressure *P*_0_, temperature *T*_0_, density *ρ*_0_ and mean molecular weight *μ*_0_ (the molecular weight is the mean mass of the gas particles in atomic mass units) equal to those of the environment, supposed to be in radiative equilibrium (in this section ‘radiative’ actually means ‘radiative plus conductive’) as shown in figure 2. If random motions displace the bubble by a small amount Δ*r* away from the equilibrium position, the equation of motion for an element of unit volume can be written as (assuming the viscosity is negligible)

$$\rho \frac{d^{2}\Delta r}{dt^{2}}=-g\Delta \rho ,$$

where Δ*ρ* is the difference (*ρ*_bubble_−*ρ*_surr_) between the bubble (supposed to have constant density) and the surroundings, and *g* is the local acceleration of gravity. One reasonable assumption is that the motion of the bubble is fast enough that all time derivatives of the mean stellar properties are equal to zero.

As a consequence of the displacement from its equilibrium position, two distinct physical situations can occur, as shown in figure 2. If the region is convectively stable, the displaced gas parcel experiences a restoring force that moves it back towards the original position, as illustrated in figure 2*b*. Indeed, the blob is subject to a stable oscillation around an equilibrium position with a frequency named the Brunt–Väisälä frequency (see below). If the bubble of material at position *r*+Δ*r* is less dense than the surrounding material, it will continue to be pushed upwards by buoyancy forces and the region is then said to be *convectively unstable*, as shown in figure 2*c*. Similarly, if the bubble displaced at a radial distance *r*−Δ*r* is denser than the surrounding material, its motion will continue because it is heavier than the local environment.

To determine which solution applies to a given layer within a star, we assume that along the displacement Δ*r* the bubble is always in pressure equilibrium with the surroundings, that is, Δ*P*=(*P*_bubble_−*P*_surr_)=0, and that the molecular weight of the bubble *μ*_bubble_ is always equal to its initial value *μ*_0_ (there is no matter exchange with the surroundings, hence the gas parcel retains its identity). The assumption of pressure equilibrium means that the motion of the bubble has to happen with a speed lower than the local sound speed. When there is a molecular weight gradient d*μ*/d*ρ* throughout the region, the difference Δ*μ*=(*μ*_bubble_−*μ*_surr_) will be equal to Δ*μ*=*μ*_0_−[*μ*_0_+(d*μ*/d*r*)Δ*r*], hence Δ*μ*=−(d*μ*/d*r*)Δ*r*.

Using the relationships d ln⁡(P)=(1/P) dP and d ln⁡(μ)=(1/μ) dμ, one gets

$$\Delta \mu =-\mu \left(\frac{d\;\ln(\mu )}{d\;\ln(P)}\right)\left(\frac{d\;\ln(P)}{dr}\right)\Delta r.$$

Differentiating with respect to time, one obtains

$$\frac{d\Delta \mu }{dt}=-\mu \frac{d\;\ln(\mu )}{d\;\ln(P)}\frac{d\;\ln(P)}{dr}\frac{d\Delta r}{dt}.$$

The temperature difference Δ*T*=(*T*_bubble_−*T*_surr_) depends on the difference between the temperature gradients in the bubble and in the surroundings and the rate of temperature change due to energy losses from the bubble (due, for example, to thermal diffusion) whose efficiency will depend on a parameter we denote as *ζ*, hence

$$\Delta T=\left[{\left(\frac{dT}{dr}\right)}_{\mathrm{ad}}-{\left(\frac{dT}{dr}\right)}_{\mathrm{rad}}\right]\Delta r-\zeta \Delta T\;dt.$$

By introducing the notation ∇≡(d ln⁡(T)/d ln⁡(P)) and differentiating with respect to time, one obtains

$$\frac{d\Delta T}{dt}=T\frac{d\;\ln(P)}{dr}(\nabla_{\mathrm{ad}}-\nabla_{\mathrm{rad}})\frac{d\Delta r}{dt}-\zeta \Delta T.$$

If Δ*P*=0, and in the assumption that the differences Δ*T*, Δ*ρ* and Δ*μ* are small, we obtain from the equation of state

$$\chi_{\rho }\frac{\Delta \rho }{\rho }+\chi_{T}\frac{\Delta T}{T}+\chi_{\mu }\frac{\Delta \mu }{\mu }=0,$$

where

$$\chi_{\rho }={\left(\frac{d\;\ln(P)}{d\;\ln(\rho )}\right)}_{T,\mu },\;\chi_{T}={\left(\frac{d\;\ln(P)}{d\;\ln(T)}\right)}_{\rho ,\mu }\;\mathrm{and}\;\chi_{\mu }={\left(\frac{d\;\ln(P)}{d\;\ln(\mu )}\right)}_{\rho ,T}.$$

We have derived in this way the following set of four homogeneous equations for the four unknowns Δ*T*, Δ*ρ*, Δ*μ* and Δ*r*:

3.1 $$\begin{array}{r} \rho \frac{d^{2}\Delta r}{dt^{2}}+g\Delta \rho =0, \end{array}$$

3.2 $$\begin{array}{r} \frac{d\Delta \mu }{dt}+\mu \frac{d\;\ln(\mu )}{d\;\ln(P)}\frac{d\;\ln(P)}{dr}\frac{d\Delta r}{dt}=0, \end{array}$$

3.3 $$\begin{array}{r} \frac{d\Delta T}{dt}+T\frac{d\;\ln(P)}{dr}(\nabla_{\mathrm{rad}}-\nabla_{\mathrm{ad}})\frac{d\Delta r}{dt}+\zeta \Delta T=0 \end{array}$$

3.4 $$\begin{array}{r} \;\text{and}\;\chi_{\rho }\frac{\Delta \rho }{\rho }+\chi_{T}\frac{\Delta T}{T}+\chi_{\mu }\frac{\Delta \mu }{\mu }=0. \end{array}$$

One can search for solutions of the form Δ*x*=*Ae*^*nt*^. By inserting into the respective equations this functional dependence for Δ*T*, Δ*ρ*, Δ*μ* and Δ*r*, a non-trivial solution is found when the determinant derived from the coefficients of *A*_*T*_, *A*_*ρ*_, *A*_*μ*_ and *A*_*r*_ is equal to zero, giving

3.5 $$n^{3}+\zeta n^{2}+\left[g\frac{\chi_{T}}{\chi_{\rho }}\frac{d\;\ln(P)}{dr}\left(\nabla_{\mathrm{rad}}-\nabla_{\mathrm{ad}}+\frac{\chi_{\mu }}{\chi_{T}}\nabla_{\mu }\right)\right]n+\left(\zeta g\frac{\chi_{\mu }}{\chi_{\rho }}\frac{d\;\ln(P)}{dr}\nabla_{\mu }\right)=0,$$

where we have defined $\nabla_{\mu }\equiv d\;\ln(\mu )/d\;\ln(P)$.

The condition that at least one of the *n* is real and positive or imaginary with a positive real part (unstable solutions) is given by the Hurwitz criterion, resulting in at least one of the following conditions to be satisfied (we recall that the pressure *P* always increases towards the star centre, *χ*_*μ*_ is negative, *χ*_*T*_ and *χ*_*ρ*_ positive):

3.6 $$\begin{array}{r} \Delta_{\mu }<0, \end{array}$$

3.7 $$\begin{array}{r} \nabla_{\mathrm{rad}}>\nabla_{\mathrm{ad}}-\frac{\chi_{\mu }}{\chi_{T}}\nabla_{\mu }\equiv \nabla_{L} \end{array}$$

3.8 $$\begin{array}{r} \;\text{and}\;\nabla_{\mathrm{rad}}>\nabla_{\mathrm{ad}}. \end{array}$$

If Δ_*μ*_<0 (*μ* increasing towards the surface), the medium is always unstable. When the gas parcel is displaced upwards (downwards) by a small distance Δ*r*, its density will be lower (higher) than the environment, and will continue to be pushed upwards (downwards) by buoyancy. The temperature difference between the displaced mass element and its surroundings suppresses or favours this displacement, depending upon the difference between ∇_rad_ and ∇_ad_ (more below). For the more common case of Δ_*μ*_≥0 (heavier elements are usually synthesized by nuclear reactions in the central regions), we should consider equation (3.7)—the so-called ‘Ledoux criterion’—and equation (3.8)—the so-called ‘Schwarzschild criterion’. The second term on the right-hand side of equation (3.7) is positive for a positive ∇_*μ*_ (the composition gradient has a stabilizing effect) so that if the gradients satisfy the Ledoux criterion, the Schwarzschild criterion is automatically satisfied, hence the Schwarzschild criterion determines the presence of an unstable medium.

These instability criteria are ‘local’, in the sense that they can be applied layer-by-layer without accounting for non-local effects that can be, however, relevant when dealing with convective mixing.

**Figure 3.**
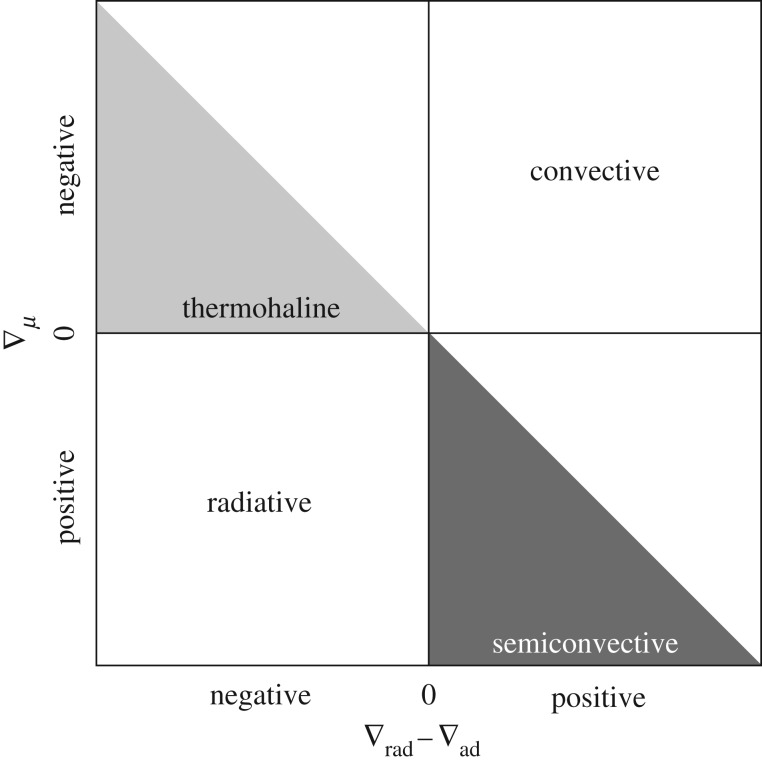
Sketch of the ∇_*μ*_−(∇_rad_−∇_ad_) stability plane with different regimes labelled (see text for details). The diagonal line dividing into half the top left and bottom right diagrams denotes ∇_L_.

Figure 3 displays a qualitative sketch in the ∇_*μ*_−(∇_rad_−∇_ad_) diagram of the region where instabilities occur, divided into four quadrants. The only stable region in this diagram is the bottom left quadrant, where ∇_*μ*_≥0 and ∇_rad_<∇_ad_. All other quadrants are unstable regions, although the type of mixing involved depends on the exact values of the gradients. If ∇_*μ*_≥0 and ∇_ad_<∇_rad_<∇_L_ (lower right quadrant), the instability is called ‘semiconvection’.

The linear analysis in the semiconvective regime shows that if the gas bubble is displaced upwards and *ζ*=0, its internal temperature will be slightly larger compared to the surroundings, hence its lower density will favour a continuation of the displacement. On the other hand, *μ* of the raising bubble will be larger than the environment and in the semiconvective regime of the gradients this effect will prevail, causing the return of the mass element to its starting position. When *ζ*>0, the temperature of the element returning from above is smaller than its initial value at *r*, therefore the restoring force is larger upon returning than when the bubble was leaving its starting position. As a consequence, the bubble returns back to a radial location *r* with a larger velocity than when it left for the upwards motion. On the downward excursion, the same effect (with an opposite sign) will operate and the amplitude of these oscillations will progressively increase due to the increasing radiative losses during the oscillatory motion.

The growth of these oscillations (overstability) will lead to chemical mixing and thus decrease or destroy the stabilizing gradient ∇_*μ*_. Results from numerical simulations and laboratory experiments show a more complex picture [8,9], with in some cases, the formation of well-mixed fully convective layers, separated by stratified interfaces, but the linear analysis suffices to highlight the peculiarity of the mixing associated with semiconvection.

The efficiency (timescale) of semiconvective mixing is difficult to estimate and depends on the efficiency of the bubble energy losses; an analysis of the growth rate in the framework of the linear analysis shows that as a result of semiconvective mixing ∇_rad_∼∇_ad_ [10], and this is another approach to treat semiconvective mixing in stellar evolution calculations, i.e. to impose a mixing efficiency such that ∇_rad_∼∇_ad_ (actually this was the original way in which semiconvection was implemented [11]) in a semiconvective region (see §4).

If ∇_*μ*_<0 and ∇_rad_<∇_L_ (top left quadrant), the instability is usually called a ‘thermohaline’ instability. If *ζ*=0, a gas bubble displaced downwards will have a larger *μ* than the environment, but also a larger temperature than the radiative environment. The combined effect is that the bubble density is lower than that of the environment, and buoyancy will push back the displaced gas element. However, if *ζ*>0, the energy loss will eventually decrease the bubble temperature enough to induce a further displacement downwards due to the effect of the larger *μ*.

This instability is controlled by the heat leakage of the displaced element, and it is observed, for example, when a layer of hot salt water lies over a layer of cold fresh water. Upon displacing downwards a blob of hot salt water, due to the fact that heat transfer by molecular collisions is typically faster than the motion of chlorine and sodium ions that cause the composition to equilibrate, the sinking blob will be able to come into thermal equilibrium with the surrounding medium faster than achieving composition equilibrium. Given that salt water is heavier than fresh water at the same temperature, the blob will continue to sink in the surrounding fresh water. As this motion continues, the medium develops ‘fingers’ of salt water reaching down into the fresh water. Inside a star, the role of salt can be played by a heavier element like helium, in a hydrogen-rich medium. This is an example of so-called ‘doubly diffusive instabilities’, because it involves the diffusion of two different components (particles and heat).

In general, a convectively unstable region mixes matter on very short timescales compared with evolutionary timescales (either nuclear or Kelvin–Helmholtz timescales), and the chemical profile in convective layers can always be assumed uniform to a very good approximation (instantaneous mixing approximation), with abundances of individual elements equal to values averaged over the whole convective region. If the convective region extends from mass layer *m*_1_ (inner boundary) to mass layer *m*_2_ (outer boundary) within the star, inside this region the abundance *X*_*s*_ of a generic element *s* is constant. At the boundaries (one or both of them), one may have a discontinuity between the homogeneous convective chemical profile and the profile in the radiative regions, for example, due to nuclear reactions (previous and/or present). Owing to these effects and just to give an example, the time evolution of *X*_*s*_ (in the approximation of instantaneous mixing) within an expanding convective shell is to a first order given by

3.9 $$\frac{dX_{s}}{dt}=\frac{1}{\Delta m}\left[\int_{m_{1}}^{m_{2}}\frac{dX_{s}}{dt}\;dm+\frac{dm_{2}}{dt}(X_{s2}-X_{s})-\frac{dm_{1}}{dt}(X_{s1}-X_{s})\right],$$

where Δ*m*=*m*_2_−*m*_1_, and *X*_*s*1_, *X*_*s*2_ are the abundances on the radiative side of the discontinuities at, respectively, the inner and outer boundary of the convective region. The first term in the integral describes the variation due to the nuclear burnings (if efficient), whereas the other two terms describe the change in composition when the boundaries of the convective zone move into surrounding regions of—in principle—inhomogeneous composition.

Exceptions to the validity of the instantaneous mixing in convective regions are the advanced evolutionary phases of massive stars about to explode as Type II supernovae (figure 1), the production of Li due to the Cameron–Fowler mechanism in the envelopes of red giant branch (RGB) stars [12] and proton ingestion episodes into the intershell convection zone of low-metallicity asymptotic giant branch (AGB) stars [13,14]. In this case, the nuclear-burning timescales in the convective regions are comparable to convective mixing timescales. This case is usually treated with a time-dependent convective mixing discussed in the next subsection.

Before closing this section, we just introduce a quantity that will appear often in the rest of the paper. In the case of *ζ*=0, gas elements in a stable region will oscillate around their equilibrium position with a frequency called the Brunt–Väisälä frequency, usually denoted with *N*. This can be derived easily from equation (3.5), and is equal to

3.10 $$N^{2}=-g\frac{d\;\ln P}{dr}\frac{\chi_{T}}{\chi_{\rho }}\left(\nabla_{\mathrm{rad}}-\nabla_{\mathrm{ad}}+\frac{\chi_{\mu }}{\chi_{T}}\nabla_{\mu }\right).$$

Another expression for this frequency (that we will use in the rest of the paper) involves the derivatives $\delta =-{\left(\partial \ln\rho /\partial \ln T\right)}_{P,\mu }$ and $\phi ={\left(\partial \ln\rho /\partial \ln\mu \right)}_{P,T}$, and the pressure scale height $H_{P}\equiv -dr/d\;\ln(P)=P/(\rho g)$ (where *g* is the gravitational acceleration), and is

3.11 $$N^{2}=N_{T}^{2}+N_{\mu }^{2}=\left(\frac{g\delta }{H_{P}}(\nabla_{\mathrm{ad}}-\nabla )+\frac{g\phi }{H_{P}}\nabla_{\mu }\right).$$

Using *δ* and *ϕ*, the gradient ∇_L_ (Ledoux gradient) can be rewritten as

3.12 $$\nabla_{L}\equiv (\nabla_{\mathrm{ad}}+\frac{\phi }{\delta }\nabla_{\mu }).$$

### 3.2. The mixing length theory of convection

From the point of view of the evolution of chemical abundances, the instantaneous mixing approximation does not require a model for stellar convection. However, this is necessary for calculating convective velocities and fluxes, and when a time-dependent description of convective mixing is required. The formalism almost universally used in stellar evolution calculations is the MLT [15], a simple, local, time-independent model, firstly applied to stellar modelling by Biermann [16]. The formulation by Böhm-Vitense [17] is usually employed in modern stellar evolution calculations.

**Figure 4.**
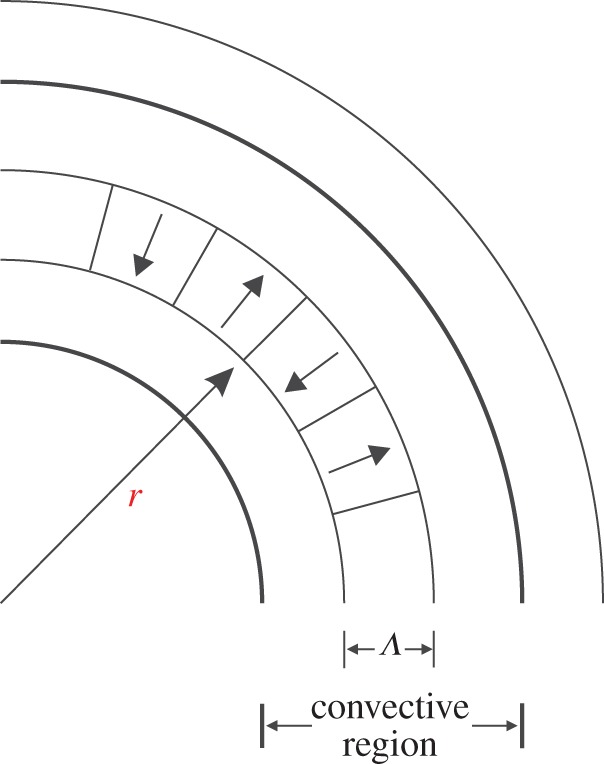
Schematic illustration of the MLT approximation to convective motion. The mixing length *Λ* corresponds to the characteristic radial distance scale over which rising and falling convective elements move before merging with the surrounding medium.

The basic idea of the MLT is to assume that the stellar fluid is composed of identifiable convective elements that move vertically in the gravitational field between regions of higher and lower temperature. Indeed, there is no net mass flow, but the effect is an outward transport of energy. The MLT assumes a characteristic distance over which bubbles rise before dissipating, the so-called mixing length *Λ* (figure 4), and describes the motion of these bubbles over the characteristic scale *Λ* under some general assumptions:

— all bubbles have the same characteristic size that is of the same order as *Λ*;
— *Λ* is much smaller than any other length scale of physical significance in the star;
— the physical properties, i.e. temperature, density, pressure and chemical composition, of the bubbles differ only slightly from the surrounding medium;
— pressure equilibrium with the environment is maintained. This means that the velocities of the convective elements are small compared with the local sound speed in the local environment.

*Λ* is assumed to be equal to a multiple of the local pressure scale height *H*_*p*_, i.e. *Λ*=*α*_MLT_*H*_*P*_, with *α*_MLT_—the *mixing length parameter*—being a constant to be empirically calibrated (usually by reproducing the solar effective temperature at the solar age with a solar model).

The need for a mean free path in the simple framework of the MLT can be easily explained as follows. Let us consider the cross sections of the rising and falling gas columns; if originally in a given convective layer the cross sections were the same, the rising gas (always in pressure equilibrium with the surroundings) will expand by a factor *e* after a distance equal to *H*_*P*_. This means that at this point within the star there is much less space available for the falling gas. On the other hand, the amount of falling material must be the same as the rising one, otherwise the star would either dissolve or concentrate all its mass in the interior, thus violating the hydrostatic equilibrium condition. The only solution is that after a distance *Λ* of the order of *H*_*P*_, part of the material stops and inverts its motion.

By relying on the previous assumptions, the MLT provides the following equations for the velocity of the convective elements *v*_c_, the convective flux *F*_c_ and the convective efficiency *Γ* (defined as the ratio between the excess heat content of a raising convective bubble just before its dissolution, and the energy radiated during its lifetime) [18]:

3.13 $$\begin{array}{r} v_{c}^{2}=\frac{a\Lambda^{2}g\delta (\nabla_{c}-\nabla^{\prime })}{H_{p}}, \end{array}$$

3.14 $$\begin{array}{r} F_{c}=\frac{b\rho v_{c}c_{p}T\Lambda (\nabla_{c}-\nabla^{\prime })}{H_{p}} \end{array}$$

3.15 $$\begin{array}{r} \;\text{and}\;\Gamma \equiv \frac{\nabla_{c}-\nabla^{\prime }}{\nabla^{\prime }-\nabla_{\mathrm{ad}}}=\frac{4c_{p}\rho^{2}\Lambda v_{c}\kappa }{c^{2}aT^{3}}, \end{array}$$

where ∇^′^ is the temperature gradient of a rising (or falling) element of matter within the convective region, and ∇_c_ is the average temperature gradient of all the matter at a given level within the convective zone (the quantity needed to solve the stellar structure equations). There are three additional free parameters besides *α*_MLT_, i.e. *a*, *b* and *c*, that are usually fixed *a priori* and define the MLT ‘flavour’ [19]^5^ There are four unknowns, namely *v*_c_, *F*_c_, ∇_c_, ∇^′^ and the three previous equations plus an additional equation arising from the fact that the total flux to be transported by radiation plus convection is known from the solution of the stellar evolution equation

3.16 $$\frac{L}{4\pi r^{2}}=\frac{4acgT^{4}m}{3\kappa Pr^{2}}\nabla +F_{c}.$$

If convection is efficient in the deep stellar interiors, the MLT provides $\nabla \to \nabla_{\mathrm{ad}}$ and velocities of the order of 1–100 m s^−1^, many orders of magnitude smaller than the local sound speed. On the contrary, in convective layers close to the surface the gradient is strongly superadiabatic and velocities are much larger, of the order of 1–10 km s^−1^, close to the local sound speed.

External or inner regions of a stellar model are convective in the following cases:

— Large values of the opacity *κ*. Given that ∇_rad_∝*κF*, where *F* is the energy flux, ∇_rad_ tends to increase above ∇_ad_. The radiative opacity generally increases with decreasing temperature—for a given chemical composition—hence this situation occurs most commonly in the cooler outer layers of stars.
— If the energy generation rate in the star is very sensitive to the temperature, then the energy flux *F* rises rapidly as *r* approaches zero in the stellar centre. This large heat flow can eventually cause ∇_rad_ to increase above ∇_ad_. This situation occurs only in stellar cores, when the nuclear energy generation rate is very sensitive to temperature, as is the case of the H-burning CNO-cycle, He-burning and more advanced nuclear-burning stages.
— In ionization zones the adiabatic gradient can decrease below the typical value ∇_ad_∼0.4. Also, in these regions the radiative opacity usually increases. Owing to these effects, one can expect the ionization zones located in the outer layers to be convective.

When the convective mixing timescales are comparable to nuclear-burning timescales, a diffusive approach is usually employed to follow the chemical evolution [20]. This means that an extra term is added to the right-hand side of equation (2.7) for a given element *s*, i.e. the right-hand side of the following diffusion equation:

3.17 $${\frac{\partial X_{s}}{\partial t}|}_{M_{r}}=\frac{1}{\rho r^{2}}\frac{\partial }{\partial r}\left(D_{c}\;\rho r^{2}\frac{\partial X_{s}}{\partial r}\right),$$

where the diffusion coefficient associated with the convective transport is taken to be $D_{c}=\frac{1}{3}\alpha_{\mathrm{MLT}}v_{c}H_{P}$, using the value of *v*_c_ derived from the MLT (see [21–23], for examples).

The MLT is very appealing for its mathematical simplicity (despite the free parameters involved) and the fact that it relies just on local quantities, hence it is easy to include in stellar evolution codes and does not affect the stability of the numerical solution of the stellar evolution equations. Once *α*_MLT_ is calibrated on the Sun, the resulting HRDs (and CMDs) of the models generally reproduce the observations satisfactorily within the current errors.^6^ Comparisons of the effective temperature evolution of models calculated with a solar *α*_MLT_ and with a calibration of *α*_MLT_ obtained from 3D radiation hydrodynamics simulations of stellar envelopes [25] display an agreement within 30–50 K [26].^7^ On the other hand, helioseismic data (the study of non-radial oscillations of the Sun [28]) clearly point to shortcomings of the MLT description of the physical structure of convective regions [29].

An alternative local description of convection included in stellar evolution calculations with the ATON code [30] is presented in [31]. The main difference from the MLT is that the convective elements have a spectrum of sizes, and the scale length of the convective motions is set to be equal to the distance to the closest convective boundary. Stellar evolution tracks calculated with this convection model show a different evolution of the effective temperature *T*_eff_ compared to calculations with the MLT, especially along the RGB phase. Differences increase with increasing initial metallicity. This convection model also suffers from shortcomings when compared with helioseismic results [29].

There are very sophisticated non-local Reynolds stress models that describe not only convection, but also (see below) semiconvection and overshooting [32–37]. They introduce a large number of equations to be coupled to the stellar structure equations and several free parameters to be calibrated observationally, and are generally not included in current stellar evolution modelling.

**Figure 5.**
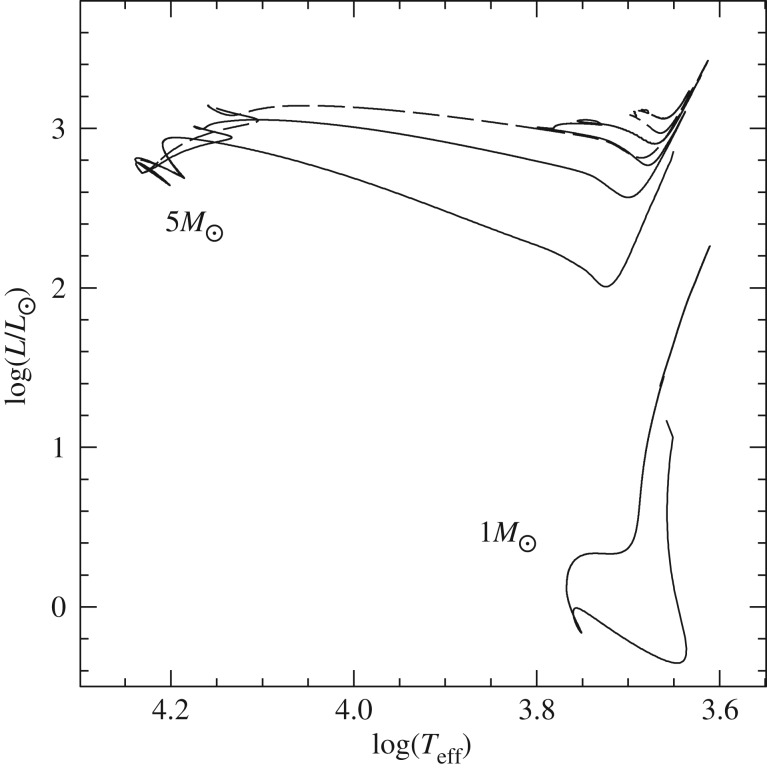
HRD of 1*M*_⊙_ and 5*M*_⊙_ stellar evolution models with the initial solar chemical composition. The 5*M*_⊙_ model is computed until the end of He-core burning. The 1*M*_⊙_ model stops during the RGB evolution. The dashed line displays the HRD of the same 5*M*_⊙_ model calculated with MS core overshooting (0.2*H*_*p*_—see text for details).

**Figure 6.**
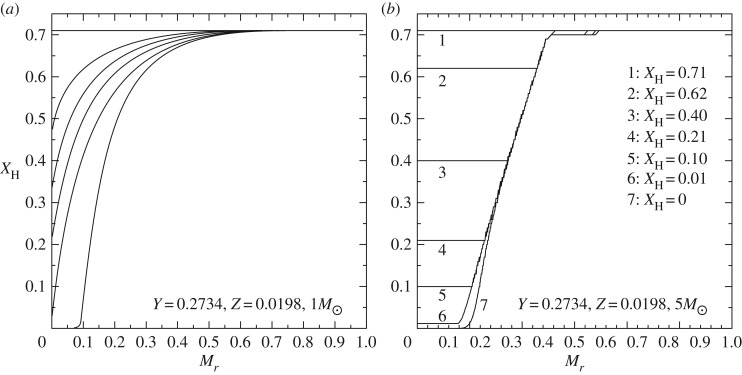
(*a*) The evolution with time of the H-abundance profile (H abundance as a function of the local fractional mass) within the 1*M*_⊙_ model of figure 5, during the MS phase. (*b*) The same, but for the 5*M*_⊙_ model without core overshooting. The different numbers label a temporal sequence with decreasing values of the H abundance in the mixed core.

Convection plays a major role in determining the evolutionary properties of stellar models. Figure 5 shows the HRD of models with (the 5*M*_⊙_ model) and without (the 1*M*_⊙_ model) convective cores during the MS. The shape of this evolutionary phase in the HRD is completely different between the two tracks, because of the different time evolutions of the H-profile in the burning region, as shown by figure 6. In the inner radiative layers of the 1*M*_⊙_ model, the H-abundance profile changes smoothly during the MS evolution, and at any given time the H abundance increases gradually from the core towards the more external layers where the burning becomes progressively less efficient. In the convective inner layers of the 5*M*_⊙_ model—where the burning takes place—the H-abundance profile is uniform, and the progressive retreat with time of the outer border of the convective core produces at a given time *t* a H-abundance profile flat in the innermost regions, then increasing outwards.

Surface convection (present everywhere along the HRD evolution, apart from the MS phase of the 5*M*_⊙_ model) is very important when comparing the surface abundances measured from spectroscopy with the models, because of the dredge-up phenomenon, whereby the fully mixed convective envelopes reach layers processed by nuclear burnings, hence altering their chemical composition during the RGB and AGB phases [2].

### 3.3. Convective overshooting

As already mentioned, the criteria for the onset of convection are local. The boundaries of the convective regions are fixed at the layer where the random motions of the gas do not get amplified. At this convective border, the acceleration of the gas elements is zero, but not the velocity. One expects, therefore, the chemically mixed region to extend into the formally stable layers in the case of both core and envelope convection. In real stars mixing beyond the formal boundary most probably results from the interplay of several physical processes [38–40], grouped in stellar evolution modelling under the ‘umbrella’ term *overshooting* (or sometimes *convective boundary mixing*). These overshooting processes are modelled very crudely by introducing free parameters to be calibrated on observations of eclipsing binaries [41,42] (typically comparisons with masses and radii of both components under the assumptions that they are coeval) and open clusters [43] (the shape of the MS turn-off (TO) region).

The standard approach is to assume that overshooting into the stable layers does not affect their thermal structure, hence the temperature gradient stays radiative. The composition is then mixed instantaneously between the formal convective border and layers at a distance λ*H*_*P*_ from this border, where λ (λ<1) is a free parameter to be observationally calibrated, and *H*_*P*_ is the pressure scale height at the convective border. This is the approach taken, for example, in the BaSTI [44], DSEP [45], Yale–Yonsei [46] codes.

A similar approach of instantaneous mixing is taken in [47]. The following integral criterion is employed to fix the extent of the convective core overshooting, inspired by a constraint on the maximum possible extension of the overshooting region developed by Roxburgh [48]

3.18 $$\int_{0}^{r_{\mathrm{cc}}}F_{\mathrm{over}}(L_{\mathrm{rad}}-L_{N})\frac{1}{T^{2}}\frac{dT}{dr}\;dr+\int_{r_{\mathrm{cc}}}^{r_{\mathrm{ov}}}(2-F_{\mathrm{over}})(L_{\mathrm{rad}}-L_{N})\frac{1}{T^{2}}\frac{dT}{dr}\;dr=0,$$

where *F*_over_ is a free parameter (between 0 and 1) to be calibrated against observations. The two luminosities *L*_rad_=−((16*πacT*^3^*r*^2^)/(3*κρ*)) d*T*/d*r* and *L*_N_ represent, respectively, the local radiative luminosity and the total luminosity produced by nuclear reactions within radius *r*, while *r*_cc_ is the radius of the convective core boundary and *r*_ov_ is the radius of the outer boundary of the overshooting region, to be determined through this equation.

The various releases of Padua stellar evolution models [49] and the PARSEC code [50] consider instead an adiabatic gradient for the overshooting region (this is the case of *penetrative convection* according to [51], because the overshooting material is assumed to be able to change the entropy stratification), and the spatial size of the overshooting region is determined as follows [52]. Starting from each radial distance *r*_*i*_ from the centre inside the formally convective region, the following equation (based on the MLT) is integrated outwards, up to *r*=*r*_*i*_+*l*, where *l*=λ*H*_*p*_, *H*_*p*_ being the pressure scale height at the convective boundary, and λ (λ<1) a free parameter:

3.19 $$\frac{1}{3}\frac{\partial v_{r}^{3}}{\partial r}=\frac{g}{\kappa T}\frac{\chi_{T}}{\chi_{\rho }}\frac{F_{c}}{c_{P}\rho }-\frac{g}{\mu }\frac{\chi_{\mu }}{\chi_{\rho }}\Delta \mu v_{r},$$

with Δ*μ*=*μ*(*r*)−*μ*(*r*_*i*_). The convective flux *F*_c_ is determined as *F*_c_=*F*−*F*_rad_, where *F*=*L*/(4*πr*^2^) is the total energy flux and *F*_rad_=−((4*acT*^3^)/(3*κρ*)) d*T*/d*r*. In the overshooting region, where the actual gradient is set to adiabatic, *F*_rad_>*F* hence *F*_c_<0, mimicking the fact that the convective elements penetrating into the stable layers are formally cooler than the surrounding medium.

At any given *r*, the convective elements originated in the range between *r*−*l* and *r* will display a range of velocities, and the maximum value *v*_m_ attained at each *r* is taken to derive the run of *v*_m_(*r*) as a function of *r*. The border of the instantaneously mixed region is then taken at the radial distance for which *v*_m_=0.

In all these approaches, the free parameters that determine the spatial extent of the convective core overshooting region needs to decrease in the regime of small convective cores. The reason is that $H_{P}\to \infty$ as r→0, hence the smaller the size of the formally convective core, the larger the size of the actual mixed core (including overshooting region), with no smooth transition of the shape of MS evolutionary tracks from the convective core to the radiative core case. Observations of the MS TO of open clusters of various ages confirms the need to decrease to zero the extent of the overshooting regions when the stellar mass decreases, in the regime of small convective cores (masses below approx. 1.5*M*_⊙_—see, e.g. [47]). This means that the assumed trend of λ (or *F*_over_) with mass, for masses with small convective cores, introduces an additional degree of freedom, albeit affecting only a restricted range of stellar masses. In this context, following an integral constraint on the maximum possible extent of overshooting regions [48,53], it has been proposed to limit the extent of the overshooting region to 15% of the radius of the formally convective core to generate a smooth transition from models with convective cores to models with radiative cores on the MS [54].

Additionally, one can find in the literature two very different approaches to the mixing beyond the formal convective boundary.

(i) A diffusive approach [55] used, for example, in the STARS [56], MESA [57,58] and GARSTEC [59] codes. The element transport beyond the formal convective boundary is described as a diffusive process (avoiding the instantaneous mixing approximation) based on results of 2D radiation hydrodynamics simulations of shallow stellar surface convection zones [60]
  3.20 $${\frac{\partial X_{i}}{\partial t}|}_{M_{r}}=\frac{1}{\rho r^{2}}\frac{\partial }{\partial r}\left(D_{\mathrm{ov}}\;\rho r^{2}\frac{\partial X_{i}}{\partial r}\right).$$
  The diffusion coefficient *D*_ov_ is given by
  3.21 $$D_{\mathrm{ov}}=D_{c}\;\exp\;\left(-\frac{2z}{fH_{p}}\right),$$
  where *D*_c_ is the diffusion coefficient inside the convective region ($D_{c}=(\frac{1}{3})\alpha_{\mathrm{MLT}}v_{c}H_{P}$), *z* is the distance from the convective boundary, *H*_*P*_ is the pressure scale height at the convective boundary and *f* is a dimensionless free parameter. Typical calibrated values of *f* are approximately 0.01–0.02.
(ii) Modelling of the mixing as ‘turbulent entrainment’, following the simulations by Meakin & Arnett [61], as employed in the stellar evolution calculations by Staritsin [62]. The motion of matter in the zone of convective instability is turbulent, and the rising turbulent flow spreads horizontally near the boundary of the turbulent region. The interface between the convective region and the stable layers moves through the stable region due to the continuous involvement of new layers in the turbulent motion. The velocity *V*_e_ of the penetration of the convective turbulent boundary into the stable layers is determined by the ‘turbulent-entrainment’ law written as
  3.22 $$\frac{V_{e}}{V_{t}}=ARi_{B}^{-n},$$
  where *V*_t_ is a typical turbulent velocity at the boundary (that can be taken, for example, from the MLT), *A* and *n* are parameters that characterize the entrainment (*A*∼0.027 and *n*∼1.05 according to the simulations by Meakin & Arnett [61]), and *Ri*_B_ is the so-called bulk Richardson number defined as
  3.23 $$Ri_{B}=\frac{l\Delta b}{V_{t}^{2}},$$
  with *l* denoting the typical size of the eddies doing the entrainment (some fraction of the pressure scale height *H*_*p*_ at the boundary of the mixed region) and
  3.24 $$\Delta b=\int_{\Delta h}N^{2}\;dr.$$
  This integral is performed across a region of thickness Δ*h* that contains the convective boundary, *N*^2^ being the the Brunt–Väisälä frequency. When *V*_e_ is determined, the distance *d* over which this boundary shifts during an evolutionary time step Δ*t* is given by *d*=*V*_e_Δ*t*.

Despite the uncertainties involved in its parametrization, the treatment of overshooting is very important because it affects the evolutionary properties of the models. For example core overshooting during the MS produces brighter models, an increased MS lifetime (because more fuel is available), larger He-core masses that induce a brighter and shorter-lived He-burning phase, and also less extended loops in the HRD (figure 5). Overshooting below convective envelopes can alter the surface abundances after the dredge-up episodes [63], and affect the luminosity of the RGB bump (see §5) and also the extension of the loops in the HRD (in the case of the 5*M*_⊙_ model in figure 5, the loop during the core He-burning phase would become more extended with overshooting also from the convective envelope). Age estimates of young–intermediate age clusters are obviously affected by the amount of overshooting included in the models, i.e. the larger the overshooting region the older is the age estimate of a given cluster. Overshooting also plays an important role during C-burning in super AGB stars, affecting the propagation of the carbon-burning flame [64]. Too large overshooting at the base of the convective C-burning region can prevent the flame from reaching the centre, thus producing a hybrid CONe core, and eventually a CONe WD.

It has also been shown that the diffusive approach to mixing in the overshooting region provides different results in terms of evolutionary times compared to instantaneous mixing, because of a slower addition of extra fuel from the overshooting region when this scheme is implemented [23].

## 4. Semiconvection

After convection, semiconvection (called ‘double-diffusive convection’ in oceanography) is probably the most significant element transport mechanism in non-rotating stellar evolution models. Below are the two major cases where semiconvective transport is efficient, and how this is implemented in stellar models.^8^

### 4.1. H-burning phase with convective cores

**Figure 7.**
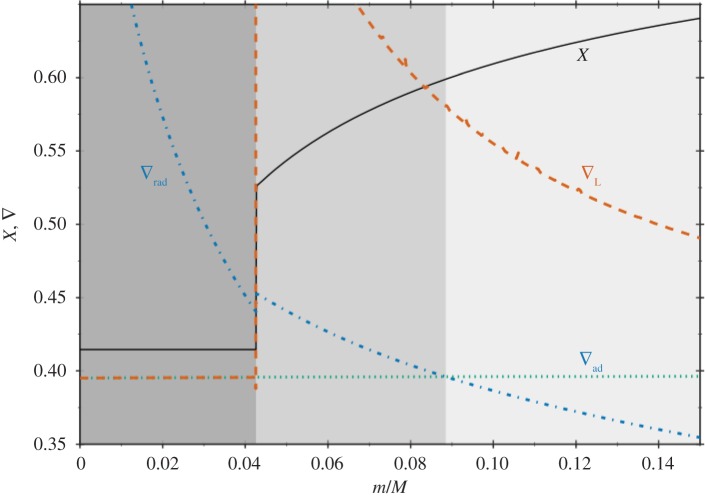
Profiles of the hydrogen mass fraction *X* and the radiative, adiabatic and Ledoux gradient as a function of the fractional mass coordinate, within a 1.5*M*_⊙_ model during the MS phase. From left to right, different tones of grey mark convective, semiconvective and radiative layers, respectively (courtesy of V. Silva-Aguirre).

There is a large body of literature that addresses the issue of semiconvection in massive stars with convective cores during the MS [10,11,66–68]. In a nutshell, layers left behind by shrinking convective cores during the MS will have a hydrogen abundance that increases with increasing radius (the chemical composition of each layer is determined by the composition of the convective core at the moment the layer has detached from the retreating mixed region). They are characterized by ∇_ad_<∇_rad_<∇_L_, and a treatment of semiconvective mixing is needed (figure 7). There is also a narrow range of masses around 1.5*M*_⊙_ (the values depending on the initial chemical composition) with increasing mass of the convective core during part of the MS, where a narrow semiconvective region forms right above the fully mixed core [69].

The efficiency of element transport in this semiconvective region has important consequences for the morphology of the TO of the tracks—very efficient semiconvection mimics the case of overshooting beyond the formal convective boundary and no semiconvection [69]—and the post-MS evolution of massive stars [70,71] because it changes the H-profile above the H-exhausted region. To provide a guideline, for massive stars inefficient mixing favours core He-ignition on the cool (red) side of the HRD, while efficient mixing favours the ignition on the hot (blue) side of the HRD (and increases MS evolutionary timescales). One could think that comparisons with the observed ratio of blue to red supergiants (B/R ratio) could provide strong constraints on the efficiency of semiconvective mixing in massive stars. However, other factors like the extent of the overshooting region (that acts in the direction to reduce the size of the semiconvective layers), and rotation will affect the HRD location (and eventually loops) during the He-burning phase [72]. At the moment no set of theoretical models seems to be able to match observational constraints on the B/R ratio, that is also strongly affected by mass transfer in binaries, which make up most of the massive star population.

Semiconvective mixing is included in these evolutionary calculations following various recipes. A traditional approach was to iterate the composition in the individual semiconvective layers until ∇_rad_=∇_ad_ locally [11,70,71]. More modern calculations adopt again a diffusive approach, including a chosen semiconvective diffusion coefficient *D*_SC_. The coefficient from [10] is implemented in the codes STERN [73], MESA and GARSTEC. It is derived from a linear local stability analysis [7], assuming the MLT to determine the velocity of the gas elements (using *α*_MLT_=1.5), and is given by

4.1 $$D_{\mathrm{SC}}=\frac{\alpha_{\mathrm{SC}}K}{6c_{p}\rho }\frac{\nabla -\nabla_{\mathrm{ad}}}{\nabla_{L}-\nabla },$$

where *K*=(4*acT*^3^)/(3*κρ*) is the thermal conductivity, ∇ is the actual temperature gradient and the free parameter *α*_SC_ determines the mixing timescale (larger *α*_SC_ correspond to shorter mixing timescales). Values of *α*_SC_ currently used are of the order of approximately 0.04–0.1, calibrated on empirical constraints [22,72]. The value of ∇ in the semiconvective region is determined from

4.2 $$\begin{array}{rl} L & =L_{\mathrm{rad}}\left(1+\left(\frac{L_{\mathrm{SC}}}{L_{\mathrm{rad}}}\right)\right)\\ \;\text{and}\;\;\frac{L_{\mathrm{SC}}}{L_{\mathrm{rad}}} & =\alpha_{\mathrm{SC}}\frac{\nabla -\nabla_{\mathrm{ad}}}{2\nabla (\nabla_{L}-\nabla )}\left[(\nabla -\nabla_{\mathrm{ad}})-\frac{\beta (8-3\beta )}{32-24\beta -\beta^{2}}\nabla_{\mu }\right], \end{array}\}$$

where *β* is the ratio of the gas pressure to the total pressure (gas plus radiation), *L* is the total luminosity and *L*_rad_ is the radiative luminosity, which can be written as

$$L_{\mathrm{rad}}=\frac{16}{3}\frac{\pi acGmT^{4}}{\kappa P}\nabla .$$

Larger values of *α*_SC_ produce semiconvective temperature gradients ∇ increasingly close to ∇_ad_.

Another expression for *D*_SC_ has been derived in the assumption of layering of the semiconvective region, with nearly uniform composition in each layer, separated by thin boundary layers within which the chemical elements are transported by molecular diffusion alone [74]:

4.3 $$D_{\mathrm{SC}}=\sqrt{D_{s}K_{T}}\left(\frac{4}{\beta }-3\right)\frac{\nabla_{\mathrm{rad}}-\nabla_{\mathrm{ad}}}{\nabla_{\mu }}.$$

where *K*_*T*_=*K*/(*ρc*_*P*_) is the thermal diffusivity, *β* is the ratio of the gas pressure to the total pressure (gas plus radiation) and *D*_*s*_ is the diffusion coefficient of He due just to the He abundance gradient (see §6). For a chemical composition of essentially two elements (in our case H and He) with atomic numbers *Z*_1_ and *Z*_2_, masses *m*_1_ and *m*_2_ and number densities *n*_1_ and *n*_2_,

$$D_{s}=\frac{3}{16n}{\left(\frac{2K_{B}T}{\pi m}\right)}^{1/2}{\left(\frac{2K_{B}T}{Z_{1}Z_{2}e^{2}}\right)}^{2}\frac{1}{\ln(\Lambda )},$$

where *m*=*m*_1_*m*_2_/(*m*_1_+*m*_2_), *n*=*n*_1_+*n*_2_, *K*_B_ is the Boltzmann constant and

$$\Lambda =1+{\left(\frac{4\lambda_{D}K_{B}T}{Z_{1}Z_{2}e^{2}}\right)}^{2},$$

with λ_D_=(*K*_B_*T*/(4*πn*_e_ *e*^2^))^1/2^ (Debye length) and *n*_e_ the electron density.

Typically, *D*_*s*_ is very small (smaller by about eight orders of magnitude) compared with *K*/(*c*_*p*_*ρ*) (the so-called thermal diffusivity) and the predicted semiconvective transport is very inefficient.

The code KEPLER [75,76] implements a different diffusion coefficient for the semiconvective transport:

4.4 $$D_{\mathrm{SC}}=\frac{\alpha_{\mathrm{SC}}K_{T}D_{c}^{\prime }}{D_{c}^{\prime }+\alpha_{\mathrm{SC}}K_{T}},$$

where $D_{c}^{\prime }$ is the diffusion coefficient the layer would have if the Schwarzschild criterion is used (fully efficient convection, $D_{c}^{\prime }=\frac{1}{3}\alpha_{\mathrm{MLT}}v_{c}H_{P}$) and *α*_SC_ is a free parameter usually fixed to 0.1 in KEPLER calculations [77].

Finally, results from recent 3D hydrodynamics simulations of layered semiconvective regions [9] have been transposed into a diffusion coefficient *D*_SC_ implemented in the MESA code. The result is that in stellar conditions the mixing obtained with this coefficient is very fast and is essentially equivalent to calculations performed with instantaneous mixing in the semiconvective region [78].

### 4.2. Core He-burning phase in low–intermediate-mass stars

**Figure 8.**
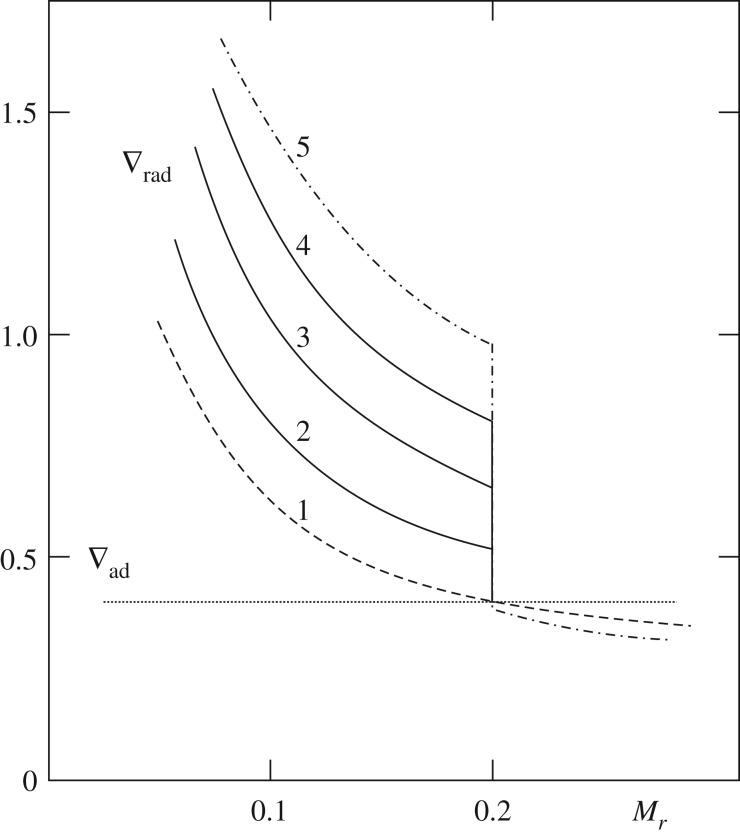
Time evolution of ∇_rad_ as a function of the mass enclosed within radius *r* inside a core He-burning model, if the discontinuity of ∇_rad_ is maintained. Increasing numbers define a sequence of increasing time (see text for details).

Another important evolutionary stage where semiconvection plays a major role is the core He-burning phase of low- and intermediate-mass stars, which has been widely discussed in the literature (see [79–82] and references therein). If we consider a low-mass star (initial mass below approx. 2*M*_⊙_) after the core He-flash, He-burning is efficient in a convective core with central values $\log(T_{c})∼8.07\pm 0.01$, $\log(\rho_{c})∼4.25\pm 0.05$, and a chemical composition of almost pure He. The opacity is dominated by electron scattering, but an important contribution (about 25% of the total) comes also from *free–free* absorption. The transformation of He to C due to nuclear burning increases the *free–free* opacity, hence *κ*, and within the convective core ∇_rad_ increases, developing a discontinuity of ∇_rad_ at the inner convective boundary (figure 8).

This discontinuity of the radiative gradient is clearly unphysical. In fact, in convective regions far from the surface ∇_c_∼∇_ad_, and given that in the MLT *F*_c_∼(∇_rad_−∇_c_), there would be an increasing convective flux developing on the inner side of the convective boundary. According to [83], the presence of this discontinuity is due to an incorrect application of the Schwarzschild criterion when an MLT picture of convection is considered, for a proper implementation requires always ∇_rad_=∇_ad_ at the convective side of the boundary. Another interpretation is that even a very small amount of overshooting suffices to reach the neutral ∇_rad_=∇_ad_ condition at the convective boundary, by means of a *self-driving* mechanism.

Let us consider an overshooting such that just one radiative layer is mixed. This layer will become fully convective on very short timescales, with locally ∇_rad_>∇_ad_ because of the amount of carbon mixed from the layers below. This means that it is now the formal boundary of a new enlarged convective core. A small overshooting to mix the next radiative layer will have the same effect, and so on until finally ∇_rad_=∇_ad_ at the convective side of the boundary. Another possibility is atomic diffusion [84] (see §6), driven by the gradient in carbon abundance between the fully mixed convective core and the surrounding radiative layers. As carbon diffuses into the radiative He-rich layers, the local opacity increases, ∇_rad_ becomes larger than ∇_ad_ and these layers become convective.

This diffusion is able to ‘extend’ the convective core on short timescales (short compared to nuclear timescales), so that ∇_rad_=∇_ad_ is satisfied at the convective boundary [84]. Yet another possibility to extend the formally convective core to attain ∇_rad_=∇_ad_ at the convective side of the boundary is the shear instability [85] (see also §8). At the formal boundary of the convective region (where the discontinuity of ∇_rad_ appears), we have two fluids of mean molecular weight *μ*_1_ and *μ*_2_, respectively, whose surface of separation is perpendicular to the gravity field, and have a relative velocity *v*_tang_ tangential to the separation surface. In this situation *v*_tang_∼*v*_c_, the velocity of the overturning convective elements when they reach the formal convective boundary, as obtained from the MLT.^9^ In this case, a mixed transition region should appear whose width is

4.5 $$z=\frac{v_{c}^{2}}{4g(1-\mu_{1}/\mu_{2})}.$$

By denoting with *t*_mix_ the typical time to mix this region of width *z*, and assuming that *t*_mix_ is of the order of the characteristic time of convection at the boundary of the core (as suggested by Castellani *et al*. [79]), i.e. *t*_mix_=*Λ*/*v*_c_, with *Λ*∼*H*_*P*_, the velocity of advancement of the border of the mixed core is *v*_*p*_≈*z*/*t*_mix_≈*zv*_c_/*Λ*. Combining these relations with equation (4.5) provides

4.6 $$v_{p}=\frac{v_{c}^{3}}{4g\Lambda (1-\mu_{1}/\mu_{2})}.$$

It has been shown in [79] using an MLT approach that an overshooting length *z*≈5 cm guarantees an advancement of the fully mixed core with speed $v_{p}\approx 10^{3}[1-(\nabla_{\mathrm{ad}}/\nabla_{\mathrm{rad}})]\;\mathrm{cm}\;{\mathrm{yr}}^{-1}$. This is sufficient to enlarge the mass of the fully mixed core to the point where ∇_ad_=∇_rad_) on timescales shorter than nuclear timescales. By adopting the values of *g*, *v*_c_, λ and *μ*_1_/*μ*_2_ adopted by Castellani *et al*. [79], equation (4.5) and (4.6) provide *z*∼3 cm, and *v*_*p*_≈10^3^ cm yr^−1^, consistent with results in [79].

**Figure 9.**
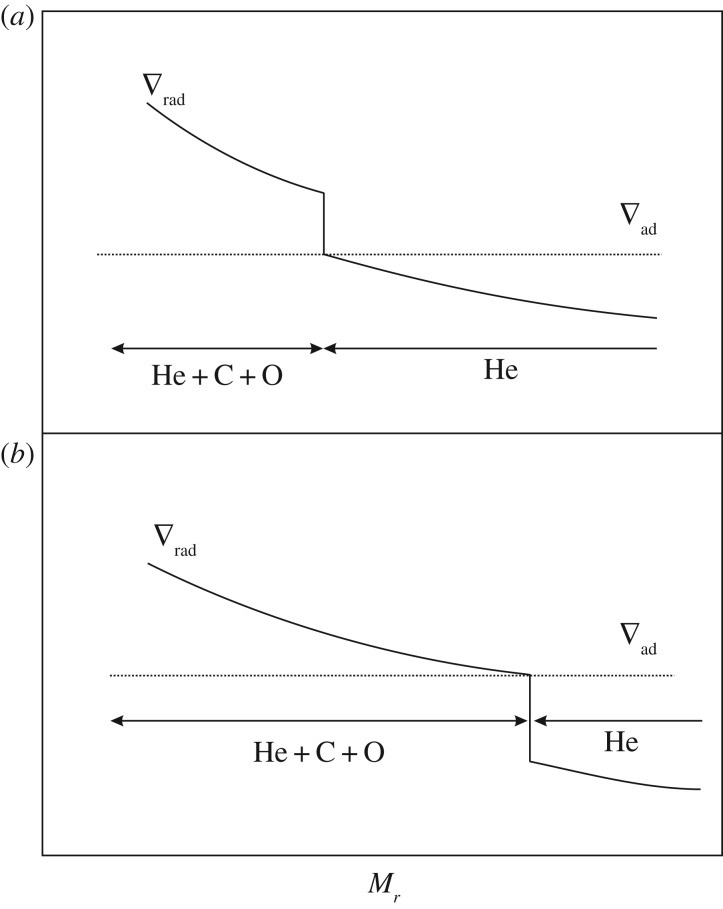
Similar to figure 8, qualitative sketch of the change in the fully mixed core when the discontinuity of ∇_rad_ at its edge is maintained (*a*), and when the convective boundary is set where ∇_rad_=∇_ad_ (*b*) (see text for details).

In practical terms, at every computational time step, one can place the boundary of the fully mixed convective region at the layer where ∇_rad_=∇_ad_ (figure 9).

**Figure 10.**
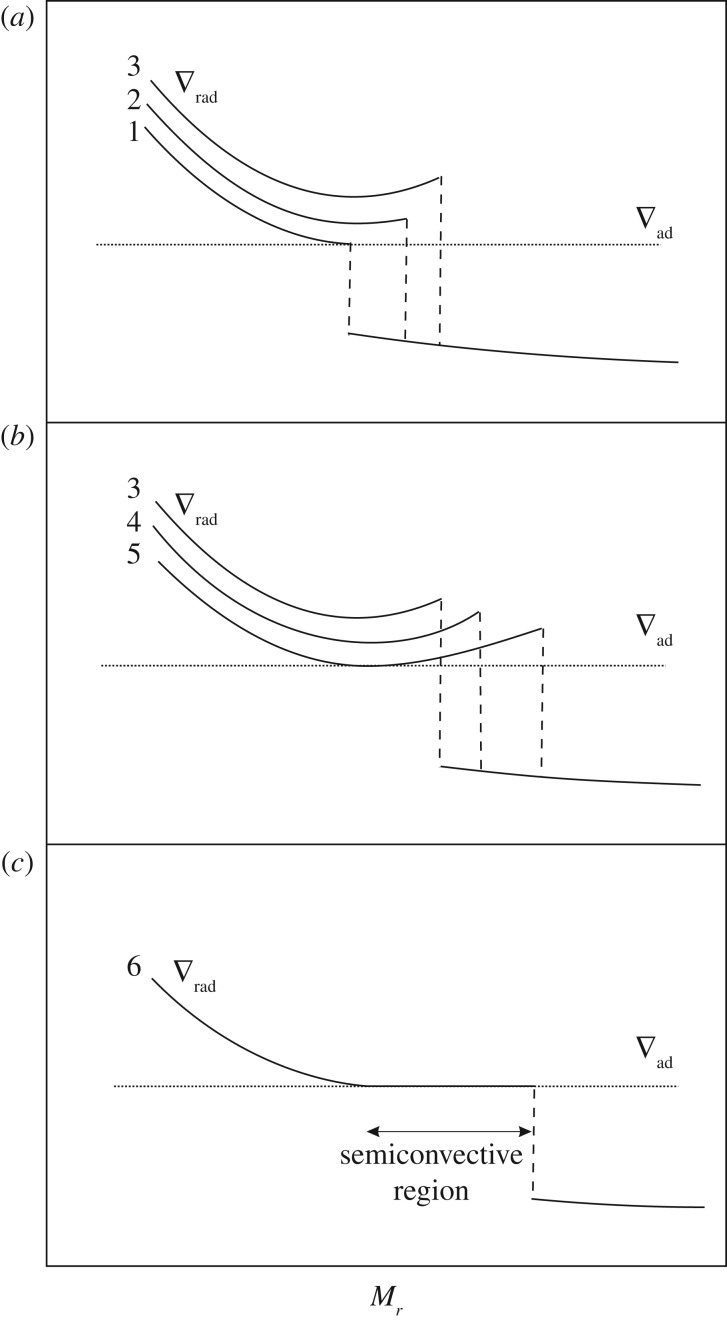
Similar to figure 9, sketch of the time evolution of the radiative gradient profile near the boundary of the convective core during the central He-burning stage. (*a*–*c*) The sequence of events which lead to the formation of the semiconvective zone (*c*). Increasing numbers denote a sequence of increasing time (see text for details).

After this early phase, when the extension of the convective core follows the increase of ∇_rad_, the radiative gradient profile starts to show a minimum (profile 3 in figure 10), as a consequence of the progressive outward shift of the convective boundary. The presence of this minimum depends on the complex behaviour of the physical quantities involved in the definition of ∇_rad_, such as opacity, pressure, temperature and local energy flux. In this situation, outward mixing to eliminate the ∇_rad_ discontinuity will induce a general decrease of the radiative gradient in the whole convective core (due to an average increase of He and consequent decrease of C—see profile 4 in figure 10). The radiative gradient will eventually decrease and become equal to ∇_ad_ at the location of the minimum of ∇_ad_ (profile 5 in figure 10).

**Figure 11.**
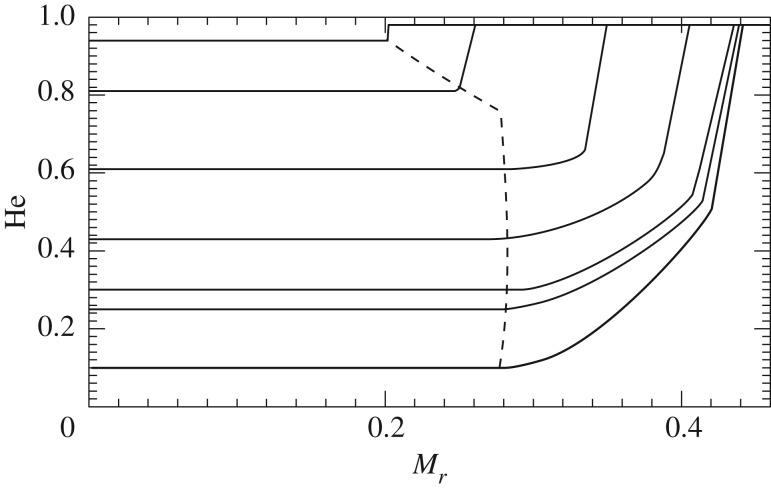
He-abundance profile inside the convective core and the semiconvective zone at various levels of central He depletion, for a low-mass core He-burning model. The dashed line marks the location of the fully mixed core boundary.

The semiconvective region is the region between the ‘neutral’ ∇_rad_=∇_rad_ point and the overlying formally convective shell whose upper boundary still displays a discontinuity in ∇_rad_. In fact, a full instantaneous mixing between the convective core located inside the minimum and the external convective shell would have the consequence of decreasing the radiative gradient in the whole mixed region. However, due to the presence of this minimum, ∇_rad_ in a portion of the mixed core would become lower than the ∇_ad_, i.e. it would not be convective. A solution for this inconsistency is to impose a partial mixing in the formally convective shell (see, e.g. [86] for an example of implementation), such that the final chemical composition—shown in figure 11—satisfies the condition ∇_rad_=∇_ad_ (profile 6 in figure 10). The mass location of the minimum of the radiative gradient moves outwards with time, because of the evolution of the chemical abundances caused by nuclear burning. As a final result, the C-enriched region increases its size outwards.

The effects of semiconvection on the evolution of these models are the following: the evolutionary tracks perform more extended loops in the HRD, and the central He-burning phase lasts longer because of a larger amount of fuel to burn.

When the central He abundance has decreased to about *Y* ∼0.10, *α*-captures by C nuclei tend to overcome C production by 3*α* reactions, thus He-burning becomes mainly a ^12^C+*α* production of oxygen, whose opacity is even larger than that of ^12^C. This causes an increase in the size of the semiconvective region and, in turn, more fresh helium is transferred into the core, which is now nearly He-depleted. Even a small amount of He added to the mixed core enhances the rate of energy production, thus the luminosity increases, driving an increase in the radiative gradient. As a consequence, a phase of enlarged mixed zone starts, the so-called *breathing pulse*. After this breathing pulse, the star readjusts to burn steadily in the core the fresh He driven there by semiconvection. Detailed calculations show that a few breathing pulses are expected before the complete exhaustion of He in the core. The evolutionary effects of the breathing pulses are the following: the models perform a loop in the HRD at each pulse, the He-burning lifetime is slightly increased and the mass of the CO-core at He exhaustion is increased.

Empirical constraints—mainly the number ratio between horizontal branch (HB—core He-burning phase in low-mass stars) and AGB stars in Galactic globular clusters (GGCs)—suggest that the efficiency of the breathing pulses phenomenon is very low, if any [87,88]. Therefore, they are usually inhibited in stellar model computations by using *ad hoc* numerical assumptions [82,89]. Typically, during the late stages of core He-burning one forces the extension of the mixed region not to lead to an increase in the central He abundance from one model to the next [87]. Another option is to set to zero the gravitational term in the energy generation equation for the inner regions. In this way, the breathing pulses are also effectively inhibited [82].

This brief discussion highlights clearly the difficulty in modelling core mixing in HB stars. Notice that the standard treatment is usually instantaneous mixing, which may not be adequate in a semiconvective regime. None of the diffusive semiconvective formalisms employed for semiconvection related to H-burning is usually applied to this situation, although the diffusive mixing employed in the STARS code still leads to the onset of breathing pulses during the core He-burning phase. It is also worth recalling that with the inclusion of an extended overshooting region (approx. 1 *H*_*P*_) beyond the layer where the ∇_rad_ discontinuity develops, the fully mixed core is always so large that the need to include semiconvective layers disappears [90] (breathing pulses still seem to appear also with large overshooting). An important consequence of including semiconvection, or large overshooting, and/or breathing pulses, is that the CO profile in the final CO core changes, with important consequences for the cooling times of the final WD stage.^10^

With increasing stellar mass, the weight of the *free–free* opacity decreases (because of higher core temperatures) and eventually all these problems disappear in the regime of intermediate-mass stars (masses above a few solar masses).

## 5. Thermohaline mixing

**Figure 12.**
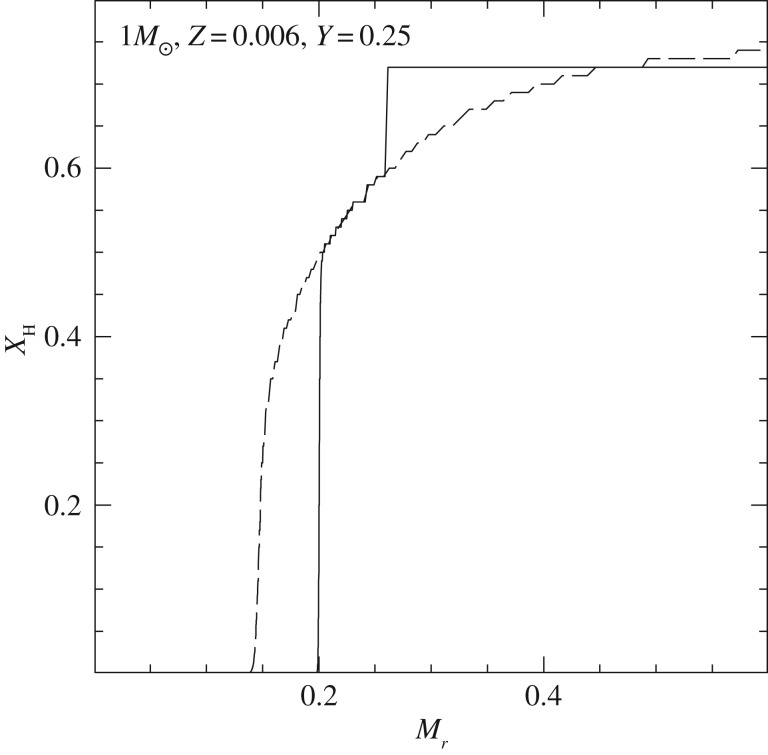
H-abundance profile within a low-mass stellar model at the start (dashed line) and at the completion (solid line) of the first dredge-up, respectively. The horizontal coordinate displays the local value of the mass. At the completion of the dredge-up, the He-core mass is equal to approximately 0.2*M*_⊙_ (it was approx. 0.14*M*_⊙_ at the start of the dredge-up) and the H-abundance discontinuity left over by the fully mixed convective envelope at its maximum extension is located 0.25*M*_⊙_ away from the centre. The varying H abundance in the layers between the He-core and the discontinuity has been produced during core H-burning along the MS. When the H-burning shell reaches the H-abundance discontinuity the RGB model experiences a temporary drop in luminosity (that in a stellar population produces a local increase in star counts, the so-called ‘RGB bump’, before evolving again with increasing *L* when the discontinuity is crossed.

In recent years, the role played by thermohaline mixing in stellar evolution has been widely explored in connection with low-mass RGB evolution.^11^ When the convective envelope deepens after the TO, the surface chemical composition is altered due to the dredge-up of H-burning processed matter—the so-called *first dredge-up*—that increases the abundance of N and He, and decreases C and the ^12^C/^13^C ratio. The first dredge-up is completed when the convective region reaches its maximum extension, approaching closely (but not reaching) the H-burning shell around the inert (and electron degenerate) He-core. From this moment on the receding convective boundary is not expected to modify further the surface abundances along the RGB. Spectroscopic observations of metal-poor Galactic halo stars provide, however, compelling evidence for an additional mixing process occurring when RGB stars reach the luminosity of the RGB bump (figure 12), which causes a sudden drop of the isotopic ratio ^12^C/^13^C, a decrease in Li and C, and an increase in N. This mixing affects approximately 95% of low-mass stars, regardless of whether they populate the halo field or clusters [94,95], and thermohaline mixing has been proposed to explain these observations.^12^

In low-mass stars, the main H-burning mechanism during the MS is the *p-p chain* that, due to its weak dependence on temperature, is efficient also in stellar layers far from the star centre. As a consequence, ^3^He accumulates in a broad zone outside the main energy production region. During the first dredge-up, this ^3^He is mixed within the convective envelope, with the consequence that, during the following RGB evolution, the layers above the H-discontinuity left over by the receding convective envelope at its maximum extension will have a uniform ^3^He abundance, larger than the initial one.

When the shell advances towards the surface during the RGB evolution, in the outer wing above the point of maximum burning efficiency there is a narrow region where ^3^He is processed through the reaction ^3^He(^3^He,2*p*)^4^He. In this nuclear reaction two nuclei transform into three and the mean mass per nucleus—the molecular weight *μ*—decreases. This leads to a small local decrease of *μ* when moving from the surface towards the centre of the star. As long as the H-burning shell advances through layers below the H-discontinuity—when the star evolves before the RGB bump, see figure 12—this effect is negligible because the shell is moving in a region with a large positive gradient, due to the H profile left over at the end of the MS. However, when the H-burning shell enters the region of uniform H abundance above the discontinuity, the local inversion of the *μ* profile, of the order of one part in 10^4^, becomes important [97]. This situation corresponds to the conditions for thermohaline mixing (see [98] and references therein).

Thermohaline mixing is usually included in stellar evolution codes as a diffusive process that tends to erase the molecular weight inversion, with a diffusion coefficient derived from a linear analysis [99,100]

5.1 $$D_{\mathrm{th}}=C_{\mathrm{th}}\frac{K}{c_{p}\rho }\left(\frac{\phi }{\delta }\right)\frac{\nabla_{\mu }}{\left(\nabla_{\mathrm{rad}}-\nabla_{\mathrm{ad}}\right)},$$

where $C_{\mathrm{th}}=(\frac{8}{3})\pi^{2}\alpha^{2}$, *α* being a free parameter related to the aspect ratio (length/width) of the mixing elements.^13^

**Figure 13.**
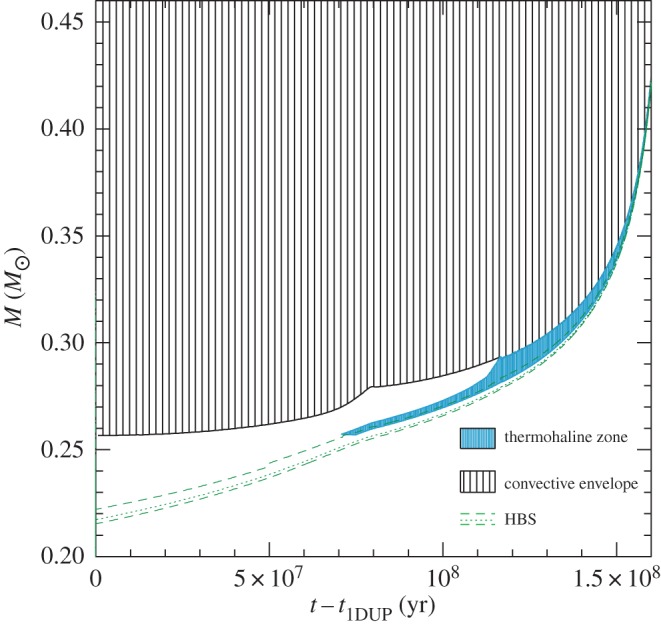
Location of the mass boundaries as a function of time (zero point taken at the completion of the first dredge-up) of the convective envelope, H-burning shell and the zone mixed by the thermohaline instability, for a low-mass RGB stellar model.

As shown in figure 13, evolutionary calculations show that thermohaline mixing extends between the outer wing of the H-burning shell and the inner boundary of the convective envelope, merging with the outer convection in a short time (approx. 30 Myr for a model with mass of the order of 1*M*_⊙_). Therefore, depending on the mixing efficiency (hence the choice of *C*_th_), a significant amount of nuclear-processed matter in the hotter layers of the H-burning shell can be dredged up to the surface during the remaining RGB evolution, helping to explain the spectroscopic data.

Comparisons with observations require *C*_th_∼100−300 from Li observations in GGCs, and *C*_th_∼1000 for C in globulars, clearly mutually inconsistent. On the other hand, models in [92,98] are able to match the ^12^C/^13^C isotopic ratio, C and N abundances in halo RGB field stars with *C*_th_∼1000; the same choice of *C*_th_ allows also to match measurements of the carbon isotopic ratio and the [N/C] ratio in open clusters, and Li abundances in field disc stars. Results from 3D hydrodynamics simulations (rescaled to match stellar conditions) add an additional source of uncertainty, for they predict an efficiency equivalent to just *C*_th_≈10 [102,103].

A recent detailed numerical analysis [101] has shown that the surface chemical abundances predicted by stellar models accounting for thermohaline mixing depend on numerical assumptions like spatial and time resolution adopted in the computations. As a consequence, the predicted surface chemical abundances should be treated with caution until a firmer assessment on how to treat thermohaline mixing in model computations is achieved.

## 6. Atomic diffusion

Microscopic effects related to collisions among the gas particles induce a *slow* element transport within radiative regions. It is possible to show from first principles that individual ions are forced to move under the influence of pressure as well as temperature gradients, which both tend to displace the heavier elements towards the centre of the star, and of concentration gradients that oppose the above processes. Radiation—which does not have a major effect in the Sun [104]—pushes the ions towards the surface, whenever the radiative acceleration imparted to an individual ion species is larger than the gravitational acceleration. The speed of the diffusive flow depends on the collisions with the surrounding particles, as they share the acquired momentum in a random way. It is the extent of these ‘collision’ effects that dictates the timescale of element diffusion within the stellar structure, once the physical and chemical profiles are specified.

The most general treatment for the element transport in a multi-component fluid associated with diffusion is provided by the Burgers equations [105]. They are obtained assuming the gas particles have approximate Maxwellian velocity distributions, the temperatures are the same for all particle species, the mean thermal velocities are much larger than the diffusion velocities and magnetic fields are unimportant, and they can be written as

6.1 $$\frac{dp_{i}}{dr}+\rho_{i}(g-g_{\mathrm{rad},i})-n_{i}{\bar{Z}}_{i}eE=\sum_{j\neq i}^{N}K_{ij}(w_{j}-w_{i})+\sum_{j\neq i}^{N}K_{ij}z_{ij}\frac{m_{j}r_{i}-m_{i}r_{j}}{m_{i}+m_{j}},$$

including the heat flow equations,

6.2 $$\begin{array}{rl} \frac{5}{2}n_{i}K_{B}\nabla T & =\frac{5}{2}\sum_{j\neq i}^{N}z_{ij}\frac{m_{j}}{m_{i}+m_{j}}(w_{j}-w_{i})-\frac{2}{5}K_{ii}z_{ii}^{\prime \prime }r_{i}\\ & \;-\sum_{j\neq i}^{N}\frac{K_{ij}}{{\left(m_{i}+m_{j}\right)}^{2}}(3m_{i}^{2}+m_{j}^{2}z_{ij}^{\prime }+0.8m_{i}m_{j}z_{ij}^{\prime \prime })r_{i}\\ & \;+\sum_{j\neq i}^{N}\frac{K_{ij}m_{i}m_{j}}{{\left(m_{i}+m_{j}\right)}^{2}}(3+z_{ij}^{\prime }-0.8z_{ij}^{\prime \prime })r_{j}. \end{array}$$

In addition, there are the constraints of electric current neutrality,

6.3 $$\sum_{i}{\bar{Z}}_{i}n_{i}w_{i}=0$$

and local mass conservation,

6.4 $$\sum_{i}m_{i}n_{i}w_{i}=0.$$

In the above 2*N*+2 equations *p*_*i*_, *ρ*_*i*_, *n*_*i*_, ${\bar{Z}}_{i}$ and *m*_*i*_ denote the partial pressure, mass density, number density, mean charge and mass for species *i*, respectively. The total number of species (including electrons) is *N*. The 2*N*+2 unknown variables are the *N* diffusion velocities *w*_*i*_, the *N* heat fluxes *r*_*i*_, the gravitational acceleration *g* (the comparison of the derived *g* with the known value from the integration of the stellar structure equations provides an important check for the consistency of the results) and the electric field *E*. The coefficients *K*_*ij*_, *z*_*ij*_, $z_{ij}^{\prime }$ and $z_{ij}^{\prime \prime }$ have to be specified, together with the radiative accelerations *g*_rad_. Several stellar evolution codes—in one form or another—use the routine by Thoul *et al*. [106] to solve the Burgers equations and calculate the velocities of the various chemical species. These diffusion velocities can then be inserted as an advection term in the equation for the time evolution of the mass fraction abundance *X*_*i*_

6.5 $${\frac{\partial X_{i}}{\partial t}|}_{M_{r}}=-\frac{1}{\rho r^{2}}\frac{\partial }{\partial r}(\rho r^{2}X_{i}w_{i}),$$

where we show on the right-hand side just the contribution of diffusion to the time evolution of the abundance *X*_*i*_ of element *i*.

In Burgers’ formalism, the effect of collisions between ions is represented by the so-called resistance coefficients, i.e. the matrices *K*, *z*, *z*^′^, *z*^′′^, whose precise evaluation is essential to estimate correctly the diffusion timescales for the various elements. These resistance coefficients can be expressed in terms of the so-called reduced collision integrals ${\Omega_{ij}^{\left(l,s\right)}}^{*}$ according to the following relationships:

6.6 $$\begin{array}{r} \frac{K_{ij}}{K_{ij}^{0}}=4\frac{{T_{ij}^{*}}^{2}{\Omega_{ij}^{\left(1,1\right)}}^{*}}{\ln(\Lambda_{ij}^{2}+1)}, \end{array}$$

6.7 $$\begin{array}{r} z_{ij}=1-1.2\frac{{\Omega_{ij}^{\left(1,2\right)}}^{*}}{{\Omega_{ij}^{\left(1,1\right)}}^{*}}, \end{array}$$

6.8 $$\begin{array}{r} z_{ij}^{\prime }=2.5-\frac{6{\Omega_{ij}^{\left(1,2\right)}}^{*}-4.8{\Omega_{ij}^{\left(1,3\right)}}^{*}}{{\Omega_{ij}^{\left(1,1\right)}}^{*}} \end{array}$$

6.9 $$\begin{array}{r} \;\text{and}\;z_{ij}^{\prime \prime }=2\frac{{\Omega_{ij}^{\left(2,2\right)}}^{*}}{{\Omega_{ij}^{\left(1,1\right)}}^{*}}, \end{array}$$

where

6.10 $$T_{ij}^{*}=K_{B}T\frac{\lambda_{D}}{|Z_{i}Z_{j}e^{2}|}$$

and

6.11 $$K_{ij}^{0}=\frac{2}{3}\sqrt{\frac{2\mu_{ij}\pi }{{\left(K_{B}T\right)}^{3}}}{\left(Z_{i}Z_{j}e^{2}\right)}^{2}n_{i}n_{j}\ln(\Lambda_{ij}^{2}+1),$$

with λ_D_ being the Debye length; $\Lambda_{ij}=4T_{ij}^{*}$ the plasma parameter (*ln*(*Λ*_*ij*_) is called *Coulomb logarithm*); *μ*_*ij*_=*m*_*i*_*m*_*j*_/(*m*_*i*_+*m*_*j*_) the reduced mass; and *Z*_*i*_, *m*_*i*_ and *n*_*i*_ the charge number, mass and particle number density of species *i*, respectively.

The collisions between particles of species *i*,*j* in the stellar plasma determine the values of the ${\Omega_{ij}^{\left(l,s\right)}}^{*}$ integrals; the physics of the collisions is specified by some form of the Coulomb interaction. This, as a first approximation, can be described by a pure Coulomb potential with a long-range cut-off distance, typically equal to λ_D_. Using this truncated pure Coulomb potential, the resistance coefficients originally computed by Burgers become [105]

6.12 $$\begin{array}{r} \frac{K_{ij}}{K_{ij}^{0}}=2\frac{\ln\;\Lambda_{ij}-C_{E}\pm \pi^{2}/4}{\ln(\Lambda_{ij}^{2}+1)}, \end{array}$$

6.13 $$\begin{array}{r} z_{ij}=0.6, \end{array}$$

6.14 $$\begin{array}{r} z_{ij}^{\prime }=1.3 \end{array}$$

6.15 $$\begin{array}{r} \;\text{and}\;z_{ij}^{\prime \prime }\approx 2, \end{array}$$

where *C*_E_ is Euler’s constant and the ± signs denote repulsive (+) and attractive (−) pairs of particles, respectively. More accurate collision integrals have been obtained by considering a Debye–Hückel type of potential

**Figure 14.**
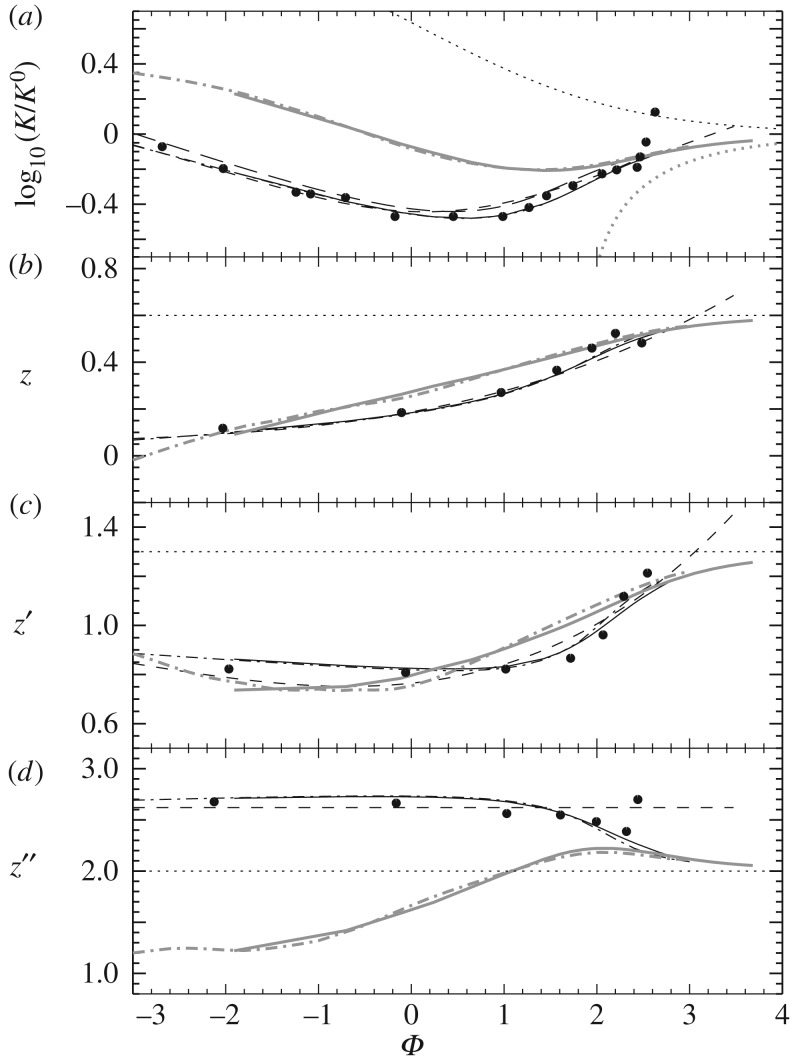
Resistance coefficients *K* (*a*), *z* (*b*), *z*^′^ (*c*) and *z*^′′^ (*d*) as originally computed by Burgers [105] (dotted lines), [107] (solid lines), [108] (short-dashed line), [110] (dashed-dotted lines) and [109] (long-dashed line) as a function of $\Phi_{ij}=\ln(\ln(1+\Lambda_{ij}^{2}))$. Dark black lines denote the case of a repulsive potential, while the brighter ones denote attractive forces (not computed in [108,109]). The dots represent the actual values computed by Muchmore [108], not his fitting formulae.

6.16 $$V_{ij}(r)=\frac{Z_{i}Z_{j}e^{2}}{r}\;e^{-r/\lambda_{D}},$$

where *r* is the particle distance. The results are provided either in tabulated form [107] or as fitting formulae [108–112], and some of them are compared in figure 14, as a function of $\Phi_{ij}=\ln(\ln(1+\Lambda_{ij}^{2}))$. Given that the calculations shown in the figure (apart from Burgers’ results) make all the same physical assumptions, results are similar, but they all differ significantly from Burgers’ results when moving towards higher densities (lower values of *Φ*_*ij*_), where his approximations are no longer adequate.

Some of these authors argued that while λ_D_ is a suitable screening distance at low densities, the Debye sphere loses its significance in denser plasmas, and that a more appropriate screening distance is in this case the mean interionic distance. Hence, they suggested to use the larger value between λ_D_ and the mean interionic distance (in the Sun, the former has always been larger than the latter). Whatever the choice for the actual screening distance, its value has to be employed as λ_D_ in equation (6.10) to compute the appropriate *Λ*_*ij*_ for determining the collision integrals. The effect of quantum corrections on the resistance coefficients has been included in [111] while the calculations [112] account for very recent developments in ionic transport properties in strongly coupled plasmas [113].

As for the radiative levitation of element *i*, the main physical interactions that drive the transfer of momentum from the radiation field to ions are *bound–bound* and *bound–free* transitions, while it is practically ineffective for fully ionized elements. The radiative acceleration can be written as [114]

6.17 $$g_{\mathrm{rad},i}=\frac{\mu \kappa }{\mu_{i}c}\frac{l}{4\pi r^{2}}\gamma_{i}$$

where *μ* is the mean atomic weight, *μ*_*i*_ is the atomic weight of element *i*, *l* is the local luminosity and *r* is the radius [114]. The dimensionless quantity *γ*_*i*_ depends on the monochromatic opacity data

$$\gamma_{i}=\int \frac{(\sigma_{i}(u)[1-\exp(-u)]-a_{i}(u))\;du}{\sum_{i}f_{i}\sigma_{i}(u)},$$

where *u*=(*hν*)/(*K*_B_*T*), *σ*_*i*_ is the cross section for absorption or scattering of radiation by element *i*, *a*_*i*_ accounts for the fact that in *bound–free* transitions only a fraction of the momentum of the ionizing photons is transferred to the ion, the rest being transferred to the electron lost by the ion, and *f*_*i*_ is the number fraction of element *i*. The Opacity Project provides a set of codes called OPSERVER that enables the calculation of *g*_rad,*i*_ [115].

Calculations of precise *g*_rad,*i*_ values involve carrying out the integration over about 10^4^ *u* values for each atomic species [116]. Given that these calculations have to be repeated at each layer in the stellar model and at each time step during the computation of an evolutionary sequence, this explains why, to date, there are only few extended sets of stellar models that include also the effect of radiative levitation. One has also to note that the Rosseland mean opacity entering the stellar structure equations as well as equation (6.17) has to be continuously recalculated at each mass layer, not just in the nuclear-burning regions—this is in principle true also for calculations including only the other diffusive processes listed above—to be fully consistent with the composition changes.

**Figure 15.**
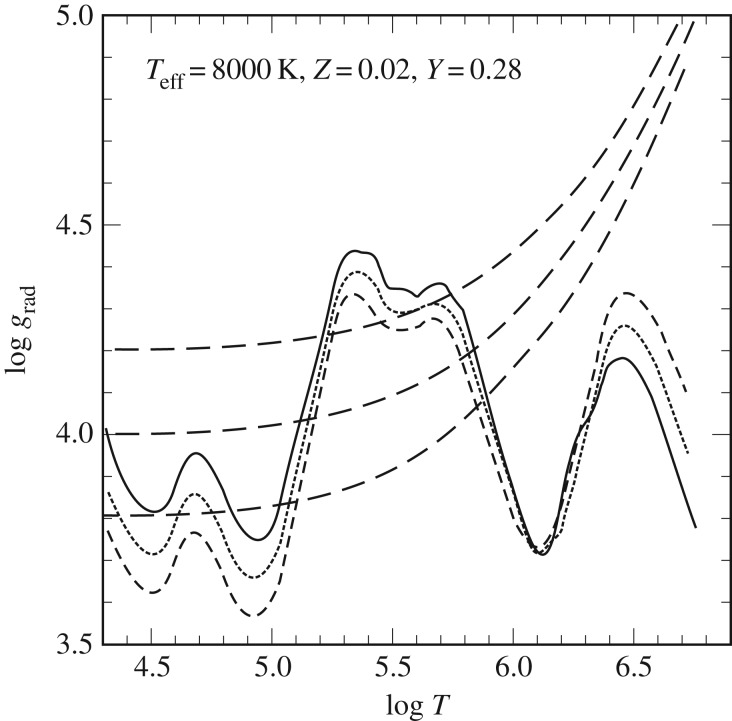
Total radiative acceleration of Fe as a function of the temperature in a stellar envelope with the labelled *T*_eff_ and chemical composition, for three values of the surface gravity log(*g*), equal to 3.8 (short-dashed line), 4.0 (dotted line) and 4.2 (solid line), respectively. The run of the local gravity with temperature is displayed by long-dashed lines.

**Figure 16.**
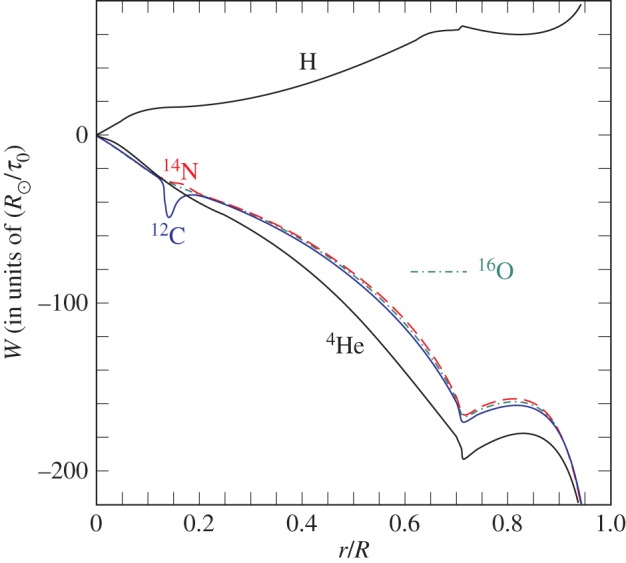
Diffusion velocity of H, He, C, N, O (the lines displaying the diffusion velocities of C, N and O almost overlap), as a function of the local fraction of the total radius, within a solar model. The velocity is in units of solar radius over the typical diffusion timescale for the Sun, that is of the order of 6×10^13^ yr. The base of the convective envelope is at *R*/*R*_⊙_∼0.71.

As an example, figure 15 displays the total radiative acceleration of iron in a stellar envelope. When the radiative acceleration is larger than gravity below a convective envelope—if convection is present, the effect is obviously negligible because of the much faster timescales of convective mixing—the element can diffuse towards the surface. Figure 16 displays the velocity profiles of H (moving upwards), He and CNO (sinking) within a solar model, derived from the solution of the Burgers equations.

The diffusion velocities are always dominated by the effect of pressure gradients. The radiative (upwards) acceleration of C, N and O is only at most about 5% of the local acceleration of gravity just below the convective envelope boundary [104], and decreases fast moving towards the centre. The local sharp increase of the settling velocity of C and the corresponding small decrease for N at *r*/*R*∼0.15–0.20 are due to the effect of abundance gradients when these elements attain the equilibrium abundances of the CN cycle (C decreases, while N increases).

### 6.1. The effect of atomic diffusion on stellar models

Atomic diffusion (sometimes denoted as microscopic diffusion, and hereafter simply diffusion) has a major direct effect on the MS evolution, the chemical stratification of the external layers of hot horizontal branch stars^14^ and WDs, and the internal chemical stratification of cold WDs. However, some properties of other evolutionary phases are also indirectly affected, as discussed below [84,117–121].

The impact of diffusion on stellar models will be discussed mainly in the context of low-mass stars, due to their evolutionary timescales comparable to the diffusion timescales during the MS phase. One has also to take into account that massive hot stars, where in principle radiative levitation can be extremely efficient, experience strong mass loss and rotational mixings (see later on in this section and §8) that tend to limit or completely erase the effect of diffusion.

If we consider the evolution of a typical low-mass star with a convective envelope on the MS, the surface abundances of metals and He tend to decrease because of diffusion from the bottom boundary of the shrinking convective envelope (that maintains a uniform chemical profile due to the shorter convective timescales compared to diffusion) being replaced by hydrogen. However, if the radiative acceleration on some ion species is larger than the local gravitational acceleration below the convective envelope, these elements are slowly pushed into the convective zone and their surface abundance increases. In general, the variation of the surface abundances during the MS phase is dictated by the interplay between radiative levitation and the sedimentation due mainly to pressure gradients (often denoted as *gravitational settling*). This variation of the surface abundances reaches a maximum around the TO.

When, after the TO, the convective envelope starts to deepen, it will rehomogenize an increasingly larger fraction of the stellar mass. Once the star reaches the RGB and the first dredge-up is completed, the abundance changes previously developed are almost completely erased, apart for the very inner layers, where metals and He were sinking during the MS. In general, the smaller (in mass) the convective envelope, the larger is the change of surface abundances during the MS, because of the smaller diluting *buffer* of matter with the initial chemical composition.

**Figure 17.**
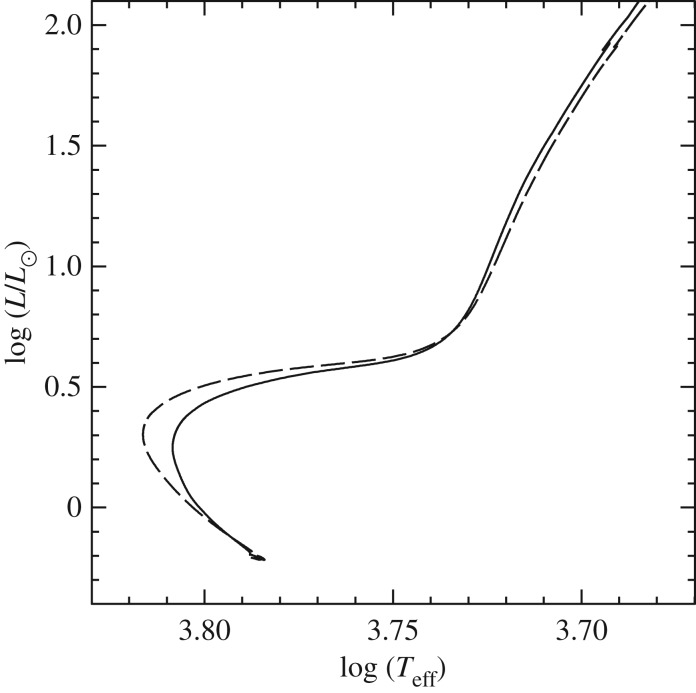
Comparison of the HRD of two 0.82*M*_⊙_, *Y* =0.25, *Z*=0.001, evolutionary tracks calculated with (solid line) and without (dashed line) atomic diffusion.

Figure 17 compares the HRD of low-mass, metal-poor evolutionary tracks, calculated with and without the inclusion of atomic diffusion. The track with diffusion increasingly diverges from the no-diffusion one moving along the MS. Its TO is fainter and cooler, while the two tracks coverge again along the RGB, where the effect of diffusion is practically erased by the fully mixed deep convective zone. The MS evolutionary times are also different, for the track with diffusion has a shorter lifetime because of the slow increase of He at the centre at the expenses of H during the MS phase.^15^ These changes of TO luminosity and MS lifetime at a given stellar mass affect also theoretical isochrones, hence age determinations of old stellar populations based on the TO luminosity decrease by about 10%.

**Figure 18.**
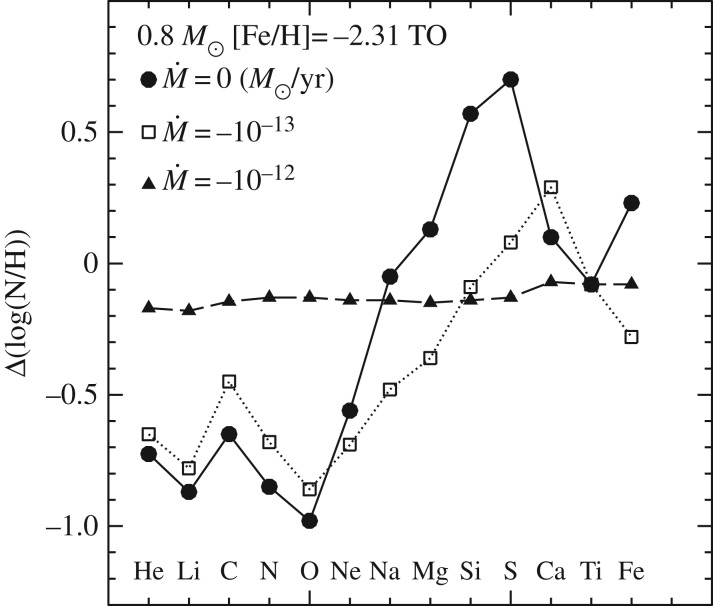
Difference between the TO surface abundances of the most relevant elements, and their initial values, for a low-mass metal-poor model (see labels). The element abundances are given as log(N/H), e.g. as logarithms of their number fraction to hydrogen [122]. These abundance variations are shown for models calculated with no mass loss, and two different mass loss rates during the MS evolution.

Figure 18 shows the pattern of variations of TO surface abundances, for a typical stellar model at the TO of a metal-poor globular cluster. These models have thin convective envelopes that maximize the effect of diffusion.

It is straightforward to notice the selective effect of diffusion, due to radiative levitation. Elements like He, Li, C, N and O sink below the convective layers and their surface abundances are severely depleted up to a factor of about ten. Other elements like Mg, Si, S and Fe are pushed into the convective envelope by the radiation pressure and their surface abundances increase during the MS. The size of these abundance variations decrease in models with larger convective envelopes. For example, a 0.8*M*_⊙_ model with initial [Fe/H]=−0.7 will show uniformly depleted abundances for all elements heavier than H at the TO, at a level of just 0.1–0.15 dex.

After the first dredge-up is completed, these abundance variations basically disappear. The He abundance post dredge-up is slightly lower in the model with diffusion (Δ*Y* ∼0.01), the RGB bump magnitude decreases by approximately 0.07 mag, the He-core mass at the He-flash increases by a few 0.001*M*_⊙_. As a consequence, the RGB tip luminosity increases at the level of just approximately 0.01 mag. The abundances of metals (other than CNO) in the He-core are only a few 0.01 dex more abundant than originally, while they (and He) are less abundant than originally by about the same amount around the core.

**Figure 19.**
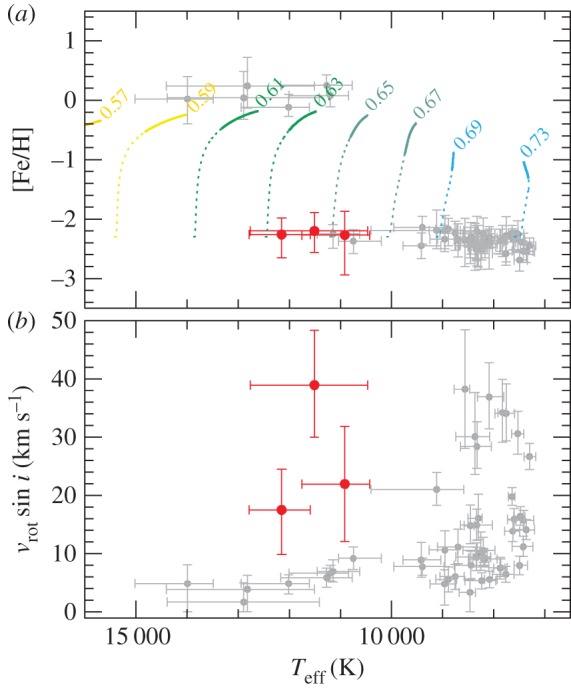
(*a*) Displaying the surface [Fe/H] values measured in a sample of stars in the metal-poor GGCs M15, M68 and M92, as a function of their *T*_eff_. The evolution (dotted segments for an HB age between 0 and 10 Myr, solid segments for ages between 30 and 60 Myr) of the surface [Fe/H] for HB models with the labelled masses including diffusion is also displayed [120]. (*b*) Displaying the projected rotation velocities for the cluster stars in the top panel. The red points (with error bars) identify stars with a different behaviour regarding the correlation between *T*_eff_, rotation velocity and surface [Fe/H] (see text for details).

Moving to the following HB phase, the zero age horizontal branch (ZAHB) luminosity decreases by approximately 0.02 mag compared to the no-diffusion case, driven by the lower amount of He in the envelope, but the major effect is on the surface abundances during the following HB evolution, as shown in figure 19. At *T*_eff_ above approximately 6000 K, the surface chemical composition of the models starts to be altered by diffusion; the selective effect increases during the HB evolution and is more pronounced for lower HB masses that evolve at higher *T*_eff_. The abundance of He tends to decrease, while overall the surface metal content increases. Figure 19 displays, as an example, the predicted behaviour of the surface Fe abundance in metal-poor HB models. One can notice an increase in surface Fe by a factor of approximately 10 within the first 10 Myr, and up to a factor of approximately 100 after 60 Myr.

These surface abundance changes are again erased by the deepening envelope convection for all HB masses that evolve to the following AGB phase. The subsequent hot WD phase sees diffusion altering again in a major way the surface abundances. Owing to the high surface gravity, gravitational settling of metals (down to the edge of the electron degenerate CO core) in the doubly layered H and He (DA type) or He-dominated (non-DA type) envelopes is very fast. Timescales depend on the dominant element, the envelope thickness, *T*_eff_ and gravity, but they are at most of the order of 10^6^ yr [119]. Finally, in cool WDs, when the electron degenerate CO core is in the liquid phase, the slow gravitational settling of ^22^Ne lenghtens the cooling times of faint WDs from high metallicity progenitors by up to approximately 1 Gyr, about 10% or more of the total cooling time of the models [123–127]. This element is the most abundant *impurity* present in the CO core, and originates from ^14^N during the core He-burning phase. Owing to the two additional neutrons hosted by the ^22^Ne nucleus relative to ^12^C and ^16^*O* ions, this element experiences a net downward gravitational force and slow settling in the liquid region of the core (inhibited in the solid regions, due to the expected sudden increase of viscosity). This regime is much denser than the case treated by the Burgers equations, and the ^22^Ne diffusion velocity *w*_^22^Ne_ is determined from a diffusion coefficient *D*_^22^Ne_ estimated from molecular dynamics calculations for a strongly coupled plasma [128]:

$$w_{{\;}^{22}\mathrm{Ne}}=\frac{2m_{p}gD_{{\;}^{22}\mathrm{Ne}}}{K_{B}T},$$

where *m*_*p*_ is the proton mass and *g* is the local acceleration of gravity.

The most recent estimate of the diffusion coefficient *D*_^22^Ne_ in the liquid phase provides

6.18 $$D_{{\;}^{22}\mathrm{Ne}}∼D_{0}\;0.53{\left[\frac{\bar{Z}}{Z_{{\;}^{22}\mathrm{Ne}}}\right]}^{2/3}(1+0.22\Gamma )\exp(-0.135\Gamma^{0.62}).$$

In this equation, $\bar{Z}$ denotes the average atomic number of the degenerate core chemical composition, and *Γ* the Coulomb parameter (1<*Γ*<180 in the liquid phase) defined as

6.19 $$\Gamma =\frac{\bar{Z^{5/3}}e^{2}}{a_{e}T},$$

Here, $\bar{Z^{5/3}}$ is an average over the ion charges, *T* is the temperature and the electron sphere radius *a*_e_ is equal to (3/4*πn*_e_)^1/3^ with $n_{e}=\bar{Z}n$ the electron density and *n* the ion density.

In addition,

$$D_{0}=\frac{3\omega_{p}a^{2}}{\Gamma^{4/3}}$$

with *ω*_*p*_ the plasma frequency

$$\omega_{p}={\left[\frac{4\pi e^{2}{\bar{Z}}^{2}n}{\bar{M}}\right]}^{1/2},$$

with $\bar{M}$ denoting the average mass of the ions.

The diffusion of ^22^Ne increases the energy budget of the WD (hence increases the cooling times) through the contribution (∂*U*/∂*μ*)_*T*,*V*_(∂*μ*/∂*t*) to the gravitational energy generation coefficient *ϵ*_g_ in the equations of stellar structure (see equation (2.5)).

Moving to stars with initial masses in the range approximately 1.5–3.0*M*_⊙_, which display vanishing convective surface layers during the MS, calculations with diffusion show the development of surface abundance variations already during the pre-MS [129]. During the MS phase, they display large surface abundance variations (like He and Ca underabundances, iron-peak element overabundances); moreover, local enhancements of iron and nickel abundances below the thin surface convective layers lead to extra convective zones that, in some cases, can trigger pulsations through the iron-induced *κ*-mechanism [130].

### 6.2. Inhibition of the efficiency of atomic diffusion

As already mentioned, diffusion is an element transport mechanism that comes from basic physics principles, and should be efficient in stars. Helioseismic observations tell us that diffusion is efficient in the Sun; its inclusion in stellar models improves the match of the inferred sound speed profile, as well as the present depth of the convective envelope and its He abundance [131,132]. The very efficient diffusion of metals from the WD envelopes is also confirmed by observations, whereby for the vast majority of WDs the chemical composition of the surface is either pure H or pure He.^16^ Also the diffusion of Ne in the CO liquid cores of WDs is indirectly favoured by the comparison of TO and WD ages for the old metal-rich open cluster NGC6791. The effect of Ne diffusion increases the age obtained from the WD luminosity function, ensuring agreement with the TO age [126].

However, existing spectroscopic measurements of surface element abundances (e.g. Fe) in a sample of GGCs contradict the predictions of stellar models that include uninhibited diffusion [120,133–136]. In a star cluster, the initial mass of the stars evolving in post-MS stages is essentially constant, and measurements of chemical abundances at the TO and the base of the RGB should display a difference, due to the effect of diffusion on evolutionary tracks discussed before, which is maximized at the TO and essentially disappears along the RGB. For example, in the case of NGC6397, with [Fe/H]∼−2.1 along the RGB, the TO Fe abundance should be approximately 0.3 dex lower, and the Ca abundance approximately 0.1 dex larger than on the RGB according to models calculated with diffusion; observations show instead TO Fe and Ca abundances approximately 0.15 and approximately 0.1 dex lower than on the RGB, respectively [137].

This points to a reduction of the effect of diffusion on the surface abundances by some competing mechanism. Nothing of course can be said about the efficiency of diffusion in the inner layers.^17^ The same qualitative result is found in more metal-rich clusters like NGC6752 and M4, and also on the HB of a number of clusters, as shown by figure 19. In this figure, one can clearly see that for HB stars hosted by three metal-poor GGCs, the surface Fe abundance increases abruptly when *T*_eff_ goes above approximately 11 000 K, while theory predicts Fe enhancements (compared with the initial value [Fe/H]=−2.3 dex typical of these clusters) at much lower temperatures.

Also comparisons of models in the mass range between approximately 1.5 and 3.0*M*_⊙_, calculated including diffusion with surface abundances measured in A and F stars, show that diffusion must be moderated by some competing mechanism [129,139,140]. For example, in samples of A and F stars belonging to the Hyades, Pleiades and Coma Berenices Galactic clusters, observed abundances of elements like Na, Fe and Ni display a general pattern and star-to-star scatters that point to diffusion being moderated to different degrees by some competing process [141].

Finally, the observed constant surface Li abundance observed in field halo stars with *T*_eff_ above approximately 5500–6000 K and [Fe/H] below approximately −1.5 dex—the so-called *Spite plateau* [142]—is also problematic for models including diffusion, because they predict for these stars a progressive decrease of surface Li with increasing *T*_eff_, which is not observed [143,144].

#### 6.2.1. Rotational mixing

As we will see in §8, rotational mixing can, in principle, counteract the effect of diffusion, and this has been invoked to explain the *delayed* (in terms of *T*_eff_) onset of diffusion in hot HB stars [145]. This inference is related to the distribution of observed projected rotational velocities for the hot HB stars of figure 19, as shown in the lower panel of the same figure. All observed stars with *T*_eff_ above approximately 11 000 K that show spectroscopically the signature of diffusion are very slow rotators, whereas at lower *T*_eff_ rotation rates are on average higher. Estimates (not based on fully consistent stellar evolution models) of the competing effects of meridional circulation and diffusion, show that moderation/inhibition of diffusion below *T*_eff_∼11 000 K by rotational mixing is possible [145]. Interestingly, in support of this idea figure 19 shows three stars with *T*_eff_>11 000 K and large projected rotation velocities with no enhancement of surface Fe, at odds with the other slow-rotating objects at the same temperatures. The link between (partial) inhibition of diffusion and rotation is however probably not justified for A and F stars, which often show slow rotation velocities and surface abundances clearly affected by a reduced efficiency of diffusion, compared with the model predictions.

#### 6.2.2. Mass loss

Another process that can moderate diffusion in the stellar outer layers is mass loss [122,146,147]. Assuming that mass loss (hereafter ML) is spherical and unseparated (chemical composition of the wind equal to the photospheric composition), its effect is simply to *peel off* the outer layers of a model. Simple mass conservation constraints, coupled to the fact that the structure of the star is unaffected by the amount of mass lost between two consecutive computational time steps, translate into the appearance of an outward flowing interior velocity due to the wind [148] given by

6.20 $$v_{w}(r)=-\frac{\dot{M}}{4\pi r^{2}\rho }\frac{m_{r}}{M_{*}},$$

where *ρ* is the local density at radial distance *r* from the centre, *m*_*r*_ is the mass interior to *r*, M_*_ is the total mass and $\dot{M}$ (negative) is the mass loss rate.

Figure 18 displays the effect of ML on the surface abundances of a typical model at the TO of a metal-poor GGC. For elements affected mainly by gravitational settling, the effect of ML is to increase their surface abundance compared with the pure diffusion case. The reason is that with the chosen mass loss rates, below the convective envelope *v*_w_ counteracts the settling velocity, hence the degree of depletion is reduced. Larger negative values of $\dot{M}$ imply larger positive *v*_w_ and less diffusion from the envelope. In the case of elements supported by radiative levitation in some layers below the convective envelope, the effect of ML on the surface abundances is more complex, and this is due to the interplay between *v*_w_ and the depth at which *g*_rad_ is larger than the local gravitational acceleration *g*. For example, let us assume an ML rate $\dot{M}=-10^{-13}$ and an age of 10 Gyr; if *v*_w_ is larger than the settling velocity down to Δ*M*=10^−3^M_*_ from the surface, and in those layers *g*_rad,*i*_>*g* for a generic element *i*, after 10 Gyr the wind will have advected to the surface matter at that location, and the surface abundance of element *i* will be enhanced compared with the initial value.

This complex behaviour of the elements supported by radiative levitation can be seen in figure 18. For an element like Mg an ML rate $\dot{M}=-10^{-13}M_{⊙}\;{\mathrm{yr}}^{-1}$ transforms the surface overabundance of about 0.10 dex into an underabundance of about 0.4 dex. A further increase of $\dot{M}$ reduces the underabundance to about just 0.1 dex. In the case of Ca, the overabundance caused by diffusion is instead increased by an ML rate $\dot{M}=-10^{-13}M_{⊙}\;{\mathrm{yr}}^{-1}$, and then transformed into an underabundance when $\dot{M}=-10^{-12}M_{⊙}\;{\mathrm{yr}}^{-1}$.

ML rates of the order of ≈10^−12^*M*_⊙_ yr^−1^ are required to explain the Spite plateau and the behaviour of surface abundances in the metal-poor GGC NGC6397 [122,146]. These rates are unlikely, being much larger than the solar current mass loss rate $\dot{M}=-2\times 10^{-14}M_{⊙}\;{\mathrm{yr}}^{-1}$.

Rates of the order of ≈10^−13^*M*_⊙_ yr^−1^ are invoked to explain at least some of the surface abundance patterns seen in A and F stars [148].

#### 6.2.3. Thermohaline mixing

The modifications of the chemical abundance profiles caused by diffusion lead to variations of the local mean molecular weight. For example, He always sinks, introducing a stabilizing contribution to the local *μ*-gradient. On the other hand, heavy elements supported by radiative levitation will lead to a destabilizing *μ*-gradient (increasing outwards). If this second effect prevails, these layers are subject to thermohaline mixing (see §5), which will tend to erase the abundance changes, and hence to moderate the effect of diffusion.

Published calculations of models for A and F stars, including both diffusion and thermohaline mixing, show that thermohaline mixing (employing the diffusion coefficient derived by Brown *et al*. [149]) plays an important role in moderating the effect of diffusion on the surface abundances, although the surface abundances of the models do not yet match observations [140]. Both processes have been included also in calculations to explain the observed population of carbon-enhanced metal-poor stellar stars [150].

#### 6.2.4. Generic turbulence

A pragmatic solution adopted in several investigations is to add *by hand* some turbulence able to counteract the efficiency of atomic diffusion in the outer layers. Turbulence here means simply an additional *ad hoc* term in the chemical evolution equations that acts towards suppressing the development of chemical abundance gradients, not explicitly connected to a specific physical mechanism. Equation (6.5), which showed the contribution of diffusion to the time evolution of the abundance *X*_*i*_ of element *i*, is therefore modified to include an additional term

6.21 $${\frac{\partial X_{i}}{\partial t}|}_{M_{r}}=\frac{1}{\rho r^{2}}\frac{\partial }{\partial r}\left(D_{\mathrm{turb}}\rho r^{2}\frac{\partial X_{i}}{\partial r}\right)-\frac{1}{\rho r^{2}}\frac{\partial }{\partial r}(\rho r^{2}X_{i}w_{i}),$$

where *D*_turb_ is the chosen turbulent diffusion coefficient.

The literature presents at least three different choices for *D*_turb_. To date, the only formulation employed to interpret several different sets of data is the following [118,139,151]:

6.22 $$D_{\mathrm{turb},\mathrm{Rich}}=f\;D{\left(\mathrm{He}\right)}_{0}{\left(\frac{\rho }{\rho_{0}}\right)}^{n},$$

where

$$D{\left(\mathrm{He}\right)}_{0}={\left[\frac{3.3\times 10^{-15}T^{2.5}}{4\rho \ln(1+1.125\times 10^{-16}T^{3}\rho )}\right]}_{0}$$

is the He diffusion coefficient^18^ at a certain reference depth in the approximation of trace amount in an ionized hydrogen plasma. The subscript 0 indicates the reference depth in terms of either a reference density *ρ*_0_ or, more often, a reference temperature *T*_0_. In this latter case, *ρ*_0_ is the density at the layer where *T*=*T*_0_. The turbulent diffusion coefficient *D*_turb_ is *f* times the He diffusion coefficient at the reference depth 0, and varying as *ρ*^*n*^.

A choice of parameters *f*=400, log(*T*_0_)=5.5, *n*=−3 (denoted as T5.5D400-3) allows to match the abundances of A and F stars, while a T6.0D400-3 or T6.09D400-3 choice of parameters in equation (6.22) reproduces the flatness of the Li Spite plateau [118]. Spectroscopy of stars in NGC6397, NGC6752 and M4, three GGCs spanning a range of about 1 dex in initial metallicity, suggests choices between T6.0D400-3 and T6.2D400-3 to match the observations [133,135,152]. Interestingly, for the approximately 4 Gyr old, solar metallicity open cluster M67, predictions from stellar models with uninhibited diffusion are generally consistent with observed abundances [153], although the predicted abundance variations are small.

Two additional forms for *D*_turb_ have been proposed, but not widely applied, to comparisons with spectroscopic observations. The first one applied to low-mass star models with convective envelopes is

6.23 $$D_{\mathrm{turb}},VdB=f\frac{{\left(\rho_{\mathrm{BCZ}}/\rho \right)}^{3}}{{\left(1-(M_{\mathrm{BCZ}}/M_{\mathrm{tot}})\right)}^{n}},$$

with *f*=15 and *n*=1.5 [154], where BCZ denote quantities at the lower boundary of the convective envelope and *M*_tot_ is the total mass of the model. Owing to the steep power-law dependence of the density ratio, *D*_turb_ becomes negligible in the nuclear-burning regions, ensuring that the assumed turbulence will not affect the inner chemical profiles resulting from nucleosynthesis and gravitational settling.

The other proposed parametrization is proportional to the radiative viscosity [155]

6.24 $$D_{\mathrm{turb},\mathrm{visc}}=f\frac{4aT^{4}}{15c\kappa \rho^{2}},$$

where *f* is a free parameter that is constrained in the range $f=1_{-0.2}^{+2.0}$ by helioseismic observations, and spectroscopy of Hyades stars and OB stars (in this latter case assuming no competing effect from ML).

**Figure 20.**
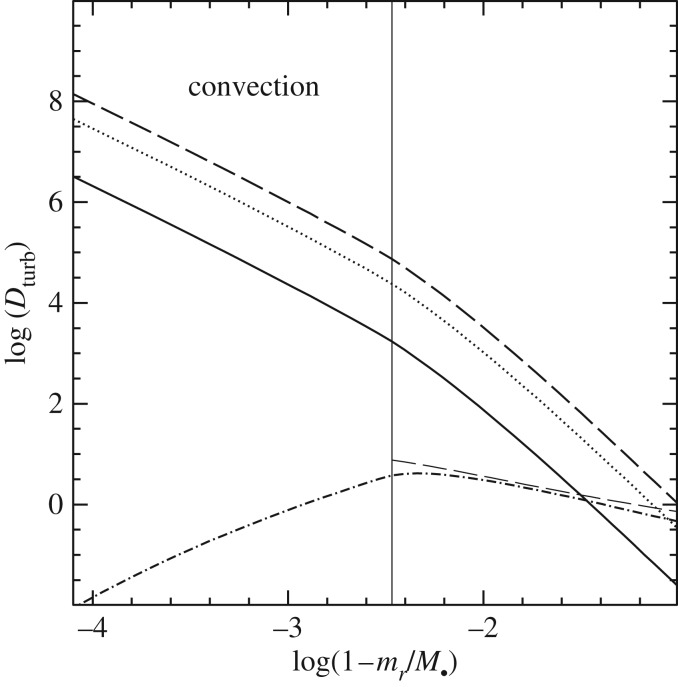
Comparison of *D*_turb,Rich_ solid line for the T6.0D400-3 choice of free parameters, dotted line for T6.2D400-3), *D*_turb,VdB_ (dashed line) and *D*_turb,visc_ (dotted-dashed line) as a function of the mass location within a 0.8*M*_⊙_, [Fe/H]= −1.3 model, in the latter phase of its MS evolution. The vertical thin line marks the bottom of the convective envelope, while the thin dashed line displays the diffusion coefficient of He (see text for details).

Figure 20 compares the different formulations for *D*_turb_ discussed above, for the outer layers of a 0.8*M*_⊙_, [Fe/H]=−1.3 model, in the latter phase of its MS evolution. In the case of *D*_turb,Rich_, we display both the T6.0D400-3 and T6.2D400-3 results, the ones that allow a match to results from spectroscopic observations, as discussed before. Notice how when moving the reference temperature from $\log(T_{0})=6.0$ to $\log(T_{0})=6.2$, the entire profile of *D*_turb,Rich_ shifts to deeper layers, thus counteracting the efficiency of diffusion in deeper stellar regions. Also, *D*_turb_,*V*d*B* (displayed with the choice of parameters recommended in [154]) appears to be very similar to *D*_turb,Rich_ for the T6.2D400-3 choice. On the other hand, both absolute values and trends of *D*_turb,visc_ (for *f*=1) with mass depth are very different from the other cases.

For the sake of comparison, we also show the diffusion coefficient of He (that is never supported by radiative levitation) at the edge of the convective envelope and below. All *D*_turb_ choices displayed in the figure are able to counteract the diffusion of He from the bottom of the convective zone. Even *D*_turb,visc_, that is the smallest turbulent diffusion coefficient in the figure, matches the one for He, hence is able to inhibit its settling below the convective envelope.

## 7. Phase separation in white dwarfs

Besides convection in the non-degenerate envelopes and the diffusion of ^22^Ne in the liquid core, another important process that redistributes the chemical abundances in cold WDs is the so-called ‘chemical separation’ upon crystallization [156]. In a nutshell, when a layer with given C and O abundances in the liquid phase crystallizes (we assume for the moment the core being made of just C and O, given that the sum C+O is always more than approximately 95% by mass in any realistic WD model), the equilibrium composition in the solid phase—given by the phase diagram of the CO mixture—is very likely to be different. This will cause a gradual change of the overall chemical profiles within the CO core that impacts the energy budget and cooling times, as detailed below.

**Figure 21.**
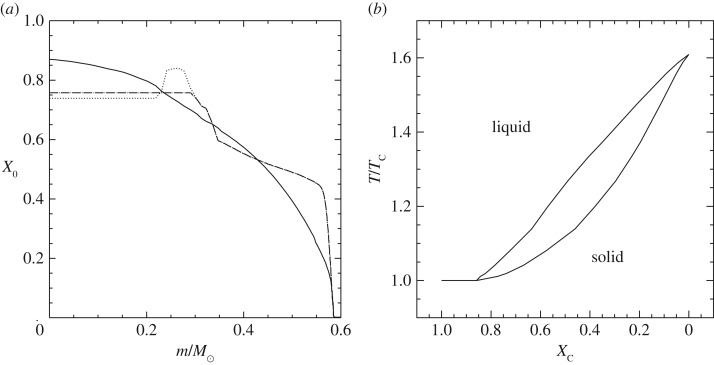
(*a*) Oxygen profile (in mass fraction) of a 0.61 *M*_⊙_ WD at the beginning of the thermal pulsing phase of the progenitor (dotted line), and the same after rehomogenization due to the *μ* inversion (dotted-dashed line) and after complete crystallization (solid line) [157]. (*b*) Phase diagram of the CO mixture adopted to calculate the profiles in the left panel [158]. *T*_C_ denotes the crystallization temperature of a pure C composition, *X*_C_ the C mass fraction.

Figure 21*b* displays a phase diagram for the CO mixture [158], with the regions of liquid and solid phase labelled. For heuristic purposes, let us assume a C mass fraction *X*_C_=0.50 uniformly throughout the core (hence the oxygen mass fraction *X*_O_ is also approx. 0.50). When the mixture starts to crystallize at the centre (the value of the Coulomb parameter *Γ*=180 for the onset of crystallization is reached first in the centre, because of higher densities), the chemical composition in the solid phase is determined as follows. One needs to draw a vertical line in the phase diagram of figure 21 with the horizontal coordinate equal to 0.50, which runs through the region belonging to the liquid phase until it intersects the upper line describing the phase diagram. The vertical coordinate of the intersection point gives the crystallization temperature of the WD centre (corresponding to *T*∼1.3*T*_C_, where *T*_C_ denotes the crystallization temperature of a pure C mixture). From this point, one has to draw a horizontal line that will intersect the lower segment of the phase diagram in correspondence with a carbon abundance *X*_C_∼0.30. This is the equilibrium abundance of carbon in the solid phase (the corresponding oxygen abundance is *X*_O_=1−*X*_C_).

Given that *X*_C_ in the now-crystallized centre is lower than the initial value, conservation of mass requires that the carbon abundance in the liquid phase at the crystallization boundary is increased (hence *X*_O_ is decreased) with respect to the original value. This means that right above the crystallized boundary the molecular weight is now lower than in the overlying layers still in the liquid phase (where the ratio *X*_O_/*X*_C_ is higher). An increase in molecular weight with increase in distance from the centre in the liquid phase causes an instability to develop, and the resulting fast-mixed region extends outwards in mass as long as the new uniform average *X*_C_ value is higher than the abundance in the next, unperturbed layer (in this case, it will reach the edge of the CO core) [159,160]. After mixing is completed, the liquid phase will have an overall enhanced *X*_C_ abundance compared to the value before the crystallization of the central layer.

Let us now suppose that the new value of *X*_C_ at the boundary of the solid core is equal to 0.55 when this layer crystallizes—at a lower temperature than the core because of lower density. The abundance in the solid phase can be derived in the same way as before, and it is now equal to *X*_C_∼0.35. This implies again that right outside the newly crystallized layer the abundance *X*_C_ must be higher (and *X*_O_ lower) than in the overlying layer. The instability in the liquid phase ensues again and the cycle is repeated (mixing in the liquid phase eventually stopping when the carbon abundance of the newly crystallized layer becomes lower than or equal to the overlying layers still in the liquid phase) until the whole degenerate core is crystallized. The final profile of *X*_C_ and *X*_O_ after crystallization is completed is no longer homogeneous; *X*_O_ will display central values higher than in the liquid phase which decrease from the centre outwards, while the opposite is true for *X*_C_.

This variation of the *X*_O_/*X*_C_ profile (hence the local value of the molecular weight *μ*) in the WD core during crystallization due to the CO phase diagram has an important impact on the WD cooling times, because of the term (∂*U*/∂*μ*)_*T*,*v*_(∂*μ*/∂*t*) in *ϵ*_g_ [157]. This means that more energy is available to be released and cooling times are longer. Depending on the thickness and chemical composition of the non-degenerate layers surrounding the CO core (that regulate the energy release), delays induced by chemical separation upon crystallization can reach approximately 10% for the coolest objects.

Figure 21*a* displays the evolution of the O-profile for a realistic 0.61*M*_⊙_ WD model [157]. The first stage of this temporal sequence is represented by the profile in the electron degenerate core at the beginning of the thermal pulse phase of the AGB progenitor, built during the core He-burning. One can notice the local maximum in *X*_O_ is about 0.25*M*_⊙_ away from the centre. The associated local maximum of the molecular weight *μ* causes an instability that homogenizes the internal layers during the liquid phase of the cooling [157], generating the profile that is maintained until the start of core crystallization. The final very different *X*_O_ profile is attained when the whole core is crystallized, and it is produced by the chemical separation upon crystallization.

**Figure 22.**
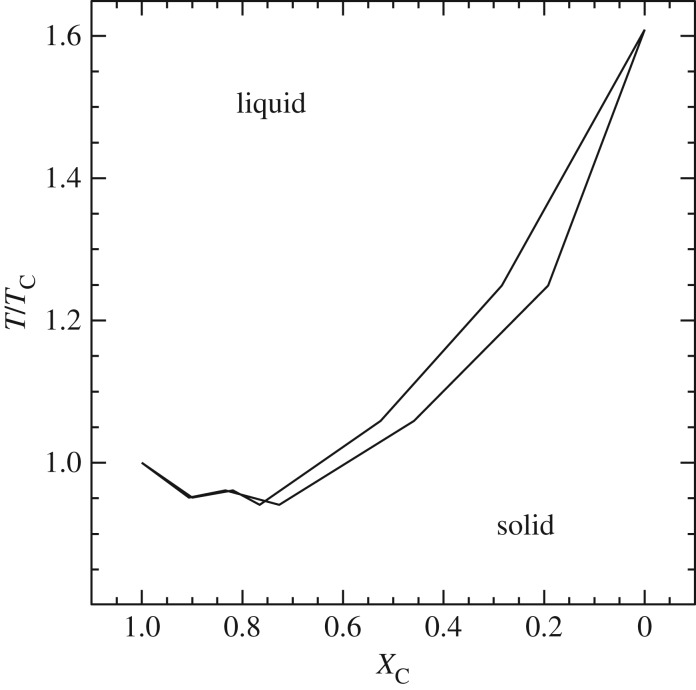
The most recent re-evaluation of the phase diagram for a CO mixture [161].

The most recent re-evaluation of the phase diagram for a binary CO mixture is displayed in figure 22 [161]. Compared to the widely used phase diagram of figure 21, it causes slightly smaller abundance variations for a given initial chemical profile in the liquid phase.

Within the CO core there are also small amounts of other metals—so-called minor species, with mass fractions of at most the order of the initial progenitor metallicity—that have been either processed through the previous burning phases (i.e. Ne), or are unchanged since the formation of the progenitor (i.e. Fe). Owing to the extreme complexity of calculating a multi-component phase diagram, a ternary CONe mixture is often assumed to behave as an effective binary mixture composed of neon (or iron) plus an element of average charge 〈*Z*〉=7 determined from the C and O abundances. Ne is important, given that ^22^Ne is the most abundant species after C and O, with a mass fraction *X*_^22^Ne_∼*Z*, where *Z* is the initial metallicity of the progenitor (*Z*∼0.02 for the solar metallicity). A detailed phase diagram for a three-component mixture CONe shows that the effect of Ne on the final WD cooling times is negligible, when the separation of carbon and oxygen is accounted for [162].

## 8. Rotation and rotational element transport mechanisms

Basic considerations about the angular momentum evolution in a contracting protostellar cloud, observations of the solar magnetic field and stellar spectroscopy (and now also asteroseismology) dictate/show that stars do rotate. Observations of MS O- and B-type stars in the Galaxy and the Magellanic Clouds reveal average projected rotational velocities of the order of 150 km s^−1^ [163–166], with values up to 350–400 km s^−1^. Average rotational velocities of the order of 150 km s^−1^ are observed also in MS A- and F-type stars [167], fast decreasing when moving to later spectral types [168], while giant stars display typically slow projected rotational velocities below approximately 10 km s^−1^ [169].

All fast rotators among O-stars show surface He-enrichments not predicted by standard non-rotating models [170]. Also, high rotational velocities along the MS are associated with enhancements of surface N that cannot be reproduced by non-rotating stellar models [171,172].

For a long time, rotation has not been an ingredient of standard stellar models, due to the increase in complexity and uncertainty—free parameters—related to the inclusion of rotation. Moreover, the basic principle that the explanation relying on the smallest number of hypotheses is the one to be preferred, coupled to the many successes of non-rotating stellar models, means that rotation has been generally considered only a second-order effect. It is, however, clear that it can have important effects on the structure and evolution of stars, mainly through its effect on the evolution of the chemical stratification.

### 8.1. One-dimensional modelling of rotating stars

The physical basis for the inclusion of rotation in detailed stellar evolution computations was laid down 50 years ago [173,174] and more recently hydrodynamical treatments of the problem have appeared [175–177]. Detailed stellar evolution calculations and comprehensive libraries of stellar models are, however, still (and for the foreseeable future) possible only with standard 1D modelling, and the basic assumptions to simulate the average mechanical and thermal distortions induced by rotation without basically altering the standard equations, are the following^19^ :

(i) Roche approximation, i.e. the gravitational potential *Ψ* is the same as if the total mass of the star were concentrated at the centre.
(ii) The angular velocity *Ω* and chemical composition are constant along isobars, e.g. surfaces of constant pressure (*shellular* rotation). This follows the assumption that turbulence is anisotropic, with a stronger transport in the horizontal (tangential to an isobar) direction than in the vertical (perpendicular to an isobar) one [178]. The angular velocity in this case depends very weakly on the colatitude, hence *Ω*≡*Ω*(*r*), meaning that it varies according only to the radial coordinate of the isobars.
(iii) The mean radius *r*_*P*_ of an isobar is defined by $V_{P}=(4\pi /3)r_{P}^{3}$, where *V*_*P*_ is the volume inside the isobar.

With these assumptions, the set of equations of stellar structure for a rotating star can be written in 1D as [179,180]

8.1 $$\begin{array}{rl} \frac{\partial r_{P}}{\partial m_{P}} & =\frac{1}{4\pi r_{P}^{2}\bar{\rho }},\\ \frac{\partial P}{\partial m_{P}} & =-\frac{Gm_{P}}{4\pi r_{P}^{4}}f_{p},\\ \frac{\partial L_{P}}{\partial m_{P}} & =\epsilon_{n}-\epsilon_{\nu }+\epsilon_{g}\\ \;\text{and}\;\;\frac{\partial \bar{T}}{\partial m_{P}} & =-\frac{Gm_{P}T}{4\pi r_{P}^{4}P}\nabla_{P} \end{array}\}$$

with ∇_*P*_ being the appropriate temperature gradient $d\;\ln(\bar{T})/d\;\ln(P)$. In the case of radiative transport,

8.2 $$\nabla_{P,\mathrm{rad}}=-\frac{3\kappa }{16\pi acG}\frac{P}{T^{4}}\frac{L_{P}}{m_{P}}\frac{f_{T}}{f_{p}}.$$

When comparing these equations to equations (2.1)–(2.4), one can notice that they are formally identical, apart from the *form factorsf*_*P*_ and *f*_*T*_. The only difference is the interpretation of the variables. In the case of rotating models, *r*_*P*_ is the mean radius of an isobar defined by the value *P* of the pressure, $\bar{\rho }$ and $\bar{T}$ are the volume-averaged density and temperature between two contiguous isobars (the difference with averages on the isobars is negligible if the mass grid of the models is dense enough), and *L*_*P*_ and *m*_*P*_ are the mass and luminosity inside a given isobar. Calculations of the energy generation coefficients, adiabatic gradient and opacity make use of $\bar{\rho }$ and $\bar{T}$. The equation of state is expressed in terms of $P=P(\bar{\rho },\bar{T})$ and the chemical composition on the isobar *P*.

Analogous to the case of non-rotating models the system of equations is solved considering the ‘Lagrangian’ independent variable *m*_*P*_, with $r_{P},L_{P},P,\bar{T}$ as unknowns.

The form factors *f*_*P*_ and *f*_*T*_ are defined, respectively, as

8.3 $$f_{P}=\frac{4\pi r_{P}^{4}}{Gm_{P}S_{P}}\frac{1}{\left\langle g^{-1}\right\rangle }$$

and

8.4 $$f_{T}={\left(\frac{4\pi r_{P}^{2}}{S_{P}}\right)}^{2}\frac{1}{\left\langle g\rangle \langle g^{-1}\right\rangle }.$$

The quantities 〈*g*〉 and 〈*g*^−1^〉 are average values of the gravity *g* over an isobar *P* with surface area *S*_*P*_. For a generic variable *f* this average is defined as

8.5 $$\langle f\rangle =\frac{1}{S_{P}}\int_{P=\mathrm{const}.}f\;d\sigma ,$$

where d*σ* is an infinitesimal element of the isobar surface *P*. At the non-rotation limit, *f*_*P*_ and *f*_*T*_ converge to unity and the equations are reduced to their non-rotating form.

To solve equations (8.1), one needs to evaluate *f*_*P*_ and *f*_*T*_, and this requires the calculation of the surfaces of isobars, e.g. surfaces where *Ψ*_*P*_ is constant, with

8.6 $$\Psi_{P}=-\Phi +\frac{1}{2}\Omega^{2}r^{2}\sin^{2}\theta ,$$

where *Φ* is the Roche gravitational potential, *r* is the radial distance from the centre and *θ* is the colatitude (*θ*=0 at the poles; see e.g. [181] for an example of how to calculate integrals (8.5) for a given *Ψ*_*P*_). It is through the calculation of *f*_*P*_ and *f*_*T*_ that the angular velocity profile enters the equation of stellar evolution. It is worth noticing that all this formalism works also in the case of a ‘conservative’ potential (for example, the simple case of solid body rotation), e.g. if the centrifugal acceleration can be derived from a potential [182,183], which is not the case for shellular rotation. A potential of this type is used, for example, in [182]

8.7 $$\Psi =\Phi -\frac{1}{2}\Omega^{2}r^{2}\sin^{2}\theta .$$

In this case, instead of isobaric surfaces one reads equipotential surfaces, and the equations (including *f*_*P*_ and *f*_*T*_) are the same as the shellular case.

### 8.2. Chemical element and angular momentum transport

A tricky issue for modelling rotating stars with 1D stellar models is how to describe the transport of angular momentum—that determines the evolution of *Ω*—and the chemical mixing associated with rotation. These are two facets of the same problem, for rotation triggers hydrodynamical instabilities and large-scale motions of the gas in radiative regions, which result in transport of both angular momentum and chemical elements. In-depth discussions about rotation-driven instabilities can be found in [180,184,185]. Here, we just give a brief overview, focussing on the actual implementation in stellar evolution calculations.

The Eddington–Sweet meridional circulation is one of the major instabilities caused by rotation. In simple terms, we can compare a non-rotating star in radiative equilibrium with its solid body rotating counterpart. The equipotential surfaces of the non-rotating star are spherical, while in the case of a solid body rotator they are rotational ellipsoids, and two contiguous equipotential surfaces will diverge in distance from each other at the equator. Given that the effective gravity *g* is proportional to the gradient of the potential (that is normal to the equipotential surfaces), it will vary with latitude on an equipotential surface. As a consequence, the temperature will be hotter at the poles and cooler at the equator—as demonstrated by von Zeipel almost a century ago, the energy flux is proportional to the local value of *g* (the von Zeipel theorem)—preventing the star from maintaining hydrostatic equilibrium.

The solution to this paradox is to invoke large-scale mass motions that transport energy, the so-called *meridional circulation*, moving material inwards from the equator and upwards along the rotational axis towards the poles. The timescale for this mixing process—the ‘Eddington–Sweet’ timescale—was estimated to be

8.8 $$t_{\mathrm{ES}}\approx \frac{GM^{2}}{LR}\frac{GM}{\Omega^{2}R^{3}},$$

where *L*, *M* and *R* are the stellar luminosity, mass and radius, respectively, *Ω* the angular velocity and the first term is the Kelvin–Helmholtz thermal timescale. These early estimates of *t*_ES_ were much shorter than the MS lifetime of stars, even for modest rotation rates, hence rotating stars should be fully mixed, contradicting observational data. The presence of chemical abundance stratifications (*μ*-gradients) in the interior of rotating stars can, however, increase *t*_ES_ considerably compared to the early estimates. A widely used model of the meridional circulation included in stellar evolution codes (that will be used later when discussing the implementation of rotational mixing) develops the circulation velocity vector **U** into two components [178]:

8.9 $$\text{U}=U_{2}(r)P_{2}(\cos(\theta )){\text{e}}_{r}+V_{2}(r)\frac{dP_{2}(\theta )}{d\theta }{\text{e}}_{\theta },$$

where *r* is the radial coordinate, *θ* is the colatitude and *P*_2_ is the Legendre polynomial of order 2. The radial components of these velocities, which will be used to treat chemical element transport, are related through

8.10 $$\frac{1}{r}\frac{d}{dr}[\rho r^{2}U_{2}(r)]-6\rho V_{2}(r)=0.$$

Another important effect of meridional circulation is that it advects also angular momentum. Local variations of angular velocity with time due to this angular momentum transport, plus the effect of contraction and expansion of the stellar layers (and eventually angular momentum loss from mass loss) will generate—starting, for example, from solid body rotation, usually assumed for pre-MS fully convective stars—a variation of angular rotation velocity with depth in radiative regions. Hence a ‘shear’ develops between neighbouring layers that leads to instabilities. This stems from the fact that the minimum energy state of a rotating fluid is solid body rotation, and if the star develops differential rotation, it is possible to extract energy by homogenizing the velocities through transport of material.

The strong thermal and molecular weight radial stratification in radiative zones tends to oppose the homogenization of the rotational velocities, while it is reasonable to assume that no restoring forces oppose horizontal displacements. As a consequence, horizontal shear (along an isobar) is expected to generate a strong turbulence on short dynamical timescales, justifying the assumption of shellular rotation. Eventually, thermal diffusion and horizontal shear can reduce the stabilizing effect of the vertical (radial) thermal and chemical stratification and induce element and angular momentum transport if the Richardson number *Ri* satisfies this criterion:

8.11 $$Ri\equiv \frac{N^{2}}{{\left(du/dz\right)}^{2}}<Ri_{\mathrm{crit}}=\frac{1}{4},$$

where *u* is the velocity of the fluid elements and *z* designates the vertical direction.^20^

The Solberg–Høiland instability, plus additional instabilities (i.e. Goldreich–Schubert–Fricke, ABCD instabilities) that can develop when equipotentials and isobars do not coincide—so-called baroclinic instabilities [180]—do potentially contribute to the transport of angular momentum and chemicals.^21^ Some of these instabilities are implemented in calculations of rotating stellar models (see §8.2.2), even though their treatment is considered to be much more uncertain than the case of meridional circulation and shear instabilities [184,185].

In the case of radiatively driven winds, the von Zeipel result has important consequences on the mass loss rates to be employed in the calculation of rotating stellar models, because in general it causes an increase in the mass loss efficiency compared to the case of a non-rotating counterpart. Various prescriptions of the mass loss enhancement due to rotation can be found in the literature. As an example, we report the prescription employed in the MESA code [58]:

8.12 $$\frac{dM}{dt}(\Omega )=\frac{dM}{dt}(\Omega =0){\left(\frac{1}{\left(1-\Omega /\Omega_{\mathrm{crit}}\right)}\right)}^{\zeta },$$

with *ζ*=0.43, and *Ω*^2^_crit_=(1−*L*/*L*_Edd_)*GM*/*R*^3^ and *L*_Edd_=4*πcGM*/*κ* averaged over a certain optical depth range.

#### 8.2.1. Advective/diffusive implementation

Meridional circulation and shear instability are considered as the main mechanisms for angular momentum transport and rotational mixing in a number of stellar evolution codes, e.g. STAREVOL [187,188], CESTAM [189], the Geneva stellar evolution codes [179,190] and the FRANEC code [191], that implement advective+diffusive transport of angular momentum and diffusive rotational chemical mixing.

The transport equation for a chemical element with mass fraction *X*_*i*_ is written as^22^

8.13 $${\frac{\partial X_{i}}{\partial t}|}_{M_{r}}=\frac{1}{\rho r^{2}}\frac{\partial }{\partial r}\left(\rho r^{2}D_{\mathrm{chem}}\frac{\partial X_{i}}{\partial r}\right),$$

where *D*_chem_ is the sum of the vertical shear diffusion coefficient *D*_shear_ and the effective diffusion coefficient *D*_eff_, which accounts for the combined effect of the strong horizontal shear diffusion *D*_h_ and of the meridional currents:

8.14 $$D_{\mathrm{eff}}=\frac{1}{30}\frac{|rU_{2}^{2}(r){|}^{2}}{D_{h}}.$$

The equation for the transport of angular momentum is written as [192]

8.15 $${\frac{\partial }{\partial t}(r^{2}\Omega )|}_{M_{r}}=\frac{1}{5\rho r^{2}}\frac{\partial }{\partial r}(\rho r^{4}\Omega U_{2}(r))+\frac{1}{\rho r^{2}}\frac{\partial }{\partial r}\left(\rho D_{\mathrm{ang}}r^{4}\frac{\partial \Omega }{\partial r}\right),$$

where *D*_ang_ denotes the diffusion coefficient for angular momentum. The second term on the right-hand side of equation (8.15) is a diffusion term, similar in its form to equation (8.13), while the first term is an advective term, modelling the transport by a velocity current. Equation (8.13) does not contain an advective term, following the author of [193] who showed that the combined effect of turbulence and circulation currents is equivalent to a diffusion process for the element transport.

There are various expressions in the literature for *U*_2_. The complete description by Maeder & Zahn [194] in the case of shellular rotation provides

8.16 $$U_{2}(r)=\frac{P}{\bar{\rho }\bar{g}C_{P}\bar{T}}\frac{1}{\left(\nabla_{\mathrm{ad}}-\nabla +(\phi /\delta )\nabla_{\mu }\right)}\left[\frac{L}{M}(E_{\Omega }+E_{\mu })+\frac{\bar{T}C_{P}}{\delta }\frac{\partial \Theta }{\partial t}\right].$$

The barred symbols indicate averages over the isobar; *E*_*Ω*_ and *E*_*μ*_ are complicated terms that depend on the angular velocity profile and mean molecular weight fluctuations on an isobar (see [180] for the complete formulae and the derivation); *Θ* describes the density fluctuation on an isobar; and *M* here denotes the reduced mass

8.17 $$M=M_{r_{P}}\left(1-\frac{\Omega^{2}}{2\pi G\rho_{m}}\right),$$

where *ρ*_m_ is the mean density inside the considered isobar surface, and *L* is the luminosity within the same surface.

In the case of convective regions, the transport of angular momentum is very uncertain, as the interaction between convection and meridional currents is not well understood. Traditionally, one can choose between the following two limiting cases. If convection inhibits meridional currents, angular momentum is redistributed very efficiently by convection, as in the case of chemical elements, and the result will be solid body rotation, as in the solar convective envelope. If meridional currents dominate—one can hypothesize that this is the case applicable to large, rarefied RGB envelopes, where convective elements may collide elastically rather than inelastically as in the solid body case—it is the specific angular momentum that is expected to be uniform. Hydrodynamical simulations have shown that the extended deep convective envelopes of red giant stars are likely to undergo radial differential rotation, with an angular velocity profile of the form *Ω*(*r*)∝*r*^−0.5^ [195]. In general, solid body rotation is usually prescribed in convective regions.

Angular momentum losses due to stellar winds need also to be accounted for. In the assumption that the mass loss is spherically symmetric, the rate of angular momentum loss during a given time step will be approximately equal to the average specific angular momentum at the surface of the star multiplied by the assumed mass loss rate.

**Figure 23.**
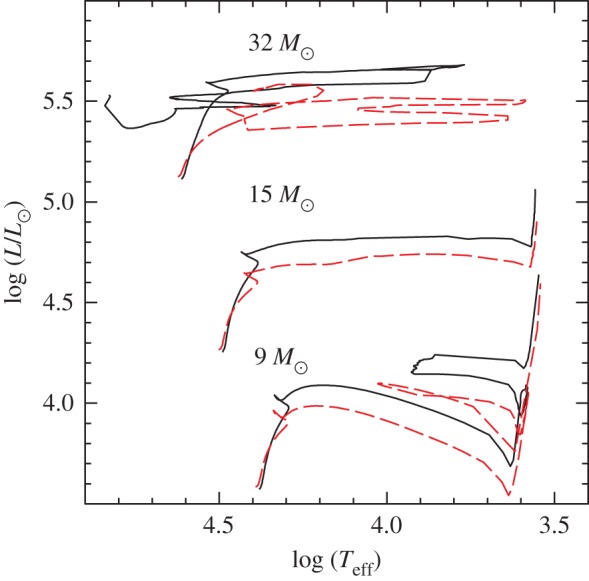
Comparison of the HRD of 9, 15 and 32*M*_⊙_ solar initial composition models, with (solid line) and without (dashed line) rotation, from the Geneva calculations [196].

To discuss the effect of rotation and rotational mixing on stellar evolution models, we consider as an example models from [196], calculated with the Geneva code. Figure 23 compares the HRD of 9, 15 and 32*M*_⊙_ solar initial chemical composition models with and without rotation, from the zero age main sequence (ZAMS) to the end of core C-burning. In these calculations, *D*_h_ has been taken as

8.18 $$D_{h}=\frac{1}{c_{h}}r|2V_{2}(r)-\alpha U_{2}(r)|,$$

from [178], assuming *c*_h_=1 and $\alpha =\frac{1}{2}(\text{d}\ln(r^{2}\Omega )/\text{d}\ln r)$. We express *D*_shear_ as

8.19 $$D_{\mathrm{shear}}=f_{\mathrm{energ}}\frac{H_{P}}{g\delta }\frac{K_{T}}{\left[(\varphi /\delta )\nabla_{\mu }+(\nabla_{\mathrm{ad}}-\nabla_{\mathrm{rad}})\right]}{\left(\frac{9\pi }{32}\Omega \frac{\text{d}\ln\Omega }{\text{d}\ln r}\right)}^{2}$$

from [197], where *f*_energ_=1 (the fraction of the excess energy in the shear that contributes to mixing).

The models are initialized as solid bodies at the ZAMS, and then evolved according to the prescriptions for the angular momentum and chemical transport described above. The prescribed initial equatorial rotation velocity is 0.4 times the critical velocity (when the centrifugal acceleration in the equatorial plane exactly compensates the gravitational acceleration) of the corresponding stellar mass. This is equal to values between approximately 250 and 300 km s^−1^ for the three displayed masses.

The effect of rotation on the tracks (that of course depends on initial velocities) is striking. Models calculated with rotation evolve to higher luminosities during the MS and stay more luminous also during the following evolutionary phases. This is mainly due to the consequences of the chemical element transport associated with rotation. In general, rotational mixing works in the direction of erasing chemical gradients within the star, and hence during the MS, this causes a continuous slow ingestion of fresh hydrogen into the H-depleted convective core, as well as a slow transport towards the surface of elements whose abundances are increased by H-burning (e.g. He and ^14^N). The effect of the ingestion of H in the convective core is to increase the MS lifetime compared to the non-rotating counterpart, and to produce slightly larger He-cores at the end of core H-burning.

**Figure 24.**
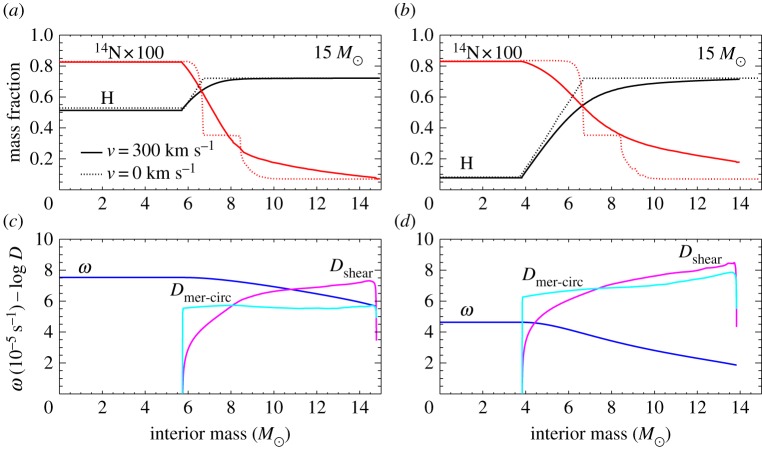
Internal profiles of H and N abundances, angular velocity and chemical diffusion coefficients for shear and meridional circulation, respectively, as a function of mass, within 15*M*_⊙_ solar initial composition models from [191], with the labelled initial rotation rates. The different panels refer to two MS stages, when the central H mass fraction is equal to 50% (*a*,*c*) and 10% (*b*,*d*), respectively (courtesy of M. Limongi).

To this purpose, figure 24 displays the internal profiles (taken during the MS when central H is reduced to—from (*a*,*c*) to (*b*,*d*)—50% and 10% by mass, respectively) of H and N abundances, angular velocity and chemical diffusion coefficients for shear and meridional circulation in solar initial composition, 15*M*_⊙_ rotating and non-rotating evolutionary models from [191], similar to the Geneva calculations. One can see very clearly the effect of rotational mixing, which smooths out gradients in the N- and H-abundance profiles. Also, shear mixing dominates in the external layers, where the gradient of angular velocity gets progressively steeper, while meridional circulation is the more efficient chemical transport process close to the edge of the convective core. Notice also the flat angular velocity profile in the convective core, due to the solid body assumption.

The different paths in the HRD followed by rotating and non-rotating models together with the different lifetimes lead also to different mass loss histories and hence to different total masses at the end of the MS and during the post-MS phases. This impacts, for example (for a fixed convective mixing scheme), the occurrence of ‘loops’ in the HRD, as the blue or red location of a model during the giant phase depends on thickness of the H-rich envelope (thick enough envelopes keep the models at the red side of the HR, while when their masses decrease below a threshold value, the models move to the blue). During the He-burning phase, the slow ingestion of fresh He in the core lowers the final C/O ratio and increases the mass of the CO core [191], affecting the pre-supernova structure of the massive models.

**Figure 25.**
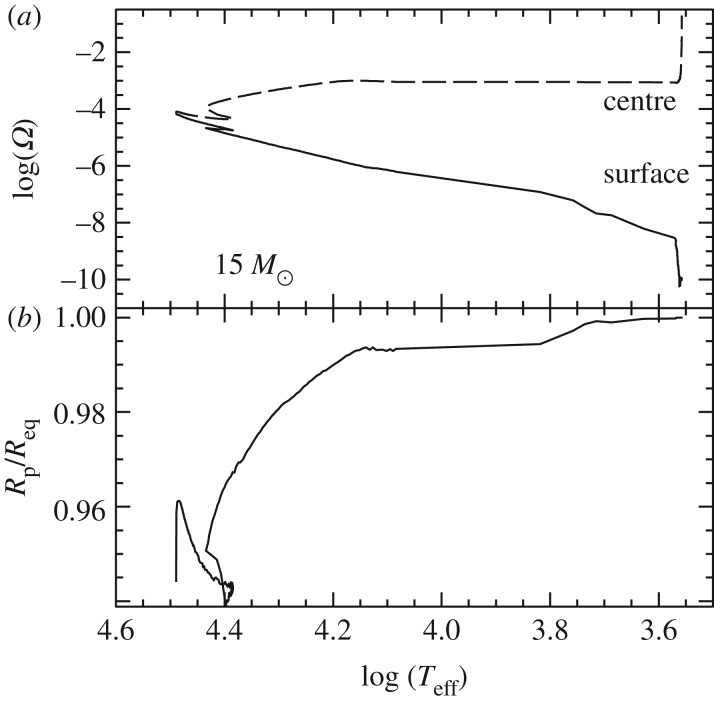
(*a*) Displaying the evolution of the surface and central angular velocity as a function of *T*_eff_ for the 15*M*_⊙_ models in figure 23. (*b*) Displaying the evolution of the ratio of the polar and equatorial radius.

Figure 25*a* shows the evolution of the angular velocity *Ω* taken at the surface and at the centre of the 15*M*_⊙_ models. After the ZAMS model with enforced solid body rotation is left to evolve, there is a readjustment of the rotational profile at the very beginning of the evolution, until the equilibrium rotational profile is reached, and the angular velocity starts to evolve under the action of the transport mechanisms. The general trend is to transfer angular momentum from the contracting core towards the external layers, but this is counterbalanced by the mass loss that removes angular momentum from the surface and eventual expansions of the convective envelopes that slow down the surface.

For the model shown in figure 25, the net effect is a constant decrease of the surface angular velocity along the whole evolution, whereas the centre of the star displays a moderate increase of angular velocity during the MS, followed by a plateau and a sharp increase during the giant phase. The models are never very far from spherical symmetry, as shown in figure 25*b*, which displays the ratio of the polar to the equatorial radius along the whole evolution.

**Figure 26.**
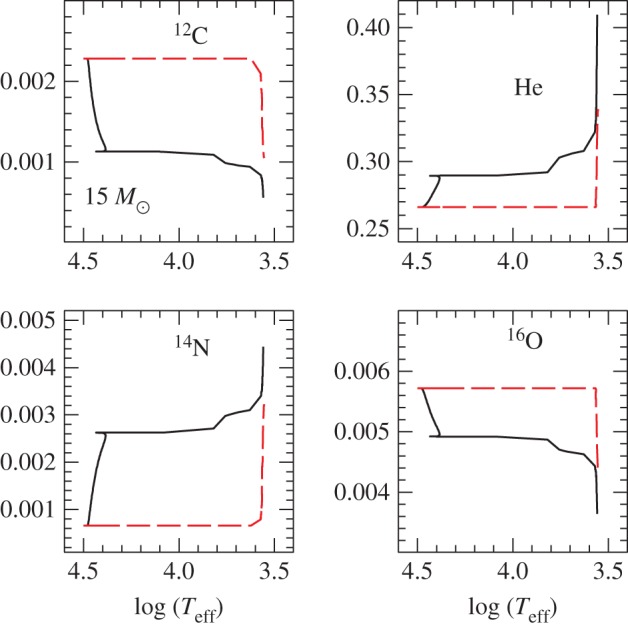
Evolution with *T*_eff_ of the surface abundances (in mass fractions) of the labelled elements, for the rotating (solid lines) and non-rotating (dashed lines) models in figure 23.

The effect of element transport on the surface abundances (in mass fractions) of some key elements (He, C, N, O) for the same 15*M*_⊙_ evolution is displayed in figure 26. Notice that in the non-rotating models the abundances change only due to the dredge-up during the red giant phase. Rotating models display instead abundance changes already during the MS, due to rotational mixing that tends to erase chemical gradients. This explains the increase of ^14^N and He, whose abundance increase in the central regions due to CNO H-burning, and the depletion of ^12^C and ^16^N, caused by the decreased O and N equilibrium abundances, compared to the initial scaled solar values. The abundances remain almost constant during the fast transition to the giant phase, and then change again due to the dredge-up.

The detailed behaviour of the evolution of the angular momentum and chemical abundance profiles is, however, strongly dependent on the precise choice of the diffusion coefficients in equations (8.13) and (8.15). We can see, for example, that the coefficient employed in these calculations contain parameters like *c*_h_ and *f*_energ_ that are set to fixed constant values not derived from first principles.

**Figure 27.**
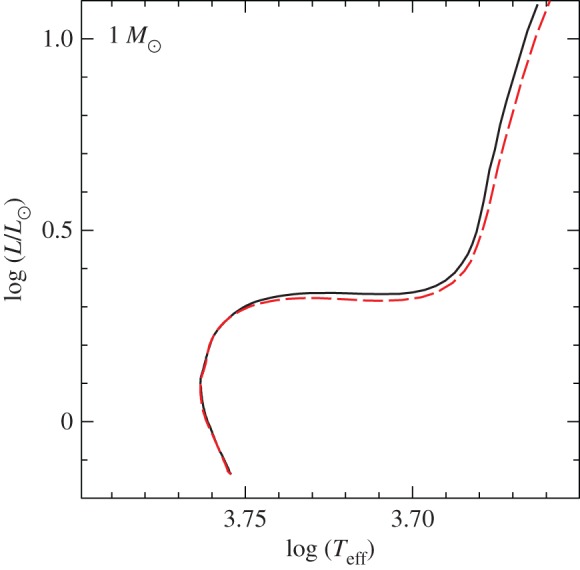
As figure 23 but for 1*M*_⊙_ models.

It is extremely interesting to analyse also the case of the 1*M*_⊙_ rotating (ZAMS equatorial velocity equal to 50 km s^−1^) and non-rotating models for the same initial chemical composition. Both calculations account also for atomic diffusion, but without including radiative levitation, whose effect is practically negligible at this metallicity. Figure 27 compares their HRDs,^23^ which are virtually identical along the MS, while the rotating SGB is slightly brighter and the following RGB phase slightly hotter. The MS lifetime of the rotating model is just approximately 4% longer than the non-rotating case.

**Figure 28.**
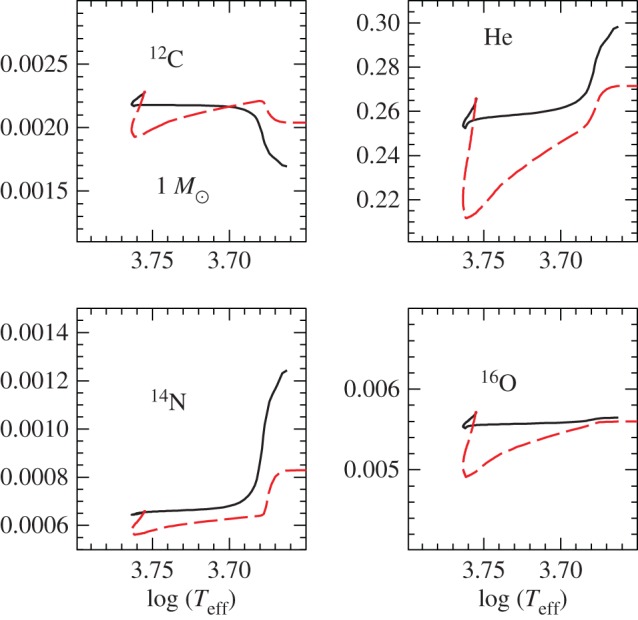
As figure 26 but for 1*M*_⊙_ models.

Figure 28 compares the evolution with *T*_eff_ of the surface abundances of key elements affected by rotational mixing. In the case of non-rotating models, the signature of efficient diffusion during the MS is very clear (see §6). The abundances of all elements decrease, reaching a minimum around the TO, and then increase when convection deepens, before the signature of the first dredge-up can be seen for C, N and He. When rotation is included, the abundances stay constant along the MS, almost equal to the initial values, showing that rotational mixing (for the chosen initial rotational velocity at this metallicity) strongly inhibits the effect of diffusion from the convective envelopes. Along the RGB of the rotating models displays stronger enhancements of N and He, and a larger depletion of C compared to the non-rotating counterparts.

There are also alternative expressions for *D*_shear_ and *D*_h_, in addition to the ones employed in the calculations we are discussing, namely:

*D*_shear_ from [198]
  8.20 $$D_{\mathrm{shear}}=f_{\mathrm{energ}}\frac{H_{P}}{g\delta }\frac{\left(K_{T}+D_{h}\right)}{\left[(\varphi /\delta )\nabla_{\mu }(1+K/D_{h})+(\nabla_{\mathrm{ad}}-\nabla_{\mathrm{rad}})\right]}{\left(\frac{9\pi }{32}\Omega \frac{\text{d}\ln\Omega }{\text{d}\ln r}\right)}^{2},$$
  with *K*, *f*_energ_ and *φ* as in [197].
*D*_h_ from [199]
  8.21 $$D_{h}=Ar{\left(r\Omega V_{2}(r)|2V_{2}(r)-\alpha U_{2}(r)|\right)}^{1/3},$$
  with *α* as in [178] and *A*=0.002.
*D*_h_ from [200]
  8.22 $$D_{h}={\left(\frac{\beta }{10}\right)}^{1/2}{\left(r^{2}\Omega \right)}^{1/2}{\left(r|2V_{2}(r)-\alpha U(r)|\right)}^{1/2},$$
  with *α* as in [178] and *β*=1.5×10^−6^.

The effect of using these various combinations of *D*_shear_ and *D*_h_ in the Geneva code has been investigated recently [201].^24^ They found that MS lifetimes, the evolution of surface velocities and the angular momentum of the core have a weak dependence on the choice of these diffusion coefficients. The shape of the evolutionary tracks, the surface enrichment (for a fixed initial rotation velocity), the blue-to-red evolution in the HRD and the extension of the blue loops are, however, significantly affected by the choice of *D*_shear_ and *D*_h_.

#### 8.2.2. Diffusive implementation

There is an alternative approach to include transport of chemicals and angular momentum, used in codes like the Yale evolutionary code [203], KEPLER, STERN and MESA. In this case, the temporal evolution of angular momentum and chemical abundances due to rotation is described by a set of two diffusion equations, computationally easy to implement:

8.23 $${\frac{\partial \Omega }{\partial t}|}_{M_{r}}=\frac{1}{\rho r^{4}}\frac{\partial }{\partial r}\left(\rho r^{4}\nu \frac{\partial \Omega }{\partial r}\right)$$

and

8.24 $${\frac{\partial X_{i}}{\partial t}|}_{M_{r}}=\frac{1}{\rho r^{2}}\frac{\partial }{\partial r}\left(\rho r^{2}D\frac{\partial X_{i}}{\partial r}\right),$$

where *ν* and *D* are, respectively, the total turbulent viscosity and the total diffusion coefficient defined as a sum of all diffusion coefficients associated with all the transport processes taken into account. Each of these diffusion coefficients is built as the product of the velocity *v* and the path length *l* of the redistribution currents with

8.25 $$l=\min\left(r,\left|\frac{\partial r}{\partial \ln v}\right|\right).$$

By |∂r/∂ln⁡v| is denoted the velocity scale height. The timescale associated with the *redistribution* over the path scale is simply *l*^2^/*D*=*l*/*v*.

This description is usually employed to include within the same formalism also convection and semiconvection, treated as diffusive processes. Appropriate diffusion coefficients for convective and rotational transports are adopted, often with free parameters to be calibrated against some sets of observations, given that these coefficients arise from order-of-magnitude considerations. Rotational mixing processes include at least meridional circulation, and shear, plus eventually additional rotational instabilities [58,76] not usually included in the codes that employ the advective/diffusive implementation.

The viscosity *ν* is prescribed by

8.26 $$\nu =D_{\mathrm{conv}}+D_{\mathrm{semiconv}}+\sum_{i,\mathrm{rotinst}}D_{i}$$

and the diffusion coefficient is usually written as the following sum [76]:

8.27 $$D=D_{\mathrm{conv}}+D_{\mathrm{semiconv}}+f_{c}\times \left(\sum_{i,\mathrm{rotinst}}D_{i}\right),$$

where *f*_c_ is one of the efficiency parameters entering the diffusive formalism and it is calibrated on observations like the solar lithium abundance (*f*_c_=0.046 in the models [203]), or the main trend of the observed nitrogen surface abundances with the projected rotational velocity for the nitrogen-enriched fast rotators in the LMC (*f*_c_=0.0228 in the models [204]).

An example of these implementations is given in the following, for the meridional circulation [76,203]. The characteristic velocity of these currents in a radiative region is assumed to be

8.28 $$v_{{\mathrm{ES}}_{0}}=\frac{\nabla_{\mathrm{ad}}}{\left(\delta (\nabla_{\mathrm{ad}}-\nabla \right)}\frac{\Omega^{2}r^{3}L}{{\left(GM\right)}^{2}}\left(\frac{2\epsilon r^{2}}{L}-\frac{2r^{2}}{M}-\frac{3}{4\pi \rho r}\right),$$

with *ϵ* denoting the energy generation rate per unit mass.

The counteracting effect of *μ*-gradients is accounted for according to

8.29 $$v_{\mu }=f_{\mu }\frac{\psi H_{P}}{\delta (\nabla_{\mathrm{ad}}-\nabla )\tau_{\mathrm{KH}}}\frac{|\nabla_{\mu }|}{\mu },$$

$\psi ={\left(d\;\ln(\rho )/d\;\ln(\mu )\right)}_{P,T},\tau_{\mathrm{KH}}$ is the local thermal timescale and *f*_*μ*_ is a free parameter that allows to vary the sensitivity of meridional circulation to *μ* gradients.

The effective value of the meridional circulation velocity used to determine the associated diffusion coefficient is finally calculated as *v*_ES_=|*v*_ES_0__|−|*v*_*μ*_| if *v*_ES_0__>*v*_*μ*_, or *v*_ES_=0 otherwise.

**Figure 29.**
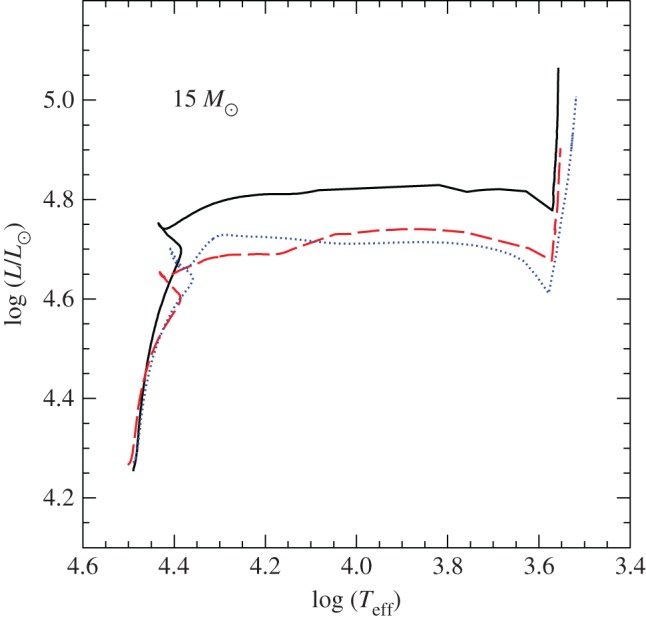
Comparison of the HRD of the Geneva models in figure 23 (same line styles) with the rotating counterpart (dotted line) from MESA calculations [58].

**Figure 30.**
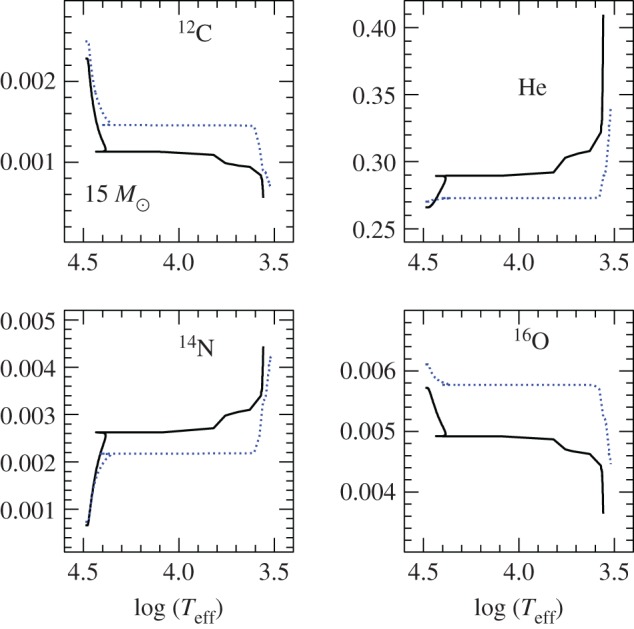
Comparison of the evolution of the surface abundances (in mass fractions) as a function of *T*_eff_ of the labelled elements, for the two sets of rotating models in figure 29. Solid lines refer to the Geneva calculations, dotted lines to the MESA models.

Figures 29 and 30 compare results for the initial solar composition 15*M*_⊙_ models discussed in figures 23, 25 and 26, with results of a MESA calculation (that employs this diffusive implementation for the transport of angular momentum and chemicals) for the same mass, solar initial chemical composition and approximately the same initial rotational velocity [205]. The HRD shows that these rotating models are almost equivalent to the non-rotating Geneva calculations. However, the evolution of the surface chemical elements shown in figure 30 displays clear signatures of rotational mixing, although the quantitative effect is very different from the Geneva results. The enhancement of the surface abundances during the MS is more moderate in the MESA calculations (even accounting for the slightly different initial abundances of some elements), and He is almost unchanged. Even the effect of the dredge-up seems to differ between the calculations.

It is difficult to determine the exact cause of these differences, given that also other elements of the model input physics (apart from the implementation of rotational transports) differ between the two sets of calculations. Very importantly, MESA calculations include also the effect of atomic diffusion (and radiative levitation), however, moderated by some additional turbulence [205]. But taken at face value, this comparison may give an idea of the uncertainties in the outputs of the current generation of rotating models.

### 8.3. Additional effects

There are at least two additional processes that may affect substantially the evolution of rotating stars, for they modify the transport of angular momentum and, directly or indirectly, also the transport of chemical elements. Their implementation in stellar model calculations is still uncertain and they are not generally included in the current generation of stellar models.

#### 8.3.1. Gravity waves

Inside a star, the density changes monotonically with the depth and the molecular weight typically increases in the direction of increasing gravity. When a gas element in this stable stratification is perturbed, the competition between buoyancy and gravity gives rise to an oscillatory motion around the equilibrium position and creates a so-called ‘internal gravity wave’ (IGW).

These IGWs are expected to be generated by the injection of kinetic energy from a turbulent region into an adjacent stable region, as observed both in two-dimensional (2D) and 3D simulations of convective mixing, e.g. by convective overshooting in a stable region and bulk excitation or excitation by Reynolds stresses inside the convection zone [206–208]. The IGWs penetrate into the radiation zone, transporting angular momentum that is deposited where they are dissipated through heat diffusion by photon exchange, which produces an ‘attenuation factor’ (*τ*) proportional to the thermal diffusivity and inversely proportional to the IGW frequency *ν* and amplitude. It is by shaping the internal rotation profile that IGWs contribute indirectly to the mixing of chemical elements.

Angular momentum transport by gravity waves is seldom included in evolutionary stellar model calculations. The current approximate treatment implemented in some stellar evolution calculations [184] expresses the solution of the equations describing the propagation of IGWs in a rotating star in terms of Legendre polynomials. At each point within a radiative region, the total angular momentum ‘luminosity’^25^ associated with the IGW propagation can be written as

8.30 $$L_{J}(r)=\sum_{\mathrm{waves}}L_{J,\nu ,\ell ,m}(r_{c})\exp[-\tau (r,\sigma ,\ell )],$$

where *r*_c_ denotes the radiation/convection interface, ℓ and *m* represent, respectively, the spherical order and the azimuthal number of the Legendre polynomial, *ν* the frequency of the IGW when launched from the convective zone, *σ*=*ν*−*m*(*Ω*(*r*)−*Ω*(*c*)) is the local wave frequency measured in the co-rotating frame with angular velocity *Ω*(*r*)—*Ω*(*c*) being the angular velocity of the solid body rotating convective zone—and

8.31 $$L_{J,\nu ,\ell ,m}=4\pi r^{2}F_{J}(\nu ,\ell ,m)=4\pi r^{2}\frac{2m}{\nu }F_{E}(\ell ,\omega ).$$

Here, ℱ_*J*_(*ν*,ℓ,*m*) is the mean flux of angular momentum carried by a monochromatic wave with emission frequency *ν*. The amplitude of the waves is assumed to be [209]

8.32 $$\begin{array}{rl} F_{E}(\nu ,\ell ) & =\frac{\nu^{2}}{4\pi }\int dr\frac{\rho^{2}}{r^{2}}\left[{\left(\frac{\partial \xi_{r}}{\partial r}\right)}^{2}+\ell (\ell +1){\left(\frac{\partial \xi_{h}}{\partial r}\right)}^{2}\right]\\ & \;\times \exp\left[\frac{-h_{\nu }^{2}\ell (\ell +1)}{2r^{2}}\right]\frac{v_{c}^{3}\Lambda^{4}}{1+{\left(\nu \tau_{\Lambda }\right)}^{15/2}} \end{array}$$

with *ξ*_*r*_ and [ℓ(ℓ+1)]^1/2^*ξ*_h_ being, respectively, the vertical and horizontal displacement wave functions normalized to unit energy flux at the edge of the considered convection zone, *v*_c_ the convective velocity, *Λ*=*α*_MLT_*H*_*P*_ the radial size of a convective element, *τ*_*Λ*_∼*Λ*/*v*_c_ the convective timescale and $h_{\nu }=\lambda \min\{1,{\left(2\nu \tau_{\Lambda }\right)}^{-3/2}\}$. The radial (*k*_*r*_) and horizontal (*k*_h_) wave numbers are related by

8.33 $$k_{r}^{2}=\left(\frac{N^{2}}{\omega^{2}}-1\right)k_{h}^{2}=\left(\frac{N^{2}}{\omega^{2}}-1\right)\frac{\ell (\ell +1)}{r^{2}}.$$

The local damping rate *τ*(*r*,*σ*,ℓ) can be written as

8.34 $$\tau (r,\sigma ,\ell )={\left[\ell (\ell +1)\right]}^{3/2}\int_{r}^{r_{c}}(K_{T}+\nu_{v})\frac{NN_{T}^{2}}{\sigma^{4}}{\left(\frac{N^{2}}{N^{2}-\sigma^{2}}\right)}^{1/2}\frac{dr}{r^{3}},$$

where $N_{T}^{2}$ is the thermal part of the Brunt–Väisälä frequency and *ν*_*v*_ is the vertical shear turbulent viscosity

8.35 $$\nu_{v}=\frac{8}{5}\frac{Ri_{\mathrm{crit}}{\left(r(d\Omega /dr)\right)}^{2}}{N_{T}^{2}/(K_{T}+\nu_{h})+N_{\mu }^{2}/\nu_{h}},$$

with $N_{\mu }^{2}$ denoting the molecular weight stratification part of the Brunt–Väisälä frequency, and *ν*_h_ the horizontal shear turbulent viscosity, which can be set to *D*_h_, and $Ri_{\mathrm{crit}}=\frac{1}{4}$.

The deposition of angular momentum is then given by the radial derivative of ℒ_*J*_. Given that, as a first approximation, only the radial dependency of IGW transport is considered, all quantities required are evaluated from horizontal (on isobars) averages. The angular momentum evolution due only with IGW transport is given by

8.36 $$\rho \frac{d}{dt}[r^{2}\Omega ]=\pm \frac{3}{8\pi }\frac{1}{r^{2}}\frac{\partial L_{J}(r)}{\partial r}.$$

The + or − sign in front of the angular momentum luminosity corresponds to prograde (*m*>0) or retrograde (*m*<0) waves.

**Figure 31.**
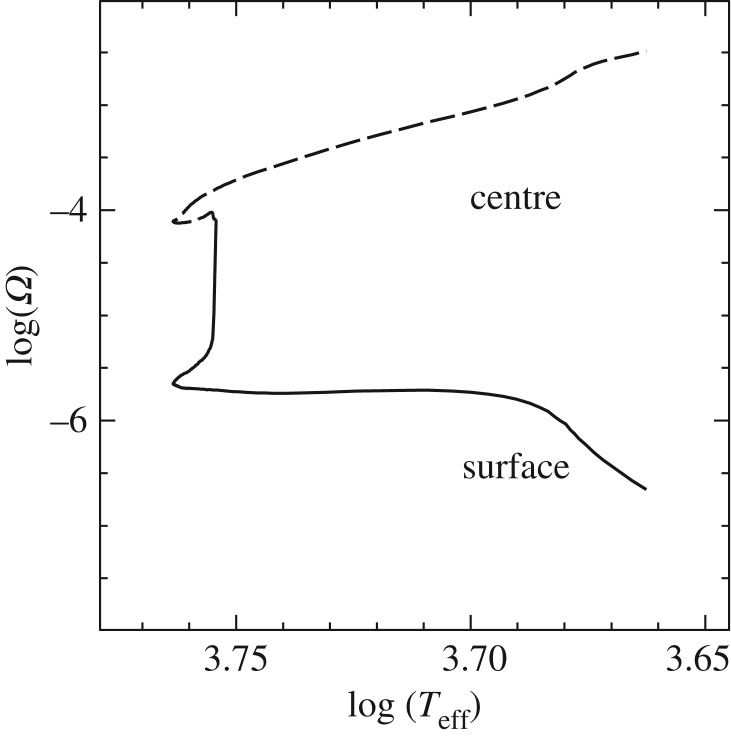
Evolution with *T*_eff_ of the central and surface angular velocity, for 1*M*_⊙_ initial solar chemical composition rotating models, calculated with the Geneva code [196].

It is clear that in the absence of differential damping for inward- and outward-travelling waves, there will be no angular momentum transport. This is the case, for example, of solid body rotation. However, differential rotation naturally filters the IGWs that propagate within the star. Considering, for example, the realistic case of a low-mass star with interior layers—see e.g. Figure 31 for the 1*M*_⊙_ solar chemical composition Geneva rotating model discussed before—that rotate faster than the convective envelope. In the inner radiative regions, we have *Ω*(*r*)>*Ω*(*c*) and for prograde *m*>0 waves *σ*=*ν*−*m*(*Ω*(*r*)−*Ω*(*c*)) is shifted to lower frequencies when travelling from the edge of the convective envelope towards the interior layers. The radial damping length of the waves given approximately by

8.37 $$l_{d}=\frac{2r^{3}\sigma^{4}}{{\left[l(l+1)\right]}^{3/2}NN_{T}^{2}K_{T}},$$

progressively decreases for these prograde waves. The retrograde *m*<0 waves are instead boosted to higher frequencies, and their damping length increases, propagating further within the star compared to the prograde waves. As a consequence, prograde waves are absorbed before they can propagate far into more rapidly rotating layers of a star, while retrograde waves pass through, and upon dissipation they deposit their negative angular momentum (they contribute with a negative sign in equation (8.36)), spinning down the rapidly rotating layers. The star then tends to evolve towards solid body rotation.

The angular momentum transport associated with IGWs is considered to be a possibility to explain the solar rotation profile, which is much flatter than generally predicted by rotating models that do not include IGWs [210]. Also, recent asteroseismic observations [211,212] show that the core of low-mass RGB stars do not rotate faster than the surface as much as predicted by current rotating stellar models. The angular momentum redistribution by IGWs may provide one way to reconcile theory with observations. Recent multidimensional detailed hydrodynamical simulations of generation and propagation of IGWs within stars, confirm their ability to transport efficiently angular momentum [213,214].

#### 8.3.2. Magnetic fields

Another way to favour the redistribution of angular momentum within a rotating star is to consider the effect of internal magnetic fields. They are generally implemented following the dynamo mechanism presented in [215], which envisages the creation of magnetic fields in the radiative regions of differentially rotating stars at the expense of the shear, due to the so-called *Tayler–Spruit* instability.^26^

The STERN code, which uses a diffusive implementation of element and angular momentum transports, accounts for the effect of magnetic fields as follows [217].

Defining by q=d ln⁡Ω/d ln⁡r the shear, the effective radial viscosity produced by the magnetic field can be written as

8.38 $$\nu_{re}=\frac{\nu_{e0}\nu_{e1}}{\nu_{e0}+\nu_{e1}}f(q),$$

where

8.39 $$\nu_{e0}=r^{2}\Omega q^{2}{\left(\frac{\Omega }{N_{\mu }}\right)}^{4}$$

and

8.40 $$\nu_{e1}=r^{2}\Omega \max\left[{\left(\frac{\Omega }{N_{T}}\right)}^{1/2}{\left(\frac{\kappa }{r^{2}N_{T}}\right)}^{1/2},q^{2}{\left(\frac{\Omega }{N_{T}}\right)}^{4}\right],$$

Denoting by *q*_min_ the minimum rotational gradient necessary for the dynamo to operate,

8.41 $$f(q)=1-\frac{q_{\min}}{q},\;(q>q_{\min})$$

and

8.42 $$f(q)=0,(q\leq q_{\min}).$$

The effective diffusivity for transport of chemical elements is given as

8.43 $$D_{e}=\frac{D_{e0}D_{e1}}{D_{e0}+D_{e1}}f(q),$$

where

8.44 $$D_{e0}=r^{2}\Omega q^{4}{\left(\frac{\Omega }{N_{\mu }}\right)}^{6}$$

and

8.45 $$D_{e1}=r^{2}\Omega \;\max\left[{\left(\frac{\Omega }{N_{T}}\right)}^{3/4}{\left(\frac{K_{T}}{r^{2}N_{T}}\right)}^{3/4},q^{2}{\left(\frac{\Omega }{N_{T}}\right)}^{6}\right].$$

According to [215] *q*_min_ is given by

8.46 $$\begin{array}{r} q_{\min}=q_{0}+q_{1} \end{array}$$

8.47 $$\begin{array}{r} q_{0}={\left(\frac{N_{\mu }}{\Omega }\right)}^{7/4}{\left(\frac{\eta }{r^{2}N_{\mu }}\right)}^{1/4} \end{array}$$

8.48 $$\begin{array}{r} \;\text{and}\;q_{1}={\left(\frac{N_{T}}{\Omega }\right)}^{7/4}{\left(\frac{\eta }{r^{2}N_{T}}\right)}^{1/4}{\left(\frac{\eta }{K_{T}}\right)}^{3/4}, \end{array}$$

with $\eta =7\times 10^{11}\ln(\Lambda )T^{-3/2}$ denoting the Spitzer magnetic diffusivity and ln⁡(Λ) the Coulomb logarithm (see §6).

These viscosities and diffusivities have been included as additional terms to the diffusion coefficients of, respectively, angular momentum and chemicals, in both the STERN [204,217] and KEPLER [218] codes, which use a diffusive implementation of element and angular momentum transports. In this latter code, modifications are applied to the viscosities and diffusivities derived before in the case of semiconvective regions, or where the thermohaline mixing is efficient, which means in regions where the timescale of mixing is much longer than in convective layers. In the case of semiconvection, a dynamo effective viscosity is defined as *ν*_re_=*ν*_*e*0_*f*(*q*) and the final expression for the viscosity in the semiconvective region is set to be $\nu_{e}=\sqrt{\nu_{re}\nu_{\mathrm{SC}}}$, where *ν*_SC_=(1/3)*H*_*p*_*v*_conv_ and *v*_conv_ is the convective velocity derived from the MLT. The effective diffusion coefficient for the transport of elements is set to (*D*_e_+*D*_SC_), with *D*_SC_ being the diffusion coefficient due to semiconvection. In the thermohaline mixing region *D*_e_ is set to *D*_*e*1_, *ν*_e_ to *ν*_*e*1_ and $q_{\min}$ to *q*_1_.

**Figure 32.**
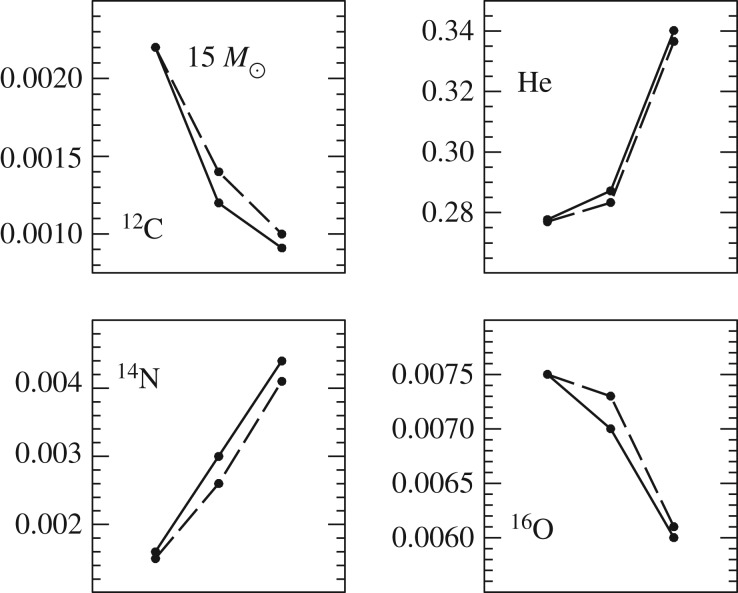
In each panel, from left to right, the abundances (mass fractions—filled circles) correspond to the MS stage when the central H mass fraction is 0.35, to central He-burning with a 0.50 mass fraction of He and to the pre-supernova stage. Solid lines correspond to rotating models without magnetic fields and dashed lines to rotating models with magnetic fields (see text for details).

The inclusion of internal magnetic fields decreases the rotation velocity contrast between core and envelope during the MS, approaching near solid body rotation. This affects both the shape of the evolutionary tracks as well as the abundance profiles within the models. Figure 32 displays, as an example, the effect on the surface chemical abundances at selected evolutionary stages in the calculations with the code KEPLER [218], for 15*M*_⊙_ solar metallicity models with a ZAMS equatorial rotation velocity of 200 km s^−1^.

Internal magnetic fields generated by the Tayler–Spruit instability have been also included in the Geneva code [219]—which implements the advective/diffusive formalism for rotational transports—in a slightly different form. Denoting by *x* the ratio

$$x={\left(\frac{\omega_{A}}{\Omega }\right)}^{2},$$

where *ω*_A_ is the Alfvén frequency of a magnetic field of intensity *B*, the solution of the equation

8.49 $$\frac{r^{2}\Omega }{q^{2}K_{T}}(N_{T}^{2}+N_{\mu }^{2})x^{4}-\frac{r^{2}\Omega^{3}}{K_{T}}x^{3}+2N_{\mu }^{2}x-2\Omega^{2}q^{2}=0$$

provides the unknown quantity *ω*_A_. The diffusion coefficient for the vertical transport of angular momentum is then given by

8.50 $$\nu_{re}=\frac{\Omega r^{2}}{q}{\left(\frac{\omega_{A}}{\Omega }\right)}^{3}\left(\frac{\Omega }{N}\right),$$

and the diffusion coefficient for the transport of chemicals by

8.51 $$D_{e}\;=\frac{r^{2}\Omega }{q^{2}}{\left(\frac{\omega_{A}}{\Omega }\right)}^{6}.$$

Once *ω*_A_ is calculated, the condition on the minimum shear for the dynamo to work is tested as

8.52 $$q>{\left(\frac{N}{\Omega }\right)}^{7/4}{\left(\frac{D_{e}}{r^{2}N}\right)}^{1/4}.$$

If this condition is not realized, a formalism for the treatment of low rotation rates—expanding upon work in [215]—is implemented. We notice that, in this treatment, the Brunt–Väisälä frequency is rewritten as

8.53 $$N^{2}=\frac{D_{e}/K_{T}}{(D_{e}/K_{T})+2}(N_{T}^{2}+N_{\mu }^{2}),$$

to account for the presence of a magnetic diffusivity *D*_e_.

A set of 15*M*_⊙_ solar metallicity models calculated with this implementation [219] also predict an almost solid body rotation during the MS, with obviously a negligible element transport by shear mixing. The transport of elements due to the magnetic fields is also negligible, while the meridional circulation is strongly enhanced by the flat rotational profile. The net effect during the MS is an enhancement of the surface abundance variations compared to rotating models without internal magnetic fields. This is different from the result of figure 32 for a KEPLER model with the same mass, initial chemical composition and very similar initial rotation rate. In this latter case, the variation of the surface abundances during the MS is almost negligible compared to the case without magnetic fields.

Finally, we mention very briefly the effect of magnetic braking due to magnetized winds.^27^ Winds with magnetic fields—in the case of surface magnetic fields, whatever their origin is—exert a braking torque that is significantly larger than that for non-magnetic cases [220,221]. The reason is that the magnetic field connects the mass lost from the surface of the star to the envelope, and when the wind finally decouples from the magnetic field, it has the same angular velocity as the surface but a much larger moment of inertia. This increases the amount of angular momentum lost, compared to the case with no magnetic fields, and it is accepted as the reason for the slow rotation rate of low-mass MS stars. The effect on the chemical element transport is indirect, through the variation of the star rotational profile. Surface magnetic fields are observed mainly in low-mass stars with convective envelopes, where they are expected to be generated by a dynamo mechanism, and this type of magnetic braking is implemented mainly in models of low-mass stars. There are various prescriptions in the literature that require the calibration of free parameters on observations, given the lack of knowledge about the surface magnetic fields [222]. For example, the rate of angular momentum loss due to magnetized winds employed in [222] is a variation of [223,224]:

8.54 $$\frac{dJ}{dt}=-K_{W}\Omega \Omega_{\mathrm{sat}}^{2}{\left(\frac{R}{R_{⊙}}\right)}^{1/2}{\left(\frac{M}{M_{⊙}}\right)}^{-1/2}\;\mathrm{for}\;\Omega \geq \Omega_{\mathrm{sat}}$$

and

8.55 $$\frac{dJ}{dt}=-K_{W}\Omega^{3}{\left(\frac{R}{R_{⊙}}\right)}^{1/2}{\left(\frac{M}{M_{⊙}}\right)}^{-1/2}\;\mathrm{for}\;\Omega <\Omega_{\mathrm{sat}},$$

where *K*_W_=2×10^48^ in cgs units to reproduce the solar case and *Ω*_sat_ is a free parameter.

## 9. An example of possible synergy among several element transport processes

**Figure 33.**
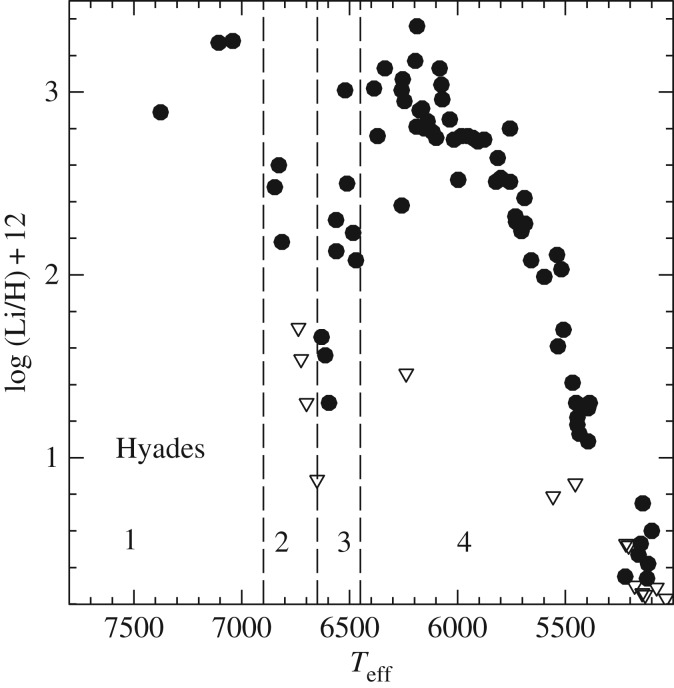
Li abundances measured in a sample of Hyades MS stars, as a function of their *T*_eff_ [225,226]. Inverted open triangles denote upper limits. The four different regions marked in the diagram are discussed in the text.

After the description of all major element transport mechanisms included in modern stellar evolution calculations, we show just an example of how their synergy might explain some puzzling observations of chemical abundances in star clusters. Figure 33 displays the trend of the surface Li abundance as a function of *T*_eff_ for a sample of MS stars in the approximately 600 Myr old Hyades open cluster [225,226]. It is easy to notice the so-called *Li-dip* around 6600 K, a sharp local minimum of the Li abundance, which cannot be explained by standard stellar evolution models including only convective mixing (the Li-dip is observed also in other open clusters of different ages, at similar temperature).

Lithium is a very fragile element that is burned at temperatures of approximately 2.5×10^6^ K. In solar metallicity MS models calculated with just convective mixing, and *T*_eff_ between approximately 6400 and 7500 K, the Li burning temperature is well below the base of the convective envelope, hence the Li abundance is constant down to the radiative layers where the temperature reaches approximately 2.5×10^6^ K. This implies that these models—which also are not expected to experience pre-MS Li depletion—predict a constant surface Li with *T*_eff_ in this temperature range, contrary to observations.

To explain these observations of Li abundances, we outline below the scenario proposed by Talon & Charbonnel [227], which involves the combination of several of the element transport mechanisms discussed above.^28^ To this purpose, we have divided the temperature range covered by the data of figure 33 into four contiguous ranges:

(i) *Region* 1. These stars have shallow convective envelopes, not efficient at generating a surface magnetic field via a dynamo process, hence the surface is not slowed down by magnetic winds. As a result, rotational mixing is expected to be just about sufficient to counteract atomic diffusion of Li below the convective envelope (e.g. counteracts the creation of a Li gradient right below the convective boundary where Li diffused from the convective region accumulates).
(ii) *Region* 2. Moving towards lower temperatures, the convective envelopes deepen, and dynamo-generated weak surface magnetic fields are expected now to start slowing down the outer layers. The increased shear strenghtens the rotational mixing and Li depletion increases with decreasing *T*_eff_. This happens because the more vigorous mixing is trying now to erase the gradient between the Li-burning region (no Li) and Li-rich convective layers.
(iii) *Region* 3. Stars on the cool side of the Li-dip have even deeper convective envelopes, that sustain a very efficient dynamo and slow down even more the external layers. At the same time, these convective layers are now very efficient at generating IGWs that redistribute momentum (driving the star towards solid body rotation) and reduce the efficiency of rotational mixing, inducing an increase in surface Li with decreasing *T*_eff_. Calculations based on the approximations described before [227] show that the expected efficiency of IGW-induced angular momentum transport has the required dependence on stellar mass (*T*_eff_).
(iv) *Region* 4. At these low *T*_eff_, the convective envelope is deep enough to reach Li burning temperatures, causing an increasingly larger depletion with decreasing *T*_eff_.

Measurements of projected rotation velocities in this cluster display a decreasing average velocity with decreasing *T*_eff_, consistent with an increased efficiency of magnetic braking when moving towards lower stellar masses [229].

## 10. Conclusion

It is clear that the description of complex physical processes like turbulent convection, semiconvection, thermohaline mixing and rotation, implemented in stellar evolution calculations is necessarily simplified, with the predictive power in some cases hampered by the use of several free parameters of uncertain calibration. Also, the effect of interactions among the various instabilities in rotating stars—which usually are considered as independent—has to be fully explored [230].

It is also fair to say that, in many cases, the approaches used in stellar evolution models are probably reaching their limits, and further developments of multidimensional hydrodynamical simulations are crucial to progress in the field. As we have discussed, there are already some constraints that current hydrodynamical simulations pose to the element transport processes efficient in stars. Even though there is clearly still a long way to go to complete stellar interior modelling with numerical hydrodynamics, physical insights provided by computer simulations are invaluable in improving our understanding of element transport processes [3,231]. Just to give two examples, recent results from hydrodynamical simulations provide new indications about the way to go to replace the MLT in stellar modelling [232], as well as how to implement a more consistent description of overshooting in different regimes [40].

At the same time, the booming field of asteroseismology is starting to provide new information on the efficiency of element transport processes, that can at the very least be used to add further constraints to our current recipes. Two perfect examples are recent works on mixing in low-mass core He-burning stars [233,234], and angular momentum transport in RGB stars [235,236].

The hope is that a synergy between clues from hydrodynamical simulations and the powerful constraints coming from asteroseismic analyses will help to improve our description of element transport in stellar model computations and improve their predicting power.

## Footnotes

1 For example, main sequence core convection of massive short-lived models involves ≈10^9^ turnover times to complete the exhaustion of fuel. The Reynolds number in stars is *Re*>10^9^–10^10^, much larger than the effective Reynolds number, approximately 10^4^ in the current best-resolved simulations.

2 In the case of pre-supernova fast evolutionary phases, the equation of hydrostatic equilibrium usually contains an extra term $-(\frac{1}{4}\pi r^{2})(\partial^{2}r/\partial t^{2})$ called the *acceleration term*.

3 If *A* and *b* are identical particles, the factor *n*_A_!*n*_b_! has to be replaced with (*n*_A_+*n*_b_)!.

4 A term −*X*_*s*_/*τ*_d_ has to be added to the right-hand side of equation (2.7) if element *s* decays with decaying constant *τ*_d_.

5 The values assumed in the classical and widely employed MLT flavour [17] are $a=\frac{1}{8}$, $b=\frac{1}{2}$ and *c*=24. Solar models calculated with this choice of the MLT flavour usually require *α*_MLT_ of the order of 1.6–2.0, the exact value depending on the specific physics inputs and treatment of the outer boundary conditions in the calculations. Remarkably enough, different choices of these three parameters found in the literature provide the same results when *α*_MLT_ is recalibrated on the Sun.

6 But see also [24] who claim that *α*_MLT_ should vary with stellar properties, using data from the *Kepler* satellite.

7 Another calibration of *α*_MLT_ from an independent suite of 3D radiation hydrodynamics simulations of stellar envelopes [27] has not been implemented yet in stellar evolution calculations.

8 Semiconvection can also be important during Si-burning [65] in massive stars.

9 Even a difference in rotational velocity between the convective core and the overlying radiative layers can have a similar effect. In this case, equation (4.5) becomes $z=v_{\mathrm{tang}}^{2}/(4g(1-\mu_{1}/\mu_{2})),$ where *v*_tang_ is now the difference in rotational velocity between the convective core (expected to rotate like a solid body) and the overlying radiative layers. Equation (4.6) becomes $v_{p}=(v_{\mathrm{tang}}^{2}v_{\mathrm{mix}})/(4g\Lambda (1-(\mu_{1}/\mu_{2})))$

10 Even the current uncertainties in the ^12^C(*α*,*γ*)^16^*O* reaction rate can affect the CO profiles, and the HB/AGB star count ratio.

11 Thermohaline mixing due to the same reasons as along the RGB is also efficient during the HB and AGB evolution of low-mass stars, according to some calculations [91]. An upper limit of 1.5*M*_⊙_[91] or 2.2*M*_⊙_[92] is found for RGB stars (the same 1.5*M*_⊙_ limit is found by Cantiello & Langer [91] for HB and AGB stars) to develop thermohaline mixing. The efficiency of the mixing in general tends to decrease with increasing mass. Thermohaline mixing is also important when carbon has ignited off-centre in the core of super-AGB stars, for it affects significantly the propagation of the flame [93].

12 About 5% of the post-bump RGB objects investigated do not show these abundance variations. A strong magnetic field can potentially inhibit thermohaline mixing [96].

13 The calculations by Cantiello & Langer [91] and the MESA calculations by Lattanzio *et al*. [101] consider the constant *C*_th_ as $C_{\mathrm{th}}\equiv (\frac{3}{2})\alpha_{\mathrm{th}}$, where *α*_th_ is in this case the free parameter.

14 We discussed in §4 the effect of diffusion on the HB mixed cores.

15 The surface abundance variations tend to increase the envelope opacities which, in turn, would tend to increase the MS lifetime. But the dominant process is the decrease of available fuel, and the net effect is an approximately 10% decrease of the MS lifetime.

16 Metals observed in a minority of WD spectra are ascribed to the effect of accretion.

17 If diffusion is inhibited only from the outer convective layers of low-mass stars, the effect on the interiors can still affect the age determination of clusters and field stars [138].

18 The He settling velocity *w*_He_ can be related to a diffusion coefficient *D*(He) through

$$D(\mathrm{He})∼\frac{K_{B}Tw_{\mathrm{He}}}{m_{\mathrm{He}}g},$$

where *m*_He_ is the mass of the He nucleus.

19 It may seem surprising to be able to calculate 1D rotating models, given that the centrifugal forces reduce the effective gravity according to the latitude and introduce deviations from spherical symmetry.

20 Hydrodynamical simulations show that shear mixing is already efficient for values of *Ri* higher than $\frac{1}{4}$[186].

21 The Solberg–Høiland criterion for stability is essentially the Ledoux criterion when rotation is present. For a moderate outward decrease of the angular velocity, rotation favours stability compared to the Ledoux criterion.

22 From now on, for simplifying the notation, we will denote by *r* the radius of the isobar *r*_*P*_.

23 The model with rotation is not calculated up to the tip of the RGB, but from independent calculations with rotation we know that He-core masses at He-ignition are larger in rotating models. This causes a brighter tip of the RGB and a brighter HB luminosity compared to the non-rotating case.

24 See also [202] for a similar analysis.

25 The total angular momentum luminosity is defined as the average angular momentum flux transported by IGWs through a surface of radius *r*.

26 The efficiency of this mechanism is, however, questioned by recent numerical simulations [216].

27 The complex angular momentum evolution during the pre-MS phase due to influence of the surrounding disc, is generally not modelled by stellar evolution calculations.

28 Other scenarios to explain the Li-dip are described in [228].

## Data accessibility

This article has no additional data.
